# Uncovering the fast, directional signal flow through the human temporal pole during semantic processing

**DOI:** 10.1038/s41598-023-33318-5

**Published:** 2023-04-26

**Authors:** P. Tiesinga, A. Platonov, V. Pelliccia, G. LoRusso, I. Sartori, G. A. Orban

**Affiliations:** 1 Neuroinformatics Department, Faculty of Science, Radboud University, Heyendaalseweg 135, 6525AJ Nijmegen, The Netherlands; 2 Department of Medicine and Surgery, University of Parma, Via Volturno 39/E, 43125 Parma, Italy; 3 Claudio Munari Center for Epilepsy Surgery, Ospedale Niguarda-Ca’ Granda, 20162 Milan, Italy

**Keywords:** Cognitive control, Decision, Neural decoding, Data processing

## Abstract

The temporal pole (TP) plays a central role in semantic memory, yet its neural machinery is unknown. Intracerebral recordings in patients discriminating visually the gender or actions of an actor, yielded gender discrimination responses in the ventrolateral (VL) and tip (T) regions of right TP. Granger causality revealed task-specific signals travelling first forward from VL to T, under control of orbitofrontal cortex (OFC) and neighboring prefrontal cortex, and then, strongly, backwards from T to VL. Many other cortical regions provided inputs to or received outputs from both TP regions, often with longer delays, with ventral temporal afferents to VL signaling the actor’s physical appearance. The TP response timing reflected more that of the connections to VL, controlled by OFC, than that of the input leads themselves. Thus, visual evidence for gender categories, collected by VL, activates category labels in T, and consequently, category features in VL, indicating a two-stage representation of semantic categories in TP.

## Introduction

**Figure 1:**
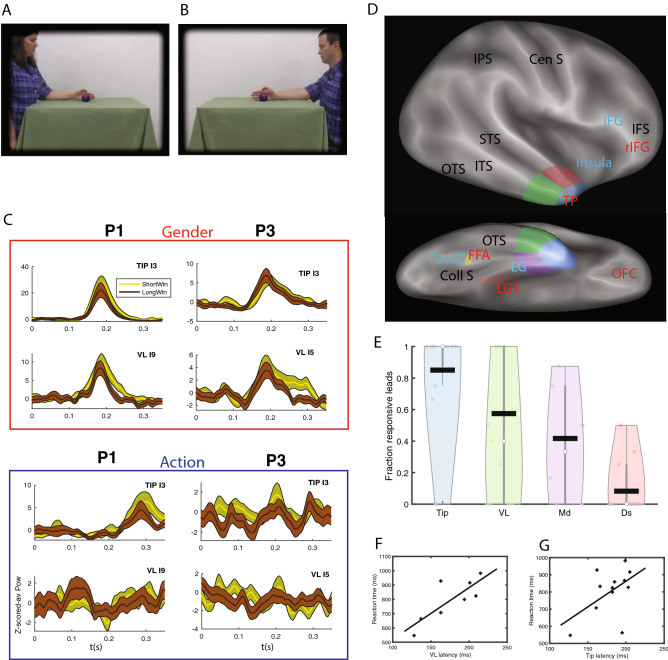
Functional heterogeneity of the TP. (**A**), (**B**): static frames of the video showing the two actors; (**C**): known properties of TP responses: brief phasic responses (wavelet power in gamma band, the mean across trials is z-scored using the spontaneous activity prior to static onset) in (top panels) gender task are similar for short (yellow) and long (brown) stimuli, which are absent in the (bottom panels) action task. We show time courses of power from static onset for example leads (inset) in T and VL for two patients (P1 and P3). Shaded bands represent standard deviation across twenty bootstraps. (**D**): Lateral and ventral views of the unfolded right hemisphere indicating the four parts of TP: dorsal (Ds, red), ventrolateral (VL, green), medial (Md, purple) and tip (T, blue): border between ventrolateral and dorsal or medial in the depth of the STS and OTS respectively; border between ventrolateral and tip at the end of the OTS and STS; the areas that provide the strongest input to TP for the task are labeled in red, these are LG1 (red ellipse), FFA (yellow ellipse), OFC and rIFG. (**E**): proportion of responsive leads in the four subregions: mean: horizontal line, median open circle, dots individual patients; (**F**), (**G**): Reaction time (RT) as a function of latency (‘lat’) of leads in VL (F, 8 patients) and Tip (G, 12 patients); Linear fit: RT: 3.6 lat + 161 ms (F) and RT = 3lat + 263 ms (G) but correlation significant in F (r = 0.80, *p* < 0.02) and not in G (r = 0.51, *p* > 0.05). *STS* superior temporal sulcus, *ITS* inferior temporal sulcus, *OTS* occipito-temporal sulcus, *Coll S* collateral sulcus, *IPS* intraparietal sulcus, *Cen S* central sulcus, *IFS* inferior frontal sulcus, *FG* fusiform gyrus, *LG* lingual gyrus, *IFG* inferior frontal gyrus, *OFC* orbitofrontal cortex, *rIFG* rostral inferior frontal gyrus.

Previous neuropsychology and imaging studies have established that the temporal pole (TP), located at the rostral end of the human temporal lobe, plays a crucial role in semantic memory^1^, but how the TP achieves this function remains unknown^2–4^, due to the lack of direct electrical recordings in TP. However, a growing number of studies have shown that TP is not an homogeneous region, but that it contains multiple cytoarchitectonic areas^5^, which have been grouped functionally using functional connectivity measured with fMRI^3,4,6,7^. This heterogeneity of TP allows some questions about its operations to be formulated. One question concerns the flow of information in TP. Since some of the subparts are unimodal and others multimodal, one could expect signals to flow first from uni- to multimodal regions. Another question concerns the precise anatomical identity of the semantic hub: it is unclear whether the hub corresponds to the whole of TP or even the two TPs^1^, or to one or two subparts of TP^8^. If the hub were to correspond to a single subpart, this raises the problem of how a small brain region can represent the wide range of semantic knowledge. To begin to address these questions, we build on our previous stereo EEG study^9^ (Methods, in “Patients”–”Standard statistical analysis” sections) showing that TP, more particularly the right TP, was specifically activated when patients visually discriminated the gender rather than the action performed by an actor (Fig. 1A,B). Indeed object concepts, and semantic categories in general, are basic elements of semantic memory^10,11^, and deciding whether a visual test (here the image of a person) belongs to a semantic category (here male or female, subordinate levels of humans) is a typical way to test representations of such semantic categories^10^. Thus we used the gender discrimination task of Platonov et al.^9^ to investigate the representation of the semantic category gender in human TP. Hence, we concentrated on those patients tested with the Platonov et al. tasks^9^, in which right TP was well explored by stereo EEG recordings, to investigate the broadband gamma/high gamma responses (50–150 Hz, referred to as the broadband gamma in the following) in the different parts of TP. Importantly, we complemented this analysis by computation of Granger causality^12,13^ between recorded leads to trace the directional flow of information within the TP and between TP and outside cortical regions.

## Results

### Functional heterogeneity of TP

The stimuli and tasks were exactly the same as in^9^ (see Methods, in “Behavioral testing” section). Patients viewed a screen on which a video, picturing a female or male actor performing one of two manipulative actions, was shown preceded by a static presentation of the first frame for a variable time (275–875 ms). In a two by two design either the complete video or a truncated one was shown (see Methods, in “Behavioral testing”), and patients either decided about the gender (using the static presentation) or discriminated the action performed (requiring the video). In a large group of 31 patients (Methods, in “Patients”), in which stereo EEG recordings (Methods, Section “StereoEEG data recording” section) in TP were available, 102/176 (58%) leads in right TP were responsive in the gender task (Fig. 1A, B), compared to 31/76 (39%) in left TP, confirming^9^. We therefore focused the present analysis on 19 out of these 31 patients with responsive leads in right TP, and on the trials in which a complete video of a manipulative action was shown. The 19 patients (8 females, age 21–49 y, Tables S1, S2) performed the gender decision task well (median accuracy 98%, median RT 827 ms after static onset). As in^9^, we found that TP leads show in the gender task brief, task-specific increases in broadband gamma (see Methods, in “Standard statistical analysis” section), which depended little on the duration of the static stimulus (Fig. 1C, top panels).

To investigate the functional heterogeneity of TP, we grouped the different architectonic areas of TP (5) into four subregions (Fig. 1D), following^6^. Specifically, we define a dorsal (Ds) subregion, matching the anterior TA cytoarchitectonic area and connected with auditory and somatosensory cortex^6^. We, however, considered cytoarchitectonic areas TG and anterior TE as separate subregions, labelled Tip (T) and ventrolateral (VL) respectively, because (1) TG in^6^ was the only subregion connected to all other TP subregions, indicating multimodality, while anterior TE was clearly visual in nature, as demonstrated by the connections with visual pulvinar, and (2) TG is dysgranular, while anterior TE is granular^5^. Finally, we grouped the remaining areas, rhinal cortex (areas 35, 36), enthorinal cortex, and TI, which all are agranular or dysgranular^5^, into a single ‘Medial’ (Md) subregion (Fig. 1D). The location and extent of these four TP subregions (Fig. 1D) was estimated from the location of the corresponding TP parts on the coronal sections of TP, shown in Fig. 1 of reference^6^. Of these four subregions, T is inferred from^6^ to be multimodal and VL only visual, something we cannot discriminate with the visual task we used. The proportions of responsive leads varied significantly (mixed effects, see Methods, in “Statistical tests” section and Suppl Text 1) between the four TP parts, being largest in T (85%), second in VL (57%), third in Md (42%) and smallest in Ds (8%, Figs. 1E, S1-1). While responsive leads are simply active during the static presentation in the gender task, a subset of these responsive leads is labelled selective when they are active during the static presentation but not during the video presentation in the gender task, nor during the static presentation in the action task. The proportion of selective leads showed similar significant (mixed effects, see Suppl Text 2) variations across the four TP parts, ranging from 58% of tested leads (T) to 8% (Ds, Fig. S1-1).

Responses in T were modestly stronger and longer, and had slightly shorter latencies than those in VL (see Table 1). In agreement with^9^, we found the RT to increase with response latency in VL and T rather similarly, but the correlation reached significance only in VL (Fig. 1F, G; VL: r = 0.80, *p* < 0.02, T: r = 0.51, *p* > 0.05). Although the number of patients contributing to the relationship for Md was small, this relationship was radically different in Md, with RT decreasing with increasing latency (RT = 939 ms-0.95 Lat), and the negative slope of Md falling outside the confidence interval of the slope for VL. To visualize the suppression in the action task we averaged (Fig. S1-2) the time courses of the leads of each TP part^9^, and observed only in T and VL a long lasting suppression in the action task, including both the static interval and the whole video presentation (Fig. S1-2). Thus our results bear out the functional heterogeneity of TP, with T and VL contributing heavily to the gender task, and T being more responsive and VL more related to the behavior. Although a substantial fraction of the tested TP leads (Black bar, 71/170 = 42%, Fig. S1-3A) were located in the epileptic zone (EZ), this impacted little our results, as a similar proportion of EZ leads occurred in the four regions, and both the proportion of responsive leads and the response strength were similar in and outside the EZ (Fig. S1-3B).

**Table 1:** Median (across patients) response properties of four subregions.

Subregion	Number of patients	Median Response (z-score)	Median Latency (ms)	Median Duration (ms)
Tip	14	1.04	182	113
Ventrolateral	8	0.85	178	97
Medial	4	0.66	201	85
Dorsal	3	0.45	165	58

**Figure 2:**
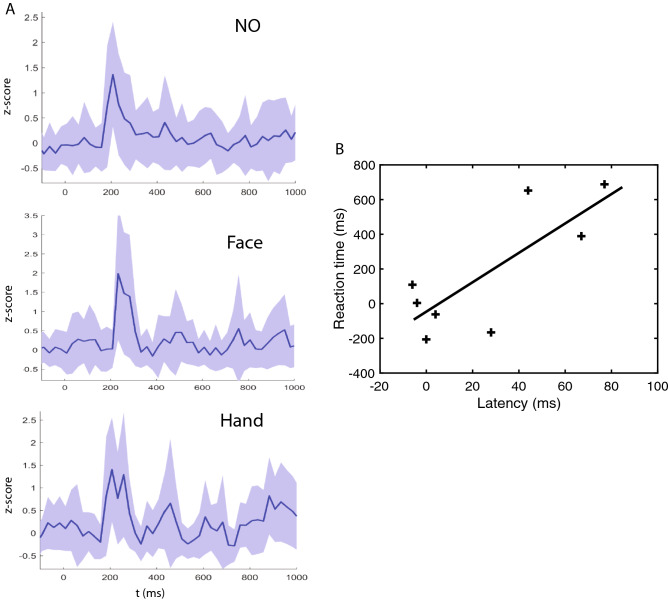
Masking effects. (**A**) responses of TP leads (VL and T averaged) to static onset in gender task of patient P1 without mask (no), face masked (face) or hand masked (hand); (**B**) Increase in RT with masking as a function of increase in median latency of TP responses across 4 patients (P1, 2, 3, 7); DeltaRT: 46 ms + 8.5 DeltaLat, correlation: r = 0.79, *p* < 0.02. The latency was determined as the time the z-scored response crossed a threshold of 3SD relative to baseline.

In the videos both the face and the body of the actor were visible. Hence, to determine the source of the TP gender signals, we masked either the face or the hand (Fig. S2-1A) in four of the 19 patients (Table S1, Method in “Behavioral testing” section), and compared the responses in the gender task to that without masking. TP lead responses (averaging over VL and T leads) were similar in strength in all three conditions (Fig. 2A), but responses were delayed only when the face was masked, this delay correlating significantly (r = 0.79, *p* < 0.02) with an increase in RT (Fig. 2B), consistent with the increase in RT with latency reported earlier for the unmasked videos (Fig. 1F, G). In one patient (patient 2) we could verify that masking effects were similar in T and VL (Fig. S2-1B). Thus both body (hand) and face signals contributed to the gender decision responses in TP, face signals being faster than hand signals.

### Intrinsic functional connections of TP

**Figure 3:**
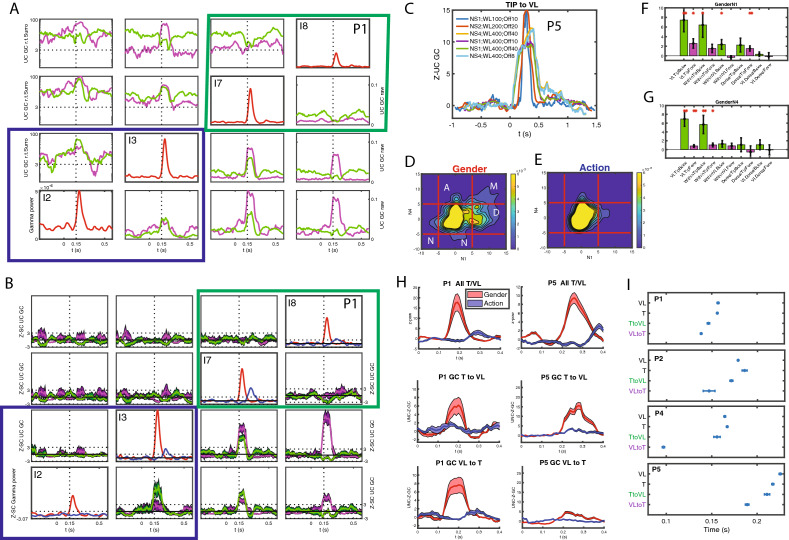
Functional connections inside TP. (**A**) Lower diagonal: Raw unconditioned GC (uGC) between leads in Tip (blue box: I2-I3) and in VL (green box I7-8) parts of TP in patient P1 for the gender task in the forward (towards the rostral end of TP, purple) and backward direction (away from the rostral end, green). Note: purple in general indicates from the column lead to row lead, green in the opposite direction. The upper diagonal represents the uGC normalized by subtracting the mean of the surrogate data and dividing by the standard deviation, plotted on a log-scale. Dotted horizontal lines indicates z = 3. The color convention is inverted, meaning the purple goes from row lead to column lead, hence purple again indicates forward direction. On the diagonal: responses of the leads to static onset in gender task on the same timescale (0 = static onset). (**B**) same data as in A, but z-scored (by period before stimulus onset) unconditioned GCs with confidence limits obtained by bootstrapping (shaded band); the upper diagonal shows the action task and below diagonal is the gender task. The diagonal has z-scored gender (red) and action (blue) task responses. The dotted line is z-score = 3. (**C**) time course of Tip-to-VL uGC in patient P5 at different maximum lags: 4 ms (NS1), 8 ms (NS2) and 16 ms (NS4) and for different window lengths and offsets as indicated in the inset: NS indicates the sampling interval, WL the window length in ms, off is the shift in ms for moving the window over the trial interval. Thus shift size has little effect, while increasing window length broadens the peak. Comparing similar windows (400 ms) show a slightly stronger NS4 uGC (orange) compared to NS1 uGC (green). (**D**), (**E**) distribution of NS1 (abscissa) and NS4 ( ordinate) uGCs between leads in different parts of TP in gender (**D**) and action (**E**) task; the letters in D indicate the types of functional connection: disappear (D), maintain (M), appear (A), and negative (N) (see Methods). (**F**), (**G**) distribution of mean (mixed effect corrected, see Suppl Text 3) NS1 (F) and NS4 (G). Vertical lines indicate SE, one asterisk is *p* < 0.05; two asterisks *p* < 0.01. (**H**) Time course of NS1 uGC from VL to T (middle) and from T to VL (lower) compared to that of power in TP (upper) of P1 (left) and P5 (right) in gender (red) and action (blue) task (shading represents SE); (**I**) mean (± SE) onset latencies (bootstrap) of NS1-uGC from VL to T and from T to VL relative to that of power in VL and T, for four patients. In all four patients onset VL-T is significantly earlier than onset T-VL.

We have so far shown that T and VL are the two main TP regions involved in the use of visual information to decide about the gender of the actor. Next, we used Granger causality^12,13^ (see Methods, in “Granger causality analysis” section ) to trace the information flow within and between these two regions, and compared them to their functional connections with the dorsal region. This analysis was performed in 7 of the 19 patients (Table S1). These included the four more recently recorded patients, also tested with masking, because the quality of their recordings reduced the need for artefact rejections (see Methods, in “Granger causality analysis” section). The 3 remaining patients were part of the previous study^9^, but had large numbers of leads in TP (13 or more). When computing the unconditioned Granger causality (uGC) in the time domain (see Methods, in “Granger causality analysis” section), we observed both tonic and phasic components (Fig. 3A, S3-1), carried by broadband gamma frequency bands (Fig. S3-2). We used z-scoring to isolate the phasic components (Figs. 3B, S3-3), and restricted all further analysis to these phasic Granger causality components. To cover the range of possible connections, and their corresponding range of temporal delays, we investigated in addition to these uGC calculated for a short delay (sampling rate 1000 Hz, with n = 4 lags in the GC calculation, i.e. maximally 4 ms, referred to as NS1, see Methods, in “Granger causality analysis” section) also uGC at longer delays (sampling rate 250 Hz, also at n = 4 lags, hence now maximally 16 ms, denoted as NS4), and observed a similar but coarser time course than the short delays (Fig. 3C, S3-4). Given the phasic nature of the uGC changes, we defined the *strength* of the uGC as the extreme value in the interval 0.1 to 0.4 s after static onset. Both uGC strength distributions display a longer positive tail in the gender than action task (Fig. 3D,E, S3-5), but showed little (albeit significant) correlation, indicating largely independent measurements (Fig. S3-5).The NS1-uGC depended less on low frequencies (below 50 Hz, 1–10 Hz) than the NS4-uGC, at least for the gender task (Table S3). Both uGC depended little on the power of the source or target leads (Tables S4, S5, see Methods, in “Granger causality analysis” section), indicating they are an independent measurement. In the gender task, connections within T and between T and VL were clearly stronger than connections of T or VL with Ds (Fig. 3F, mixed effects, see Suppl text 3). Furthermore, both NS1 and NS4 connections within T and between T and VL were significantly stronger in the backward than the forward direction, and overall connections in gender task were stronger than in action (mixed effects see Suppl text 4, Fig. S3-6). Very similar results were observed for the strength of the NS4-uGCs (Fig. 3G, S3-6).

We next investigated how the strength of the uGCs at the two delays combined for a pair of TP leads. Given the skewness of the uGC distributions (Fig. S3-5), we used a threshold of 5 to define strong connections of either sign, and considered a value of 3 as threshold of medium connections. Combining positive strong connections for NS1 and NS4 yielded 3 types (disappear, maintain and appear, Fig. 3D), while leads showing a strong negative uGC for at least one delay were grouped in a fourth type, labelled negative. These four types differed between the five connections between TP regions (within-T, T-VL, within-VL, VL-Ds and T-Ds with both directions combined): the uGC types with strong NS1 components (disappear and maintain) dominated in the within-T and the VL-T connections (mixed effects, see Suppl Text 5, Fig. S3-6). They also differed between the two directions of the within-T, T-VL and within-VL connections (mixed effects, see Suppl Text 6). This suggests that especially in the backward direction, a substantial fraction of these connections between and within T and VL are direct connections.

The previous analysis indicated the strength of the VL-T connections, linking the two regions most responsive in the gender task. We next investigated the timing of the T-VL NS1 uGCs. This could only be investigated in 5 of the 7 patients with leads in both VL and T and with strong enough uGCs between the two regions (we required 3 strong connections in at least one direction). Remarkably, the time course of the NS1-uGC between VL and T (Fig. 3H) was very similar to that of the TP activation, and equally task dependent. Furthermore, in all four patients for which data were available (Fig. S3-8), the NS1-uGC increase in the forward (VL to T) direction started significantly earlier than that in the backward direction (Fig. 3I), even if the backward uGC was stronger (Fig. 3F). Thus visual signals flow first, as predicted, from unimodal VL to multimodal T, but then quickly and strongly in the opposite direction. This flow could not be resolved by the latency of the responses in VL and T, which were very similar.

**Figure 4:**
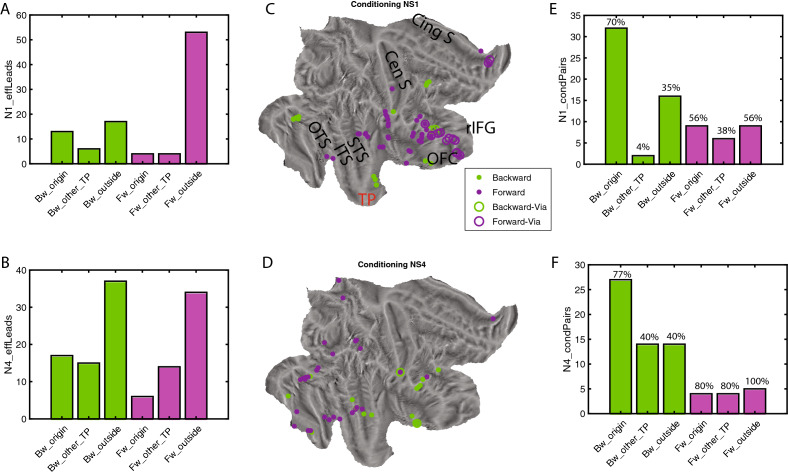
Conditioning of GC within TP: VL –T connection. (**A**), (**B**) number of leads with conditioning effect on GC from T to VL (Bw, green) and from VL to T (Fw, purple) for short delay (A, NS1) and long delay (**B**, NS4) in source TP region, other TP parts or outside TP. (**C**), (**D**) flatmaps of right hemisphere with location of outside leads having a conditioning effect on NS1 (**C**) and NS4 (**D**) uGC in forward direction (purple, via-leads: open circle) and backward direction (green, via leads: open circle). (**E**), (**F**) number of pairs in backward (Bw, green) and forward (Fw, purple) direction being conditioned by leads located in source TP region, other TP parts and outside TP for short (**E**) and long (**F**) delays.

Next, to investigate to what extent the uGC between VL and T represented direct connections, we tested whether conditioning with activity of a third lead (see Methods, in “Granger causality analysis” section) could reduce the NS1 or NS4 uGC of strong VL-T connections to values below 3 (less than medium strength connections). This analysis was performed on the same five patients used for the timing of the VL-T connections. For conditioning leads outside TP we defined ‘via-leads’ as those leads connected to both the TP source and target (source to via-lead and via-lead to target uGCs > 5). Non-via-leads are the remaining leads for which one or both of the uGC scores did not exceed 5. NS1 uGC in the forward VL to T connection were conditioned by a few leads within VL, but by many more outside TP (Fig. 4A). Those included via-leads, all in the PFC and OFC, and non-via leads centered on the insula and the opercular regions (Fig. 4C), all of which exerted an outside control on the short-delay VL to T connection. For the NS4-uGC in the same direction (Fig. 4B), the number of conditioning leads were more balanced between VL and outside TP, but the latter were all *non*-via and scattered over the cortex, including in temporal and parietal cortex. In the backward direction (T to VL) significantly (mixed effect, see Suppl Text 7 & 7bis) fewer leads outside TP had a conditioning effect on the NS1-uGC, compared to the forward direction, and very few of these were via leads (Fig. 4A). For the NS4-uGC backward connection more outside leads had a conditioning effect, and for both delays these outside leads were located in prefrontal and temporal cortex (Fig. 4C, D, see Methods in “Visualization of lead location” section). These results are consistent with the view that internal NS1 and NS4 uGCs represent chiefly direct and indirect connections, respectively. It is noteworthy that in the backward direction for both delays conditioning leads within the TP source had a much more widespread effect on uGCs than the outside leads (Fig. 4E, F). For the maintain leads, which exhibit both strong NS1 and NS4 connections, we could compare the leads having a conditioning effect. We found that only a minority such leads matched for the two delays (11% and 18% in forward and backward directions respectively), providing further evidence of the independence of these two functional connectivity measurements. Also connections within a subregion (backwards within Tip, Fig. S5-1) were conditioned by outside TP leads, for both NS1 and NS4 uGCs, to a degree similar to T to VL connections, but this could be investigated only in 3 patients. Thus for intrinsic TP connections, uGCs at short delays were most prominent and for these delays, the two directions of the VL-T connections differed widely, the forward direction being earlier and more under outside control, and the backward direction much stronger.

### Functional connections of TP with outside cortical regions

**Figure 5:**
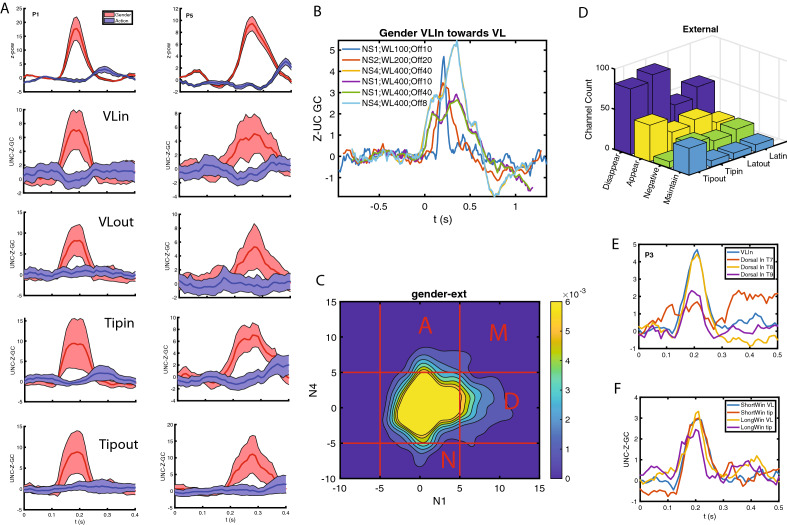
Functional connections of T and VL with outside cortical leads. (**A**) (Top panels) power of TP leads in gender (red) and action (blue) tasks for patient P1 (left) and P5 (right) compared to the time course of NS1 uGCs in both directions (continuing from top to bottom) with VL (VLin, VLout) and T (Tipin, Tipout); Full line mean, hatching 1STD; notice the shift to the right of all curves in P5 relative to P1. (**B**) time course of VLin uCG for 1 ms (NS1), 2 ms (NS2) and 4 ms (NS4) sampling times and max lag 4 samples in patient P5. Same conventions as Fig. 3C. (**C**) Joint distribution of NS1 (abscissa) and NS4 (ordinate) uGC linking T and VL with outside leads in either direction in gender task. Same conventions as in Fig. 3D. (**D**) Distribution of the four strong functional connection types for the four anatomical types of outside connections. (**E**) time course of NS1 uGC from VLin leads to 3 dorsal TP leads (red, yellow, purple) compared to average VLin NS1 uGC (blue) in patient P3. (**F**) time course of VLin (blue, yellow) and Tipin (red, purple) uGCs NS1 for short (blue, red) and long (yellow, purple) stimulus presentations also in P3.

We next investigated the in- and output connections of the leads outside TP with both the VL and T regions in the 5 patients in which VL-T timing was investigated (Table S1). Indeed, in these patients electrodes targeted the two TP regions, and timing of intrinsic and extrinsic connections could be compared. Each patient had over 100 leads outside TP and globally the 619 outside leads in the five patients covered the whole cortex well (Fig. S5-1), with the exception of occipital cortex, implanted in only one patient, and parietal cortex, explored only in its rostro-ventral part (Table S6). Being interested in the functional connections of outside leads with T or VL, we averaged the uGC over the different VL or T leads available in a given patient. Again we considered uGC at two delays (Fig. S5-2): 4 ms (NS1) and 16 ms (NS4), which showed phasic increases synchronously with the TP activation, as illustrated for the four NS1 uGCs in Fig. 5A, B. These uGC distributions for the two delays were more similar than for the intrinsic connections, but showed again little correlation (Fig. 5C, S5-3, r = 0.254 (*p* < 3 10^–36^) for gender and 0.355 (*p* < 2 10^–71^) for action). For both delays the extrinsic uGC, unlike the intrinsic ones, depended on the source, target power and their product to a moderate degree (Tables S4, S5). On the other hand as for the intrinsic uGC, the extrinsic ones depended mainly on high frequencies for short delays in the gender task, and on both high and low temporal frequencies for the long delays (Table S3). Not surprisingly, the uGCs with outside leads, in contrast to the intrinsic uGCs, were weak (Fig. S5-4): only for short delays in the gender task some uGCs averaged over 1.5, yielding significant task dependency only for the short delays (mixed effect, see Suppl Text 8, 9). Hence, we used only the strong (> 5) uGCs to describe the outside connections of VL and T, using the four types defined above for internal TP connections (Fig. 5D). Although ‘disappear’ connections dominated all strong connections in the gender task, they reached significantly larger proportion in Tipin (from outside to T) connections than in the other 3 anatomical classes of outside connections (Tipout = from T to outside, VLin = from outside to VL, VLout = from VL to outside; Mixed effects, see Suppl Text 10). Compared to the intrinsic strong connections, the outside ones were significantly (mixed effects, see Suppl Text 11) less frequently ‘maintain’, and more frequently ‘appear’, indicating more segregation of the two delays in extrinsic connections (compare Figs. 5D and S3-7). The NS1 component of strong outside connections displayed two important properties: synchrony with the TP activation (onset latencies of the 4 strong outside uCG were on average 2 to 13 ms earlier than those of TP activation) and clear task dependence (Figs. 5A, S5-5, S5-6).

**Figure 6:**
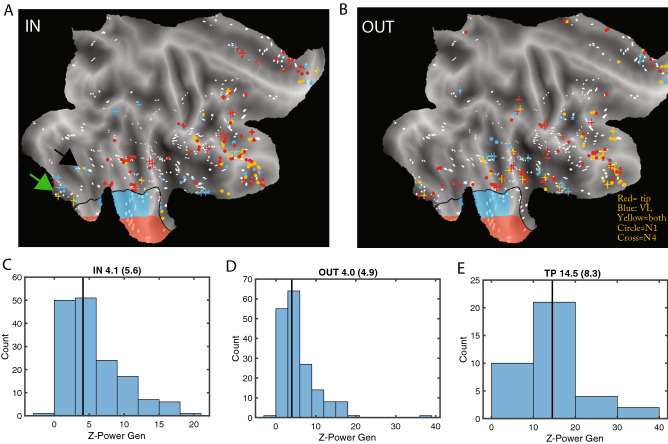
Properties of outside leads connected to T and VL: (**A**), (**B**) flatmaps of right hemisphere showing outside leads providing *input* to VL (blue), T (red) or both ( yellow) at short (dots) and long (crosses) delays (A), or receiving *output* from VL (blue), T (red) or both (yellow) at short (dots) or long (crosses) delays (B); white dots: unconnected leads; black full line border of TP with VL in blue and T in red. Black and green arrows point to FFA (black) and LG1 (green) in ventrolateral temporal cortex. (**C**), (**D**), (**E**) distribution of responses in gender task of leads providing input to Tip and VL regions of TP (**C**), receiving from T and VL (**D**) and within T and VL (**E**) with indication of the median (value in title, with interquartile range in parenthesis), which is almost three times larger in TP than outside.

Just over a quarter of outside cortical leads were positively and strongly connected with the T/VL parts of TP in either direction: median proportion inward (26%, range 16–47%), and median proportion outward (26%, range 22–40%). Interestingly, although average values were very similar in the two directions, the proportions in the two correlated little across patients (R^2^ = 0.09). This indicates that the uGCs reflect functional links and not the implantation pattern idiosyncratic of each patient. The outside leads connected with TP were located in all parts of temporal and frontal cortex, including OFC and insula (Fig. 6A, B). OFC stood out as the region most connected with TP (Tables S7, S8): median proportion inward reached 83% (range 33–100%) and median proportion outward 67% (range 33–100%), both effects being significant (mixed effect S Text 12, 13). In both directions, ventral temporal cortex (TC) was the second most connected region, and dorsolateral frontal cortex was the least connected region, all these effects being significant (mixed effects Suppl. Text 12, 13). Both the inward and outward external leads could be connected to both VL and T, or to only one of those regions. Proportionally fewer leads were connected only to VL amongst the output leads compared to input leads (Mixed effects, see Suppl Text 14), indicating that T dominates the output from TP. The temporal leads providing input to TP were segregated into dorsal and ventral regions, connected predominantly with T and VL, respectively (Fig. 6A, Tables S9, S10). Both effects of functional connectivity were significant, see mixed effects S Text 15, 16. While the ventral temporal (VT) leads connected with TP in the input direction were predominantly linked to VL, those VT leads connected in the output direction with TP were predominantly linked to T (Table S11), again a significant effect (mixed effect Suppl Text 17). Outside cortical leads negatively connected with TP showed a more restricted spatial pattern than positive leads (Fig. S6-1), but they were observed predominantly in a single patient (P1). Overall, both the positively and negatively connected leads responded in the gender task in a task dependent way, but response strength and task effect were smaller than in TP leads (Fig. 6C-E, S6-2, S6-3). Also these outside leads responded on average synchronously with the TP leads but individual leads showed large variation in onset latency (Fig. S6-4). Two VT regions providing input to VL are noteworthy: FFA in fusiform cortex (black arrow in Fig. 6A), and a region in lingual gyrus, labelled LG1 (green arrow in Fig. 6A). In agreement with^9^, the FFA leads (2 patients, Fig. S6-5B, C) responded with short latency to the static presentation in the two tasks equally. The LG1 leads (4 patients, Fig. S6-5A–D) showed a weaker and later response that was weakly task dependent. Across patients the latency of LG1 responses varied little with RT, unlike the VL leads (Fig. S6-5E, F). Remarkably, the VLin uGCs from both LG1 and FFA showed strong task dependency (Fig. S6-6A-D), and the LG1 NS1 uGCs correlated significantly (r = 0.73, *p* < 0.005) with the power of LG1 leads, unlike overall VLin NS1 uGCs (Table S5). The NS4-uGCs from LG1 were variable, ranging from absent in P1 to very strong in P3 (Fig. S6-7A-D), and differed from those in the other direction (Fig. S6-7E–H). The NS4-uGCs from FFA also differed in the two directions (Fig. S6-7F, G).

### What determines the TP gender decision responses?

**Figure 7:**
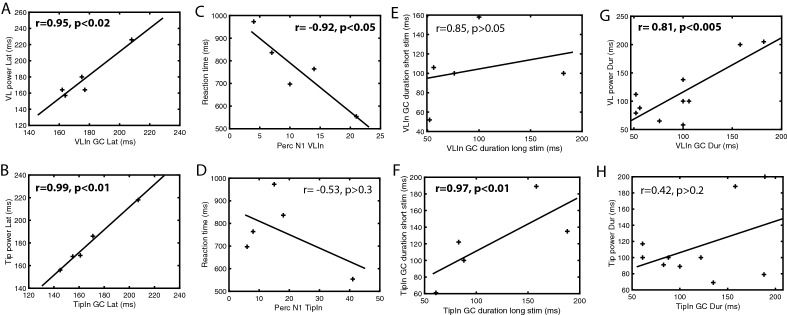
Properties of the VLin and Tipin uGCs*.* (**A**), (**B**) Latency of TP responses as a function of latency of increase in NS1 UGC, for VL/VLin (A, r = 0.95, *p* < 0.02, latVL = -78 ms + 1.45 latVLin) and T/Tipin (B, r = 0.99, *p* < 0.01, latT = 11 ms + latTipin). (**C**), (**D**) relationship between RT and percent leads with strong NS1 uGC with VL (**C**) and Tip (**D**) across patients (n = 5). For both connections the RT decreases with increasing proportions of connected leads but the correlation is significant for VLin (r = -0.92, *p* < 0.05) not Tipin (r =  − 0.53, *p* > 0.3). (**E**), (**F**) duration of increase in NS1 uGC for long stimulus presentation as function of the duration for short stimuli for VLin (**E**) and Tipin (**F**). Same as S7-3F,G. Notice that Tipin durations are very similar for both short and long stimuli, while this is not true for VLin; (**G**), (**H**) correlation of duration of TP response and duration of NS1 uGC input across patients and the two stimulus durations for VL (**G**) and for T (**H**). Correlation is significant for VL (r = 0.81, *p* < 0.005), but not for T (r = 0.42, *p* > 0.2).

While the outside leads sending signals to TP are responsive and task dependent (task dependency was significant for leads with positive or negative, inward or outward connections, mixed effects, see Suppl Text 18), they are less so than the TP leads (Fig. S6-2, S6-3). On the other hand, the strong uGCs linking these leads to TP show clear phasic, task dependent rises (Figs. 5A, S-5, S6-6). Hence the unfolding in time of TP responses seems to reflect largely the temporal properties of the functional connections, in particular the short delay uGCs. These latter were anatomically specific (Fig. 5E): both for VLin and Tipin leads the connection to Ds (3 patients) were reduced to less than 30% of those to VL or T (Fig. S6-8)) and depended little on stimulus duration (Fig. 5F). Also, the latencies of VLin NS1-uGC and of VL power correlated significantly (r = 0.95, *p* < 0.02, Fig. 7A), as did those of Tipin NS1 uGCs and T power (r = 0.99, *p* < 0.01, Fig. 7B). Further analysis confirmed the importance of the VLin NS1-uGCs for the time course of VL power. First, the proportion of Vlin (r =  − 0.92, *p* < 0.05) but not Tipin NS1-uGCs (r =  − 0.53, *p* > 0.3) correlated negatively with RTs across the five patients (Fig. 7C, D). This effect of VLin proportions implies an effect on VL response latency (which correlates with RT), and explains the difference between VL and T in the equations linking uGC and TP response latencies (Fig. 7A, B): the strong negative intercept in the VLin-VL latency equation (latency VL =  − 78 ms + 1.45 latency VLin NS1 uGC) indirectly reflects the effect of the proportion of NS1 uGCs, which decreases with latency, given a slope exceeding one. This effect of the proportion of VLin NS1-uGCs on latencies of VL responses, by the negative intercept, also sheds some light on the paradoxical observation that for patients with short RTs (patients 1, 2, 4), the VL latencies are shorter than those of the VLin leads. The second support for the role of VLin timing on TP responses comes from the effects of stimulus duration on the duration of TP responses and of NS1-uGCs increases. In agreement with^9^, when considering the five stimulus durations, the duration of VL responses hardly (< 10%) increased with stimulus duration, an effect mainly due to a single patient (Fig. S7-1). This independence of VL responses from stimulus duration was confirmed when dividing the trials into long and short halves: across patients duration of VL responses for long trials depended weakly (shallow slope and small r) on duration for short trials (Fig. S7-2). A very similar relationship (with the intercept 20 ms shorter) was observed for the VLin NS1-uGCs (Figs. 7E, S7-3), but not the Tipin NS1-uGCs (Figs. 7F, S7-3) nor Tip responses (Fig. S7-2). These Tip inputs and responses both exhibited a slope close to 1 and large r, reaching significance for Tip responses (r = 0.97, *p* < 0.01), setting them apart from the VL inputs and responses. It is noteworthy that the VLin lead responses also showed a relationship with near 1 (0.92) slope and large r (0.85, *p* > 0.05), further underscoring the differences between VL and VLin lead responses. When considering the duration of responses for short and long stimuli separately, yielding 10 data points across the 5 patients, the duration of VL responses correlated significantly (r = 0.81, *p* < 0.005) with that of VLin NS1-uGCs (Fig. 7G). This correlation, however did not hold (r = 0.42, *p* > 0.2) for the relation between T responses and Tipin NS1-uGCs (Fig. 7H), again setting Tip inputs and responses apart from VL inputs and responses. Thus not only does the VL response time course match that of the VLin NS1 uGCs, but this match between afferent connections and response sets VL apart from Tip.

### Control of the inputs to TP

**Figure 8:**
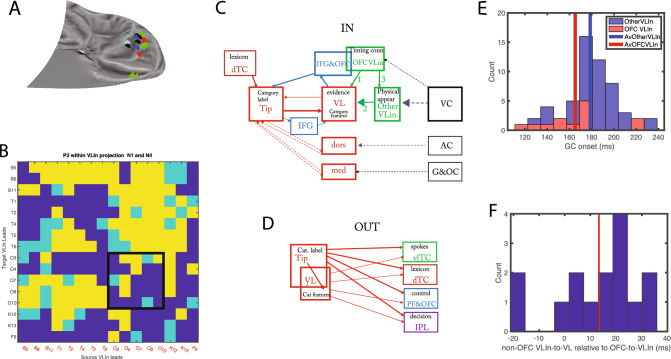
Relationship between intrinsic and outside functional connections. (**A**) location of OFC VLin leads in 5 patients (red: P1, light blue: P2, green: P3, black: P4, blue: P5) (**B**) matrix with connections between VLin leads in P3: yellow NS1 and/or NS4 uGC > 5, light blue: NS1 and/or NS4 uGC > 3 (but below 5), dark blue: both NS1 and NS4 uGC < 3; the two leads on the medial edge of OFC (O3, O4) combined are connected to all other VLin leads outside OFC (black square). (**C**) Scheme showing input functional connections to T. (**D**) Scheme with output functional connections T and VL combined; In C and D Red: TP subregions, green VLin leads, blue prefrontal and OFC, black: sensory cortices, Bordeaux: dorsal TC, purple: inferior parietal lobule; full lines: functional links inferred from present study with thickness indicating strength, stippled lines connections from literature. Black text hypothesized function. (**E**) distribution of onset latencies (mixed effect corrected) of VLin NS1 uGCs for OFC and other leads (arrows 1 and 2 in C). The two means are significantly different (mixed effects, see Suppl Text 14, *p* < 0.02). (**F**) difference between onset latencies of NS1 uGC from other non-OFC VLin leads to VL leads and from OFC VLin leads to other VLin leads (arrows 2 and 3 in C): the mean (13.3 ms) differs significantly from zero (mixed effects, see Suppl Text 15, *p* < 0.02).

If the timing of the VL responses reflects that of the short delay VLin uGCs, this calls for a region controlling the onset of these functional links. As VLin and VL leads are largely synchronous (Fig. S6-4), this control region should provide input to VL in parallel to input to Vlin leads, and thus belong to the VLin leads, in addition to being connected to the other VLin leads and being task dependent. The OFC VLin leads, located mainly in the rostral part of right OFC (Figs. 8A, S8-1C), as those responsive in the gender task (Fig. S8-1A), were indeed connected to most other VLin leads (Figs. 8B, S8-2, S8-3). In addition, the OFC leads responsive in the gender task, are known to be task dependent^9^. OFC VLin leads met two further requirements for a region controlling VLin NS1 uGCs’ timing: (1) their uGC to VL (Fig. 8C, arrow 1) had a significantly shorter onset latency (Fig. 8E, mixed effects, see Suppl Text 19) than the uGCs from the remaining VLin leads (arrow 2 in Fig. 8C), (2) the NS1 uGC from the OFC-VLin leads to the other VLin leads (Fig. 8C, arrow 3) had a shorter onset latency than their VLin NS1 uGCs (Fig. 8C, arrow 2): indeed their difference was significantly different from zero (Fig. 8F, mixed effects, see Suppl Text 20).

One final issue concerns the intrinsic VL-to-T uGCs, which were the earliest event we observed in the right TP. These functional links might arise from a small group of VL neurons that escape detection by the coarse LFPs^14^, and receive FFA input, as indicated by some FFA-VL uGCs having early onset latency (Fig. S6-6H). These ‘early’ VL neurons may then activate the OFC leads conditioning the VL-to-T connection (Fig. 8C), which overlap with the OFC-VLin leads (Fig. S8-1D). In one patient these OFC leads indeed showed face responses (Fig. S8-4), and their location is consistent with that of the lateral OFC face patch in the monkey^15^.In addition to these short delay uGCs, the ‘early’ VL neurons may also drive the long delay VL to T connections (Fig. S3-6B).

## Discussion

Our results showed that (1) the T and VL parts of right TP were most responsive in the gender task, but VL responses correlated with the RT of the gender task. These results were unaffected by the location of a substantial number of leads in the EZ. (2) The TP responses are based on visual signals from both the face and the hand of the actor. (3) The VL and T parts are linked in both directions with the VL to T flow earlier and under more external control than the stronger T to VL flow. (4) While the cortical input to TP targets mainly VL, originating predominantly in OFC and VT cortex, output arises more from T, returning to a range of outside regions, including OFC and VT cortex. (5) The time course of the TP responses reflect more the timing of the afferent links than the time course of the responses in afferent leads.

Initial studies of human temporal lobe responses to faces were investigated with event related potentials^16^, but subsequent studies used a variety of gamma band ranges: 50-150 Hz^17^, 30-70 Hz^18^, 30-100 Hz^19^, 40–90 Hz^20^. The latter authors reported FFA responses to faces with a time course very similar to the one reported here. Interestingly they failed to find a relationship between the FFA gamma band power and behavioral measures in a gender discrimination task similar to ours. We used the broad band gamma range 50–150 Hz because of its relationship with neuronal activity (see^21^). Using the broad band gamma range, the present gender task activates not only FFA but also TP^9^.

The computation of Granger causality^12,13^ between pairs of stereo-EEG leads allowed us to trace the functional connections between these recording sites. Granger causality computation yielded both strong phasic and tonic connectivity within right TP, but in the present study we concentrated on the phasic changes in an attempt to understand the remarkable properties of the phasic TP responses in the gender task^9^. Importantly, this computation allowed to trace also the direction of these transient connections and their time course, the latter being impossible with fMRI^6,7^ given the long time constant of the hemodynamic response. Indeed we were able to show that the connections between VL and T differ significantly in strength and latency in the two directions (Fig. 3). These findings are in agreement with those of single cell and electrocorticographic studies in non-human primates^22–26^. Furthermore we computed the unconditioned GC for two delays (4 ms and 16 ms), which proved to be approximately two independent measurements. Indeed there was little correlation between the uCG for the two delays in the intrinsic and outside connections of TP in either task (R^2^ ranging from 0.06 to 0.25). In addition, the conditioning leads for the VL-T connections rarely matched for the two delays. These two delays of the uGC imply functional connections operating on a very short time scale. Yet, previous intracranial evidence of multi-stage temporal processes underlying visual cognition^27,28^ demonstrated feedforward and backward dynamics with ~ 100 ms separations between activations in distant areas. This is different from the present study, in which we used Granger causality to investigate functional connectivity between two simultaneously active, neighboring TP subregions (T and VL), which, in addition, are active only very briefly. Hence it is not surprising to find 4-16 ms delays for the functional connections between these subregions, as the effects of the functional connections have to fit within the brief simultaneous activations. Furthermore our results are in line with single cell data from non-human primates. Indeed, consistent with the 8 to 13 ms delay obtained for the coupling of gamma frequencies in single cell studies of attention effects in V4 and FEF^22^, the uGCs at 16 ms delay may correspond to connections with more distant regions as shown by the location of the conditioning leads for NS4 uGCs between VL and T (Fig. 4). But this is not necessarily always so, since strong NS4-uGCs were measured between the two neighboring regions VL and T. The explanation for this apparent paradox resides in the fact that these uGCs do not necessarily indicate direct connections. In single cell studies it has been argued that frequency-resolved granger causality only applies to direct connections^29^, but it is unclear whether this generalizes to our study using time resolved granger causality applied to local field potentials. In absence of anatomical information, as was the case here, a direct connection can be inferred only if conditioning of the GC between a pair of leads is independent of the activity of all leads outside the pair, which is impossible to assess with certainty given the limits to the within-patient sampling of recording sites in this study. If the leads with conditioning effect are located in the same region as the source of the uGC, as we observed (Fig. 4), this could be considered a convergent projection from the source region, implying a direct anatomical connection between the two regions in which the leads of the conditioned pair are located.

The proportions of leads responsive in the gender task confirm that right TP is a heterogeneous structure^5–7^, with the VL region, in which more than half (58%) of the tested leads were responsive, being the entry stage for visual signals, as it occupies the most rostral part of TE. The Md subregion includes rhinal cortex which is known to host many visually responsive neurons^24^, consistent with the relatively large proportion of responsive leads (42%) observed in this subregion. Anatomical connections^5–7^ indicate that in addition to VL, Ds and some parts of Md are also unimodal subregions, linked to specific sensory systems, while T, receiving from all 3 unimodal regions^6^, is multimodal^3,30^. As semantic categories are multimodal, they should therefore be represented in T. Since the functional link VL to T is the first event in TP, it follows that category labels in T are activated by the visual evidence collected by VL in the signals carried by the afferents from the temporal VLin regions. The masking experiments showed that these VL signals arise, be it with different latency, both from the actors’ face and body, which remain segregated at lower levels^31,32^. This combination of face and body signals in human TP is consistent with our earlier prediction^33^, but difficult to compare with the face patch in monkey TP, where neurons are selective for familiar faces but not familiar bodies (^34^, Fig. S2-1). Consistent with the masking results, the temporal VLin regions included not only FFA, but also lingual regions processing color and texture information^35–38^. Hence, the visual evidence collected by VL concerns both the geometry and material properties of face and body, i.e. the physical appearance of the actor. The lingual regions processing color and texture had a longer and more variable latency than FFA leads, consistent with the increase in latency of the TP responses when masking the face. While the location of the VLin leads informs on the nature of the signals reaching VL, the time courses of VL responses reflect more those of the VLin connections than those of the VLin leads: latency, duration, strength of response and its task dependency all are more similar between VL leads and short delay VLin connections than between VL and VLin leads.

Our results also clarify the efferent projections from multimodal region T, the connectivity of which has been difficult to study with imaging^3,4^. Indeed, they indicate that the output from TP arises mainly from T or from T and VL combined, i.e. the regions representing semantic categories. Indeed T and VL are known to share many functional connections^6^. According to our results these TP *outputs* target a range of regions (Fig. 8D): (1) the VT cortex which are the sensory regions providing input to VL, i.e. the spokes of the model, consistent with^8^, and including feedback to the VLin leads (Fig. S8-3, Tables S10, S11); (2) dorsal temporal cortex i.e. possibly representing the lexicon, a path useful for naming tasks^39^; (3) the PFC and OFC control regions (although to a reduced number of leads compared to the TP input leads, Fig. S8-4, Table S7, S8) to adjust the control^40^, and (4) to inferior parietal lobule (IPL) decision regions^41^ for the semantic decisions in the present task. Although both T and VL provide output to rostral IPL (Fig. 6B), the significant correlation between response latency in VL and RT suggests that the visual evidence collected by VL is the main source of information for the semantic decision.

Jackson et al.^8^ investigated mechanisms for semantic memory by comparing a number of model architectures in terms of the degree of (1) conceptual abstraction they achieved, that is the ability to discern conceptual similarity among items denoted by images, words or other attributes, and (2) context sensitivity, in which a context-specific subset of properties of a concept needed to be generated, both of which are critical components of the task our subjects performed. They find that the best-performing model had a multimodal hub (their hidden layer 2 – HL2), that received input from multiple modality specific hubs (hidden layer 1 – HL1) that each integrate over spokes of modality-specific brain regions, some of which also sent and receive shortcut connections to the multimodal hub (these thus bypass HL1). Our findings concerning the direction of information flow within and through TP fit well with this model. Indeed, the VLin-VL-T sequence matches the optimal hub and spokes architecture^8^, with VLin leads corresponding to the spokes layer, VL to the hidden layer 1 (HL1), and T to the multimodal hidden layer 2 (HL2), with the short cuts between spokes and HL2 being instantiated by the few ventrolateral temporal leads connected directly to T (Fig. 6A). It is noteworthy that the transition from unimodal to multimodal TP regions corresponds to cytoarchitectonic distinctions^5^ between cortices with a thick layer 4 (VL) and thin layer 4 (T). Our results, however, clarify the model^8^ in several ways, specifically:

First, it proposes a solution to the problem of a small hub having to represent many categories. Our results suggest that this is solved by T (HL2) using VL (HL1) as annex for the category representation. Indeed, if many of the categories have to be localized in T, the category representation must be very reduced, limited to an abstract label, i.e. a simple index. Given the many T leads responding in a task using only very basic categories, the category label is likely distributed over large numbers of neurons, unlike what has been reported for declarative memory^42^. The automatic return activation of VL by the strong T to VL connection suggests that the abstract category label in T is strictly linked to the main visual category features in VL, possibly corresponding to the defining features of categories^10^. The link from T to the spokes (see above) may be more flexible, possibly emulating the characteristic features of semantic categories^10^. Thus T corresponds to the semantic hub in the strict sense, and its index representation solves or alleviates the problem of hosting many categories into a relatively small region, even if population codes can be computationally very efficient^43^. Such a representation may also facilitate the establishment of links between related categories. T receives input not only from VL and PFC/OFC control regions, but also from dorsal TC, which may provide input from the lexicon^44^, thereby establishing a one to one relation between abstract category labels and words. Thus categories could be activated by a different path than the physical appearance, provided by the VLin leads to VL^45^.

Second, our results also clarify the control over the TP. Indeed, while the input from ventrolateral TC provides the sensory evidence collected by VL, the timing of this input is set by VLin-OFC, which controls the VLin connections. This sets the time course of activity in the whole TP, as the direct input from outside leads to T does determine its latency but not its duration (Fig. 7). Thus, OFC, the only region outside TP hosting double selective leads in^9^, and directly connected to the TP by the uncinated fasciculus^46^, has an extensive control over the collection and transmission of sensory evidence to the T. Importantly, most OFC leads are in the most rostral part of OFC, an evolutionarily novel part, the function of which has so far remained unknown^47^. With respect to model suggestions^8^, it should be noted that the VLin-OFC controls the link between VLin leads and VL, i.e. the link spokes-HL1 in the model, rather than one of these layers, which were the optimal location for connections with the control regions according to the model^8^. In addition, the VL-T link (corresponding to the link HL1-HL2 in the model) is also under OFC control (quantified as a conditioning effect) and T itself receives input from PFC/ OFC (Fig. 6A), indicating that the link HL1-HL2 in the model is also under control of outside regions. An anatomical substrate for such outside control has recently been described for layer 6 of T^48^. Thus, our results enrich control mechanisms by indicating the presence of control at multiple levels which targets both the links between and within TP subregions (i.e. layers in the model). This is not surprising as in the modelling study^8^ the demand for control was limited, serving only to select the relevant sensory feature, while here control sets the timing of TP.

In conclusion, our study of the responses of stereo-EEG leads and their functional connections reveals the directional flow within the right TP leading to a suggestion of how the gender categories are represented by an abstract label in area TG, the multimodal part of TP, and by category features in anterior TE, its visual part. By extending the study of directional flow to the outside connections of right TP it reveals (1) the use of physical appearance as input to the TP, underscoring the importance of the senses for concrete knowledge, and (2) the control of OFC (and ventrolateral PFC) over the different processing stages feeding this input into TG, extending to functional role of OFC beyond contributing to slow cognitive control^49^. Finally our results also show that the fast processing in high level cognitive regions such as TP, is determined by the timing of the functional connections rather than that of the afferent areas, providing a mechanism for the human brain to operate on autonomous time scales. Further work is needed to extend the present results to more concrete semantic categories, to left TP, and to abstract and verbal categories.

## Methods

### Patients

Stereo Electroencephalography (stereoEEG) data were collected from 19 patients (8 female, age 21–49, mean 32 years, Tables S1 and S2) suffering from drug-resistant focal epilepsy. Fourteen of the 19 patients contributed to the previous study^9^. These patients were stereotactically implanted with intracerebral electrodes as part of their presurgical evaluation, at the Claudio Munari Centre of Epilepsy Surgery, to define the cerebral structures involved in the onset and propagation of seizure activity^50,51^. The strategy of implantation was based on the presumptive location of the epileptogenic zone (EZ), derived from clinical history, examination of non-invasive long-term video-EEG monitoring, and neuroimaging. Patients were fully informed regarding the electrode implantation and stereoEEG recordings. The present study received the approval of the Ethics Committee of Niguarda hospital (ID 939–2.12.2013) and informed consent was obtained from all patients in the study. The experimental testing of the patients was performed during the pre-surgical evaluation in accordance with the Italian and European guidelines. The two subjects shown in Fig. 1A, B and S2-1 have given their informed consent for publication of identifying information/images in an online open-access publication.

#### Inclusion criteria

As described in^9^ patient selection was based on a series of stringent anatomical, neurophysiological, neurological and neuropsychological criteria, with the specific aim of minimizing the recording of any data from pathophysiological and functionally compromised sectors of the brain tissues.

· *Anatomical criteria*: only patients whose magnetic resonance imaging (MRI) showed no anatomical abnormalities, or only very restricted anomalies were included in the study.

· *Neurophysiological criteria*: this examination includes the inspection of the EEG tracks at rest, during wakefulness and sleep from both intracranial and scalp EEG. Pathological activity is characterized by the presence of epileptic discharge at the seizure onset, but epileptic spikes may be present in leads exploring the regions surrounding the EZ during the interictal periods. Since epileptic spikes could affect the quantification of task- and stimulus-related gamma reactivity, each trial presenting an interictal epileptic discharge (IED) at any latency during the stimulus presentation was removed. Besides inspecting the spontaneous EEG activity, the neurophysiological investigation also included an assessment of the normal reactivity of both intracranial and scalp EEG to a large set of peripheral stimulations (somatosensory, visual, vestibular and auditory stimulations) to verify normal conduction times and overall sensory reactivity.

*Neurological criteria*: no seizure, no alteration in the sleep/wake cycle, and no change in pharmacological treatment must have taken place within the last 24 h before the experimental recording of a patient included in the study. Neurological examination had to be unremarkable, with in particular no motor or visual deficit.

*Neuropsychological criteria*: a series of neuropsychological tests was administered by experienced neuropsychologists. The tests focused on the evaluation of the patient skills in language (production, comprehension, reading), verbal memory, visuospatial memory, visual exploration, executive and attentional functions, visual perception, abstract reasoning. Among them, we considered of particular relevance 5 items indexing skills which could impact the ability to carry out the required tasks:

- *Semantic fluency*^52^: the overall score is followed by the ranked index, where a value greater or equal to 2 indicates normal function, a value of 1 indicates a subclinical abnormality, a value of 0 indicates a pathological dysfunction.
- *Naming*: this is a test item extracted from the Boston Naming Test^53^; a score below 20 is considered pathological.
- Visual exploration^54^; a score below 30 is considered pathological.
- Attentional matrices^52^ ; the overall score is followed by the ranked index, where a value greater or equal to 2 indicates normal function, a value of 1 indicates a subclinical abnormality, a value of 0 is considered pathological.
- *Face recognition*: Benton Facial Recognition Test^55^. A value outside the normed range (41–54) is considered pathological.

Their values, which were normal in the vast majority of patients and tests (70/78), are reported for 16/19 patients in Table S2; 3 patients were abnormal for semantic fluency, 3 also for face recognition.

#### Localization of recording sites with respect to lesions and epileptogenic zone

Only patients presenting either no anatomical alterations (n = 15) or only small abnormalities (n = 4), as evident on MRI, were included. Three of the 4 patients with positive MRI showed minimal periventricular nodular heterotopia, and one patient focal cortical dysplasia (FCD) in the frontal lobe.

The epileptogenic zone (EZ) involved generally parts of temporal or frontal cortex. Its extent was established in 18 out of the 19 patients and the overlap with the four TP parts was determined.

#### Electrode implantation

All implantations in the patients considered were made in the right hemisphere: most (16) had unilateral implantations, but 3 had bilateral implantations. A number of depth electrodes (range: 12–21) were implanted in different regions of the hemisphere using stereotactic coordinates. Each cylindrical electrode had a diameter of 0.8 mm and consisted of eight to eighteen 2-mm-long contacts (leads), spaced 1.5 mm apart (DIXI Medical, Besancon, France). Immediately after the implantation, cone-beam computed tomography was obtained with the O-arm scanner (Medtronic) and registered to preimplantation 3D T1-weighted MR images^56,57^. Subsequently, multimodal views were constructed using the 3D Slicer software package^58^, and the exact position in the brain of all leads implanted in a single patient was determined by using multiplanar reconstructions^59^. Leads were identified, following clinical conventions, by a letter corresponding to the electrode shaft, followed by a number starting from the electrode tip. The number of leads localized in the grey matter of the right hemisphere ranged from 84 to 171 (Table S1).

### Behavioral testing

Behavioral testing of the patients was exactly the same as described in^9^.

#### Setup

Patients were seated 70 cm from a liquid crystal display (Dell P2210, resolution 1680 × 1050 pixels, 60 Hz refresh rate) in a familiar environment. The visual stimuli were generated using a personal computer equipped with an open GL graphics card using the Psychophysics Toolbox extensions^60,61^ for Matlab (The Math Works, Inc.).

#### Visual stimuli and tasks

The stimuli consisted of 1.167 s videos clips showing one of two actors (male or female), standing next to a table and dragging or grasping an object (a blue or red ball) on the table using the right hand. At the start of the video, the hand could be shaped either as a palm or a fist and its position could be either above or on the table. In half of the trials, we increased the size of the video (by 20% of the original) within the aperture. The aperture was created by multiplying videos with an elliptic mask causing video clips at the borders to gradually blur into the black background. Finally, the videos were shown as recorded (actor standing to the right of the table), or mirrored around the vertical axis (actor on the left side of the table). These manipulations resulted in 64 (2^6^) videos which were then presented either in the full length (*Full trials*) or truncated either at the time point corresponding to each individual’s 84% action discrimination threshold (ranging from 200 to 250 ms) or at one of 2 other points 100 ms earlier or later (*Truncated trials*). The remainder of the movie in the truncated trials was replaced by a dynamic noise which was produced by randomly scrambling every pixel in the display on subsequent frames.

All trials started with a baseline period (1 s), followed by a variable static phase, created by repeating a first video frame, identical for the two actions, for 283, 458, 583, 733 or 883 ms, and then followed by the video displaying either action. If patients could not respond during static and dynamic stimulus presentation, they were given another 2 s to reach a decision before the trial ended. As soon as patients pressed a button during any of the 3 trial phases (static frame, video, response epoch), the inter-trial period (1 s) started. In the analyses of the effects of the duration of static presentation, trials with 283 and 458 ms static presentation were considered short trials, those 733 ms and 883 ms with static presentations as long trials.

The trials were organized into 4 blocks of action (An) or gender (Ge) discrimination tasks^9^. The order of presentation was always An-Ge-An-Ge. Each block consisted of 2 sub-blocks of 32 trials such that one sub-block contained full trials and the other truncated ones, presented in pseudorandom order. At the beginning of each sub-block, the instruction saying either ‘action’ or ‘gender’ in Italian was displayed for 5 s. The patients had to follow this instruction by performing either the action or gender two-alternative forced-choice (2AFC) discrimination task by pressing, when ready, either a right or left button with the right hand to indicate their decision. In the first 2 blocks the original videos were displayed and in the last 2, we mirrored the videos moving the actor to the opposite visual field. During the trial, a fixation cross was presented near the manipulated object in the center of the screen. During the 1 s inter-trial interval, only the fixation cross was visible.

Patients were instructed to fixate the cross in the center of the screen. In all subjects the experimenter verified that subjects complied with the fixation instruction. Eye movements were recorded in 13 patients using a noninvasive monitor-mounted infrared video system (SMI iView X 2.8.26,) sampling the positions of both eyes at 500 Hz. The measurements confirmed that patients followed the instructions to fixate well and that fixation accuracy was similar in the action and gender tasks^9^.

#### Preliminary procedures

To familiarize patients with action and actor discrimination tasks, we presented them with 2 familiarization blocks of 30 full trials chosen pseudo-randomly such that they contained equal numbers (15) of the 2 tested actions and the 2 tested actors. Patients responded by pressing a button at the end of each trial and received an auditory feedback indicating either a correct (with low pitch tone) or an incorrect (with a high pitch tone) response. The procedure was repeated until patients made fewer than 2 errors per block. Patients first learned which button corresponded to which gender and then which button corresponded to which action. In addition, every test block was preceded by a short (10 trials) familiarization block reminding patients of the proper button-choices for an upcoming discrimination task.

After completing familiarization blocks, patients viewed a block of 30 pseudorandomly chosen trials in which videos were truncated at 3 different time points (150, 250 and 350 ms) from the motion onset with the end of the video replaced by dynamic noise. After collection of the action discrimination performance, the 84%-threshold was estimated for each patient and later used in the experiment to create trials in the truncated condition.

#### Masking

In four patients, the full trials of the gender task were repeated with either the face or the hand covered by a grey rectangle 8.2° × 4.1° in size for the hand and 5.5° × 6.1° for the face (Fig. S1-2). Two blocks of 64 trials each were collected: first block with the standard video and second block with the mirrored video. Each block contained two sub-blocks of 32 trials in which 16 non masked and 16 masked trials are interleaved: first sub-block hand masked, second face masked, third face masked, fourth hand masked. Thus 32 trials with hand or face masked could be compared to 64 trials without masking.

### StereoEEG data recording

For each implanted patient, the initial recording procedure included the selection of an intracranial reference, chosen by clinicians using both anatomical and functional criteria. The reference was computed as the average signal of two adjacent leads both exploring white matter. These leads were selected patient-by-patient because they did not present any response to standard clinical stimulations, including somatosensory (median, tibial, and trigeminal nerves), visual (flash), and acoustical (click) stimulations, nor did electrical stimulation evoke any sensory and/or motor behavior^21^. The stereoEEG was recorded with a Neurofax EEG-1100 (Nihon Kohden System) at 1 kHz sampling rate.

### Standard statistical analysis

The recordings from all leads in the gray matter were filtered (band-pass: 0.08–300 Hz; notch: 50 Hz) to avoid aliasing effects and were decomposed into time–frequency plots using complex Morlet’s wavelet decomposition^9^. Power in the gamma (50 to 150 Hz) frequency band was extracted within an interval, extending from 1000 ms before the start of the trial to 1000 ms after the latest response by the subject^9^, the timing of which differs between subject and task. This interval was subdivided into non-overlapping 25 ms bins. Following previous intracranial studies^9,62^, gamma power was estimated for 10 adjacent non-overlapping 10-Hz frequency bands, and averaged. The quality of the data was visually inspected using plots of average gamma power in all trials collected for a given condition, to detect the possible presence of IEDs. All trials/leads in which any IED or other transient electrical artefacts appeared were removed.

The anatomical reconstruction procedures followed those of^21^ and included two basic steps: 1) identifying the recording leads located in the individual cortical surface using the multimodal reconstructions performed in each patient, and 2) importing these locations into a common template, using the warping of the individual cortical anatomy to the fs-LR average template.

Behavior data of the full trials were analysed as in^9^. This previous study established that the performance on the two tasks was extremely similar for the long trials. Accuracy (% correct) and reaction times (RTs) were computed for each of the 19 patients in the gender task (Table S2). Accuracies were high with a median of 98% (range 84–100%), and because of two patients with low score on face recognition, there is a correlation between accuracy and face recognition score (r = 0.72, *p* < 0.005). Reaction times were generally short (less than 1 s) with a median of 832 ms. Two patients had an RT exceeding 2 s, and those were removed from the analysis of the relationship between response latencies of leads and RTs. The remaining RTs ranged from 547 to 982 ms.

In this part of the present study only the full trials, 128 in total (64 trials for each task), were analyzed for responsiveness and specificity of the leads, in following of^9^. The analysis was performed on the average gamma band (50–150 Hz) power sampled with 25 ms bins, and z-scored against the 1 s baseline period^9^. Each trial was subdivided into three epochs: (1) the 1 s baseline epoch (BEp), ending with static stimulus onset; (2) the static epoch (SEp), defined as the 200 ms time window starting 75 ms after static onset (275 ms was duration of the shortest static phase); (3) the video epoch (VEp), which started at the end of the static presentation (variable across trials) and lasted until a patient gave a response. Responsive and specific leads were defined exactly as in^9^, using the distribution (mean and SD) of z-scores during the 1 s interval before stimulus onset as reference. Responsive leads had a z-score exceeding a threshold of 3SD during the static epoch in the gender task. Specific leads in addition did not exceed a z-score of 3SD in the static epoch of the action task (task specificity), nor in the video period of the gender task (epoch specificity). In the present study, responses were compared in different masking conditions. Since only 32 trials were sampled for each of the two masking conditions, the z-scored gamma band time courses of the responsive leads in T and VL were averaged in each patient: 6 leads in patient 1, 5 in patient 2, 3 in patient 3, and 5 in patient 7. Latency and duration of these average time courses were defined by the intersection with z-score of 2.

### Granger causality analysis

#### Patient inclusion Granger analysis

We selected 7 patients (Patients 1–7 in Table S1) with most recent recordings or large numbers of responsive leads in TP, particularly in subregions VL and T, to investigate the directional flow of information within TP and between TP and outside cortex. Since Granger causality depends on the time course of individual trials it is much more prone to artefacts than the average power in the 50–150 Hz band used in the standard analysis. Therefore we developed a specialized artefact rejection procedure (see below) which resulted in a larger fraction of rejected trials compared to our standard analysis. Therefore we included in the analysis the full trials as well as the truncated trials for both tasks, doubling the number of trials available for analysis. This is acceptable because the truncating of the videos had little effect on the behavior in the gender tasks, in which patients relied on the static part of the stimulus^9^. Indeed for the 7 patients the median accuracy was 98% in truncated trials compared to 100% in full trials. The median RT was 696 ms for truncated compared to 832 ms for full trials. Also the TP responses to the static stimulus in the truncated trials of the gender task were similar to those in the full trials^9^.

#### Preprocessing for Granger analysis

The recordings were cut in segments aligned to 1 s before static onset and lasting at least until 1 s after static onset (depending on the onset of the following trial). Since the TP response in the gender task started on average 170 ms after static onset and lasted on average less than 200 ms, the segment included all of the TP responses. For each patient there were between 81 and 171 leads located in gray matter of the right hemisphere, for which 128 trials in the “gender” and “action” task were available (256 trials total). For the initial standard analysis, which was conducted to extract the onset and task-specificity of the response and focused on the high-gamma power, artifact rejection was based on outliers in power between 50 and 150 Hz (see above). For the further analysis that involved the interaction between leads evaluated through Granger causality this was inadequate. We were interested in effects that were induced by stimulus onset and designed criteria to remove other large-amplitude activity that was not locked to onset. We identified the following artifacts: (1) large amplitude low frequency and gamma band events; (2) large-scale activity correlated between leads; (3) lead saturation and glitches where there was a coherent jump in recorded values across leads. We therefore developed a multi-pronged procedure, implemented in Matlab (the mathworks, version R2017b, R2019b, R2021b), because each criterion by itself detected only part of the artifacts we had identified by eye. We applied each criterion to the data (see below) and accordingly set the traces for specific trials and leads (or channels) to *NaN*s (not a number) so as to automatically exclude them from further analysis in the matlab routines. For each patient there were on the order of ten thousand traces to be manually inspected per task condition, which was not feasible and would also not lead to reproducible results. We therefore developed a pipeline that is characterized by a few heuristic parameters that are set separately for each patient, by evaluating the trials around the decision boundary between those that are kept and those that are discarded. This ensures reproducibility because the same setting of parameters leads to the same selection of trials/leads. The used parameters are listed in Tables 2, S12 and S13. Note that initially, we considered the action and gender trials separately in the outlier analysis (Patients 1–3, 5–6), but found that it was more efficient to combine them when the pipeline matured (Patients 4 and 7). The pipeline included the following steps.

**Table 2:** Global performance of artefact rejection for Granger Causality computation.

Patient	Task	Outlier procedure	Number Leads	Total leads-trials	Total leads-trials rejected	Fraction leads-trials rejected	Median trials per lead	Min trials per lead
1	Gender	BivarXCWav	113	14,464	1113	0.08	120	87
1	Action	BivarXCWav	113	14,464	1036	0.07	121	86
2	Gender	BivarXCWavJumpsAll	137	17,536	2932	0.17	110	19
2	Action	BivarXCWavJumpsAll	137	17,536	4146	0.24	105	12
3	Gender	BivarXCWav	125	16,000	1276	0.08	121	77
3	Action	BivarXCWav	125	16,000	1913	0.12	122	32
4	Both	BivarXCWav	145	37,120	3342	0.09	247	17
5	Gender	BivarXCWav	131	16,768	1531	0.09	118	92
5	Action	BivarXCWav	131	16,768	2241	0.13	115	45
6	Gender	BivarXCWavJumps	171	21,888	2959	0.14	114	54
6	Action	BivarXCWavJumps	171	21,888	3130	0.14	113	53
7	Both	BivarXCWavJumpsFlatness	81	20,736	6150	0.30	191	47

##### Feature-vector outliers

The data (each lead, each trial) were first detrended using the Matlab routine *detrend* in time by subtracting the best fitting linear function. The power across the entire trial epoch was determined using the routine *pwelch*, and summed across the high gamma band (50-150 Hz), this statistic was combined with the standard deviation across time into a two-component feature vector. Elements with arbitrary low values (< 1E-42) were removed before applying on the log-values the routine *mcdcov* from the LIBRA toolbox (https://wis.kuleuven.be/statdatascience/robust/LIBRA,^63^) for each lead separately. We used log-values in order to obtain a distribution that was more similar to a multivariate Gaussian. The routine first calculates robust estimates for the mean and covariance via the minimum covariance determinant method, and then outputs the corresponding Mahalanobis distance for each sample (for a given lead)^63^. For a bivariate Gaussian distribution of feature values, 97.5% of the samples lie below a Mahalanobis distance of 2.7162. For each patient, we set a percentile above which the trial-lead combinations would be rejected, this was implemented by finding samples that exceeded the corresponding Mahalanobis distance threshold. Across patients the percentile was a parameter that ranged from 90 to 99 (*BivariateRejectPercentile* in Table S12).

##### Wavelet calculation

The continuous wavelet transform (cwt) was applied using the matlab routine *cwt* with default settings on the time series at full resolution, i.e. 1 ms sampling interval. Specifically, we used the analytic Morse wavelet^64^ with the symmetry parameter (gamma) equal to 3 and the time-bandwidth product equal to 60. For each octave ten frequencies were sampled. The resulting waveforms were smooth and were down sampled by a factor 10, hence time samples were spaced 10 ms apart (matlab function *downsample*). For each lead and trial, we extracted blobs in the *frequency-scale* x *time sample* image, representing transient oscillatory events with a limited duration in a limited frequency band. Only scales corresponding to frequencies higher than 10 Hz were considered. First, we took the absolute value (the wavelet data is complex), and then binarized the image using a threshold that corresponded to the 95^th^ percentile (matlab routine *imbinarize*), after which holes, that is, lower intensity pixels inside a blob, are filled through a call to *imfill*, with option ‘*holes*’. The so called connected components (the blobs) are then individually labeled through *bwlabel*, with the parameter *connection number* equal to 8 (i.e. we consider nearest and next nearest neighbors pixels to form the connected component). Via a call to *regionprops*, we retrieve for each connected component its *Area*, *Centroid*, *BoundingBox*, *MeanIntensity*, *MaxIntensity*. For each trial/lead we keep the blob with the largest area, which are then combined into one set across trials/leads. Out of this set we select those whose frequency centroid is above a preset value (parameter: *LowerCutOffGlobalHighFreq*, Table S12) and whose maximum intensity exceeds a preset percentile of the whole set (parameter *PercentileRejectGlobalHighFreq*, Table S12). This version of the procedure is referred to as Global, as opposed to Local where we would determine percentiles for each lead, which we had initially explored as well. The corresponding trial-lead combinations so selected are removed.

##### Putative interictal events

On some trials we detected coherent events that involved a large number of leads, but that were not locked to stimulus onset. We applied the following procedure to identify these trials. For each trial, we extract the *lead vs time* matrix, z-score each lead, and divide the trial interval up in time windows of length 400 samples (sampling rate 1 kHz, i.e. the window length = 400 ms), and shift these windows with an offset = 50 samples to cover the entire trial interval. For each window and each pair of leads we determine the cross correlation with lags ranging from -150 to 150 samples. We find the maximum of the absolute value and note the corresponding delay. We keep only the maximum value (and delay) across windows. From this data we reconstitute for each trial a *lead x lead* matrix filled with the value of the maximum correlation, we refer to these as the correlation matrix. We explored a variety of ways to extract lead groups & trials with excessive correlation, and settled on the following procedure. For each trial we construct a histogram with 20 bins of the off-diagonal elements in the correlation matrix. We determine a distance between trials using the optimal transport distance^65^ between the corresponding histograms (c code at https://users.cs.duke.edu/~tomasi/software/emd.htm, ported to matlab via mex file https://nl.mathworks.com/matlabcentral/fileexchange/12936-emd-earth-movers-distance-mex-interface). Briefly, this is the cost to change one histogram into the other one by moving the mass of each density to the other across the shortest distance. The resulting distance matrix is clustered into two groups using fuzzy c-means^66^ implemented in the matlab routine *fcm*. The smaller (in terms of leads) of the two clusters represented the outliers, which we confirmed by visual inspection. All the corresponding trials were putatively tagged for removal. The removal only took place if the trials also contained above a certain threshold of bivariate outliers (see below).

##### Trial amplitude outliers

We also applied an alternate procedure to identify trials that could be considered outliers. We took the maximum of the absolute value across all leads (for each trial and time sample), yielding for each trial a time series of maxima. We then again took the maximum across time, yielding a single value for each trial. Using the matlab *isoutlier* function with default settings we extracted the outlier-trials.

##### Glitches

We noticed that on some trials all leads in one time step changed coherently up or down, we caught this by calculating the mean across all leads of the absolute value of the difference between two consecutive time samples, for each time point. Subsequently, we extracted the local maxima across time, for each trial, and determined which of them were outliers using matlab *isoutlier* function with a specified parameter 'ThresholdFactor’ (our parameter *GlitchThreshold* in Table S13). This was only relevant for three subjects and removed between 3 and 12 trials.

##### Out of range measurements

We also noticed that some signals were cut-off and replaced by flat (constant) signal during extended periods in the trial. We detect this by labeling as 1 all occurrences where the difference between consecutive samples is less than 1E-10 and count the segments of length *winlen*, shifted by *winstep* to cover the entire trial period, in which all entries are one (parameters *FlatnessWinstep* and *FlatnessWinlen* in Table S13). All trial-lead combinations where the number of such segments exceed *replen* are removed (*FlatnessRepLen* in Table S13). Only one subject (P7) displayed this effect, and 34 trial-lead combinations were removed.

The outcome of the set of two trial-based procedures (*Trial amplitude outliers, Putative interictal events*) is a union of trials slated for removal. Since removing a trial would entail removing all leads within that trial, hence severely impact the amount of data available for analysis, we added an additional criterion: a trial should contain more than a certain number bivariate outliers in order to be removed (5 to 10, *LeadThresholdForTrialBasedRejection* in Table S12). All the trials identified by ***Glitches*** were removed and combined with the set that survived the preceding criterion***.***

The outcome of *Wavelet calculation* and *Feature-vector outliers* and *Out of range measurements* is a set of trial-lead combinations obtained from the wavelet blobs, the bivariate gamma-band/standard deviation outliers and out-of-range leads. We took the union of these. Note that some trial-lead combinations were present in more than one set, so the total number trial-lead combinations removed does not match to sum of combinations in the separate sets. Likewise, the completely removed trials also contain trial-lead combinations flagged for removal. The individual number of removed trials, trial-combinations is shown in Tables S12 and S13.

The resulting total number of trial-lead combinations removed is summarized in Table 2. For relatively clean data sets less than 9% of the trial-lead combinations are removed (P1 Gender/Action, Gender for P3 and P5, Table 2. This guarantees that at least 77 trials are available for each lead, for other patients more (up to 30%) trial-leads were removed, which meant that some leads had to be discarded from analyses because too few trials were available.

#### Lead-based analysis

We performed analyses on the leads by applying a wavelet transform (*cwt*, default parameters) and averaging leads into the 50–150 Hz band. Note that we recalculated this with enhanced artifact rejection procedure rather than reusing the results of the standard analysis (see above). The wavelet analysis had as downside that time across which responses were integrated depended on the frequency band, which was different from the outcome of GC analyses, making them hard to compare. Hence, we also explored the power spectral density calculated across the same windows across which the GC was determined.

#### Unconditioned Granger causality at two delays

We first selected pairs of interest, and for each determined the set of trials which were available in both leads, in a greedy fashion. This means that for each pair potentially a different set of trials was used. We also tried a setting in which we took the trials that were present in all leads of interest (across all pairs), but this reduced the number available trials too much yielding estimates of low statistical quality.

Each time interval was divided into time-windows (length: 100 samples at 1000 Hz) that were shifted with steps of 10 samples (‘offset’) across the entire interval. We used the mvgc tool box (version 1.0^67^), which is a parametric method, but uses an efficient algorithm that in our hands was more robust than the frequency-based non-parametric method utilizing spectral decomposition^68^. For each window the Granger causality was calculated, both in the time-domain (yielding a 2 × 2 matrix for each window, of which only the off-diagonal elements are relevant) and in the frequency domain (yielding one 2 × 2 matrix for each of the 101 frequencies between 0 and 125 Hz) with lags up to 4. Lags refers to how many time steps (samples) in the past are used in the parametric model to predict the current responses. We also refer to this maximum of samples in the past as delay, for this setting the delay is 4 times 1 ms, i.e. 4 ms. This parameter setting represented the standard set, labelled NS1. We also explored two additional settings where we doubled and quadrupled the distance between samples (i.e. sampling rate of 500 Hz and 250 Hz, respectively, indicated by NS2 and NS4, respectively), this allows to extract causal influences at a longer delay. Our model order (i.e. maximum lag, corresponding to the delay in ms), was chosen based on a trade-off between temporal resolution (window length) and the number of model parameters, for which the AIC criterion in the mvgc toolbox indicated lags of 4. We indeed do find effects with a lag of 4 with sampling rate of 1000 Hz, corresponding to a delay of 4 ms, which is rather short when considering the typical 10 ms that each cortical area adds to response latency, as assessed in experiments with non-human primates using visual stimuli^69,70^. However, more recent mouse recordings using neuropixels do find spike to spike correlations, indicative of a direct synaptic connection between neurons, in different cortical regions with a median delay of 3.9 ms^71^. In the same study, the difference in response onset (first spike time) of between the visual area highest in the hierarchy and that lowest in the hierarchy is about 12 ms, indicating that our results at lag 4 with sampling rate 250 Hz, i.e. delay of 16 ms, could reflect direct interactions between further separated areas^22^, connected by long intracortical tracts, such as the uncinated tract, in addition to multi-step connections involving intermediate areas.

#### Granger causality and power in sending and receiving areas

An analytical calculation based on a first-order (lag 1) model for two leads indicates that the GC represents the logarithm of the strength of interaction and the ratio of activity in the sending over the receiving area. Hence, there is an expectation that there is correlation between GC score and power of either sending or receiving area or both. This calculation, however, does not take into account the effect of other areas on the pair of leads, nor the effects of z-scoring as well as higher model order (lag 4). We evaluated this potential correlation for both internal connections as well as external projections by determining the correlation between GC peak and power peak during the response period. In general (see Tables S4, S5) the uGC correlated weakly with the power of the source or of the target area, or the product of these powers, indicating that uGC captures the functional connection between areas without much effect of the power in the connected areas.

We performed three sets of runs, each for a different set of analyses. *First*, we took all of the selected trials and determined one time-varying Granger causality (GC) curve. *Second*, we selected 20 times a different subset of trials (with replacement, hence some trials could be missing and other trials could be used more than once), and determined the Granger causality for each. We refer to this as the bootstrap set. We then determined the mean and standard deviation across bootstraps for each time window. This gives an estimate of the statistical variability due to (limited) number of trials available, and allows us the compare Granger values for different pairs or in the same pair for different directions. *Third*, we computed the Granger causality between a lead with trials in one randomly permuted order, and the second lead of the pair with the trials in a different randomly permuted order. We repeated this calculation 20 times to obtain a mean and a standard deviation. This mean represents the GC value between leads that are not causally connected, hence it is an estimate of the bias in the GC and its variance. We use it to determine whether there is a significant causal influence of one lead on the other, by normalizing the corresponding GC score by subtracting the surrogate mean and dividing by the standard deviation. This is referred to as the score relative to surrogate data.

#### Connection strength and types of strong connections

We found that in most cases there was a significant GC during the entire trial period. Hence, to assess the task-dependent aspects we z-scored the GC scores using the mean value and standard deviation during the baseline period, which was defined as the period starting 900 ms before static stimulus onset and ending at 0 ms, at the static onset. This means that more variable GC scores that led to high standard deviation in the baseline, result in smaller z-scores. In addition, z-scores with negative sign are also of interest as they represent the case where the tasks reduces causal influences.

For each connection (both internal, between pairs of leads in T and VL as well as external between pairs with leads outside TP), we determined the maximum and minimum z-score during the response period, and combined these into a new score indicating the strength C of the response. If both minimum and maximum were positive, C was equal to the maximum, whereas if both were negative, C was equal to the minimum. When the maximum is positive and minimum is negative, and their absolute value differs less than 3, we take C as the mean of the two. If the absolute difference is larger than 3 we take as C the variable with the maximum absolute value, including its sign. We use this procedure to assign a useful signed single measure for the positive or negative strength of the response even when there is bimodal response profile in time.

These strength measures were combined across patients and calculated for two delays (sampling at 1 ms and 4 ms, NS1 and NS4, respectively) as well as the two task conditions (gender versus action). We determined the two-dimensional density of NS1 and NS4 using the matlab routine *ksdensity* with the bandwidth variable set equal to 0.8, and added level lines via matlab function *contour* for 8 levels between 0 and 0.006.

We assigned a type to external connections with the TP as well as to internal pairs (both leads within TP) based on the peak value of the GC during the response period for NS1 (sampling rate 1 kHz) and NS4 (sampling rate 250 Hz). We determined the bivariate distribution as described in the preceding section, and found that GC values exceeding a z-score of 5 constituted outliers. When the NS1 z-score exceeded 5, but the NS4 did not, we referred to those connections as disappear (D) connections, whereas if the NS4 also exceeded 5, they were ‘maintain’ (M). When the NS1 z-score was less than 5 (and not below -5), but the NS4 z-score exceeded 5, the connection was called ‘appear’ (A). When the NS4 score was below -5, and NS1 score was less than 5 (potentially also negative), we referred to the lead as ‘negative’ (N). We did not consider further the category opposite (when a z-score exceeding 5 or -5 flipped to the opposite sign when changing delay), since it was not present in our data to a sufficient level.

#### Conditioning of Granger causality

Granger causality between pairs of leads can be confounded by common input from other sources that are not included in the calculation. These influences can be exposed and accounted for by calculating the conditional Granger, which means fitting a parametric model that includes more than 2 leads, the more additional leads the better. There is however a trade-off between the window length, which needs to be short enough to follow the temporal dynamics of the tasks reflecting the engagement of causal influences between leads, and which needs to be large enough to have enough data relative to the number of parameters estimated. We found that a window length of 100 ms gave us just enough resolution to identify the brief responses. The number of parameters is the number of lags times the square of the number of leads included. It was not feasible to go beyond 3 leads, which corresponds to just one conditioning lead per pair. So as an alternative strategy we considered all remaining leads once as a conditioning lead for each pair of interest. The conditioning leads that reduced the GC z-score below 3 were considered the most influential leads because it meant that they provided the strongest common input to the pair.

#### Determination of time of response onset

We were interested in determining which leads were activated first by stimulus onset and which pairs increased the level of causal interactions the first. We therefore calculated the latency on a trial-by-trial basis. We determined the timing of power onset and peak GC scores by first finding the maximum in a region around the expected response (adjusted by patient), normalizing the traces by this maximum, determining the time sample at which 50% of peak height was first exceeded and then linearly interpolating to find where the curve would have crossed 50%. We applied this to gamma power estimates that were determined via matlab routine *pwelch*^72^ on the same set of windows that were used in the standard GC estimates (i.e. 100 samples of 1 ms) rather than the wavelet procedure introduced before so that the timing could be compared. The data were z-scored, and then bootstrapped to get 100 curves, for each of them the timing analysis was conducted and the mean determined. For GC scores we only selected those curves whose peak z-score exceeded 3. The error estimates are obtained across all available channels for power onset and across all available pairs of channels for GC (see Fig. 3I).

### Statistical tests

We combined statistics on response strength as a continuous value as well as a binary value across patients and aimed to differentiate across different variables, such as brain region and task. Following the suggestion of a recent methodological paper^73^, we used mixed effects models, where patients were a random variable (random effect) and region/task were predictors (fixed effect).

We use two types of models, models that predict continuous values such the strength of a connection and those that predict counts, either the responsiveness of a lead (binary: 0 or 1) or the count of the number of leads satisfying a particular property. In all cases, we start with a linear model that predicts a continuous value using a linear combination of the fixed effects and random effects. In some cases, where there were two distinct types of fixed effects, for instance region as well as direction of the GC score, the two effects were included as separate levels in the model. For these cases we in addition explored interaction effects, in which the product of two fixed effects was included in the formula for prediction, as long as there were enough data points relative to the number of parameters. When a continuous output is required, no additional terms are needed in the model and we fit it using the matlab routine *fitlme*, which uses maximum likelihood to find the optimal model parameters. When a binary value of count needs to be predicted we need to specify a response distribution: the binomial distribution for the binary variables and the Poisson for the counts. This distribution in addition specifies a nonlinear function, called the link function that is applied to the continuous predictor, which is the logit and log, respectively. For these models, we the matlab routine *fitglme*, in which the Laplace approximation is used to find the optimal model parameters.

Consider for example the first test in Supp. Text mixed effects.

We specify the formula as: ‘Proportion responsive = 1 + Region + (1|Patient)’.

This means that we predict whether particular leads in a particular region in a particular patient are responsive, for which the data is a sequence of zeros and ones, and corresponding region and patient labels. The model is comprised of an overall mean, indicated by the first ‘1’, and a fixed effect of the region, ‘Region’ in the equation. Note that if there are four regions, there are only three fixed effect levels, for which we use in the mathematical formulation indicator values (1 if the lead came from a particular region, zero otherwise). Three indicator values are then enough, because if they are all zero, the lead came from the fourth region. Hence, four indicator values would be dependent (their sum always equal to 1), and the algorithm would not converge. The patient is a random effect and it contributes a shift in mean, indicated by the last ‘1’.

We provide in addition the line: ‘(Binomial, 176, 4 fixed eff. coeff. = region, 19 random eff. coeff. = patient)’.

The first entry is the response distribution, Binomial, because we model binary data, followed by the number of observations, the number of fixed effects (4, for which we obtain only 3 values), and the number and identity of random effects.

The fixed effects are then quantified in the table in terms of the intercept (the coefficient of first ‘1’ in the model), and the names of the three fixed effect regions, for which we give the t value (with the effective number of degrees of freedom, i.e. the number observations minus fixed effect parameters) and the p value. We report a fixed effect as significant when the level value is significant relative to background (overall mean).

We also visualized the mixed effect models, for instance in Fig. 1E: we represented for each lead in one of the four TP anatomical regions (T, VL, Md, Ds) the responsiveness as a binary value. A mixed effect model was then determined as above with the patient considered as a random effect and the brain region as a fixed effect. The results are displayed using a violin plot (downloaded from https://github.com/bastibe/Violinplot-Matlab, written by Bastian Bechtold), based on the fraction responsive leads for each patient and each region (displayed as dots), with the horizontal lines representing the predicted mean responsiveness across patients for each region obtained from the model fit (using the matlab *fitted* function).

### Visualization of lead location

We used a set of surface files (i.e. fsaverage.R.cartesian-std.164k_fs_LR.coord) downloaded from the caret legacy site (http://brainvis.wustl.edu/sumsdb/archive_index.html), exported to and rendered in matlab using user-written programs. We used both the flatmap as well as inflated surface representations^74^.

## Supplementary Information

Supplementary Information 1.

Supplementary Information 2.

## Data Availability

Data and code will be made available upon reasonable request to PT, subject to preserving patient confidentiality.

## Abbreviations

2AFC: Two alternative forced choice
A: Appear connection
Ac: Action
AIC: Aikake information criterion, used in the mvgc toolbox to determine optimal lag of GC calculation
BEp: Baseline epoch
C: Signed strength of the TP response combining negative and positive peaks
Cen S: Central sulcus
Cing S: Cingulate sulcus
D: Disappear connection
Ds: Dorsal subregion of TP
EEG: Electro encephalogram
EZ: Epileptic zone
FCD: Focal cortical dysplagia
FFA: Fusiform face area
fMRI: Functional Magnetic resonance imaging
GC: Granger Causality
Ge: Gender
HL1: Hidden layer 1
HL2: Hidden layer 2
Hz: Hertz
IED: Interictal epileptic discharge
IFG: Inferior frontal gyrus
IFS: Inferior frontal sulcus
IPS: Intraparietal sulcus
IPL: Inferior parietal lobule
ITS: Inferior temporal sulcus
Lat: Latency
LG: Lingual gyrus
LG1: Functional region on the LG
LR: Left right
M: Maintain connection
Md: Medial subregion of TP
MR: Magnetic resonance
MRI: Magnetic resonance imaging
ms: Millisecond
N: Negative connection
NS1: Number of samples 1, GC scores based on a sampling interval of 1 ms
NS4: Number of samples 4, GC scores based on a sampling interval of 4 ms
OFC: Orbito-frontal cortex
OTS: Occipito-temporal sulcus
PFC: Prefrontal cortex
RT: Reaction time
SD: Standard deviation
SEp: Static epoch
STS: Superior temporal sulcus
T: Tip subregion of TP
TA: Cyto-architectonic region of temporal lobe
TE: Cyto-architectonic region of temporal lobe
TG: Cyto-architectonic region of temporal lobe
Tipin: Projections from cortex outside TP to Tip
Tipout: Projections from T to cortex outside TP
TP: Temporal pole
uGC: Unconditioned Granger Causality
VEp: Video epoch
VL: Ventrolateral subregion of TP
VLin: Projections from cortex outside TP to VL
VLout: Projections from VL to cortex outside TP
VT: Ventral temporal
Y: Year
