# Supplementary material for: Uncovering the fast, directional signal flow through the human temporal pole during semantic processing
Source: Sci Rep. 2023 Apr 26;13:6831. doi: 10.1038/s41598-023-33318-5 (PMC10133264; doi:10.1038/s41598-023-33318-5)
Supplement: Supplementary file 1 — Supplementary Information 1. [file 41598_2023_33318_MOESM1_ESM.docx]

Supplementary material.

Supplementary text: mixed effects statistical tests

1 *Proportion responsive leads in 4 TP parts* (Tip, VL, Md, Ds) estimated in 19 patients.

Proportion responsive= 1+Region+ (1 | Patient) (Binomial, n=176, 4 fixed eff. coeff. = region, 19 random eff. coeff. = patient)

| Name effect | Tval (df 172) | pval |
| --- | --- | --- |
| intercept | -0.77 | 0.44 |
| Region Tip | 5.20 | **5.7 x10^-7^** |
| Region VL | -0.76 | 0.45 |
| Region Ds | -4.21 | **5.8x10^-5^** |

Proportion of responsive leads in Tip (large) and Dorsal (small) is significant.

2 *Proportion of selective leads in four TP parts* (Tip, VL, Md, Ds) estimated in 19 patients

Proportion selective= 1+ region+ (1 | patient) (binomial, n=176, 4 fix eff coeff, 19 rand eff coeff)

| Name effect | Tval (df 172) | pval |
| --- | --- | --- |
| intercept | -2.67 | 0.009 |
| Region Tip | 3.83 | **0.0002** |
| Region VL | 1.53 | 0.13 |
| Region Md | -0.25 | 0.80 |

Although different reference similar result large proportion in Tip and small proportion in DS are significant.

3 Univariate analysis of *Strength of 10 internal NS1 and NS4 connections in two tasks* (difference from zero) in 7 patients

Strength=1 + (1 | patient) (n varies, 1 fix eff coeff, 7 rand eff coeff)

3A: NS1-Gender

| Connection | tVal (df) | pVal (unc, corr for 10 comp significant in bold) |
| --- | --- | --- |
| Within Tip Fw | 1.80 (46) | 0.08 |
| Within Tip Bw | 2.50 (46) | *0.016* |
| VL-T Fw | 2.63 (66) | *0.011* |
| VL-T Bw | 3.07 (66) | **0.0031** |
| Within VL Fw | -0.94 (35) | 0.35 |
| Within VL Bw | 2.37 (35) | *0.023* |
| VL-Ds Fw | -0.19 (37) | 0.85 |
| VL-Ds Bw | 0.73 (37) | 0.47 |
| Tip-Ds Fw | 2.83 (41) | *0.007* |
| Tip-DS Bw | 1.55 (41) | 0.13 |

3B: NS4-Gender

| Connection | tVal | pVal (unc, corr for 10 comp significant in bold) |
| --- | --- | --- |
| Within Tip Fw | 2.48 (46) | *0.017* |
| Within Tip Bw | 2.75 (46) | *0.008* |
| VL-T Fw | 2.68 (66) | *0.009* |
| VL-T Bw | 4.17 (66) | **0.0001** |
| Within VL Fw | 1.37 (35) | 0.18 |
| Within VL Bw | 1.72 (35) | 0.095 |
| VL-Ds Fw | -0.07 (37) | 0.95 |
| VL-Ds Bw | 0.98 (37) | 0.33 |
| Tip-Ds Fw | -040 (41) | 0.68 |
| Tip-DS Bw | 0.70 (41) | 0.49 |

3C: NS1-Action

| Connection | tVal | pVal (unc, corr for 10 comp significant in bold) |
| --- | --- | --- |
| Within Tip Fw | 1.33 (46) | 0.19 |
| Within Tip Bw | 1.58 (46) | 0.12 |
| VL-T Fw | 1.94 (66) | 0.057 |
| VL-T Bw | 2.15 (66) | *0.035* |
| Within VL Fw | -0.83 (35) | 0.41 |
| Within VL Bw | 1.64 (35) | 0.11 |
| VL-Ds Fw | 0.31 (37) | 0.76 |
| VL-Ds Bw | 1.06 (37) | 0.29 |
| Tip-Ds Fw | 3.89 (41) | **0.0005** |
| Tip-DS Bw | -0.06 (41) | 0.95 |

3D: NS4-Action

| Connection | tVal | pVal (unc, corr for 10 comp significant in bold) |
| --- | --- | --- |
| Within Tip Fw | -0.56 (46) | 0.58 |
| Within Tip Bw | 0.68 (46) | 0.50 |
| VL-T Fw | -0.21 (66) | 0.83 |
| VL-T Bw | 4.87 (66) | **0.00001** |
| Within VL Fw | -1.96 (35) | 0.058 |
| Within VL Bw | 0.94 (35) | 0.35 |
| VL-Ds Fw | -1.59 (37) | 0.12 |
| VL-Ds Bw | 2.80 (37) | *0.008* |
| Tip-Ds Fw | -0.06 (41) | 0.95 |
| Tip-DS Bw | 0.26 (41) | 0.80 |

4 *Comparisons two tasks for NS1 and NS4 uGC strength* for internal connections in 7 patients

4A Comparison Gender Action for NS1 internal connections in 7 patients

uGC strength = 1 + Task + (1 | Patient) (Binomial, n=920, 2 fixed eff. coeff, 7 random eff coeff)

| Name effect | T value (df=918) | pValue |
| --- | --- | --- |
| intercept | 3.27 | 0.0015 |
| gender | 9.45 | **10^-15^** |

4B Comparison Gender Action for NS4 internal connections in 7 patients

uGC strength = 1 + Task + (1 | Patient) (Binomial, n=920, 2 fixed eff. coeff, 7 random eff coeff)

| Name effect | T value (df 918) | P Value |
| --- | --- | --- |
| intercept | 2.14 | 0.035 |
| gender | 8.34 | **10^-15^** |

Internal connections are significantly stronger in gender than action for both NS1 and NS4.

5 *Number of four connection types ( disapp, maint, appear, negative) in 5 internal TP connections* (within Tip, T-VL, within VL, VL-Ds, Tip-Ds, both directions averaged) in 7 patients

Number= 1+ anatomical connection+ connection types + (1 | patient) (Poisson, n=96, 8 fix eff coeff, 7 rand eff coeff)

| Name effect | T value ( df 88) | P Value |
| --- | --- | --- |
| intercept | 0.37 | 0.71 |
| Within Tip | 1.95 | 0.06 |
| VL-Tip | 5.89 | **10^-7^** |
| Within VL | -2.89 | **0.005** |
| VL-Ds | -1.56 | 0.12 |
| Disappear | 5.07 | **10^-5^** |
| Maintain | 2.37 | **0.02** |
| Appear | -2.06 | **0.045** |

No significant interactions.

Number of all 3 connection types is significant (large for Dis and Maint, small for App), as is that of VL-Tip (large) and within VL (small);

6 *Number of four connection types (disapp, maint, appear, negative) in two directions of 3 internal connections* (within T, VL-T, within VL) in 7 patients.

Number= 1 + Direction + Connection type+ (1 | Patient) (Poisson, n=56, 5 fixed eff coeff, 7 rand eff coeff)

| Name effect coefficient | T value (df 51) | P Value |
| --- | --- | --- |
| intercept | 0.48 | 0.63 |
| forward | -5.04 | **10^-5^** |
| disappear | 5.72 | **10^-6^** |
| maintain | 4.28 | **10^-4^** |
| appear | -2.31 | **0.025** |

Count of all 3 connection types (large for Disapp & Maint, small for App) is significant as is the small number in forward direction.

7 *Region of conditioning leads (source TP, other TP, outside TP) for two directions of T-VL NS1 connection* in 5 patients

Number= 1 + Region of Origin+ Direction + Region of Origin x Direction+ (1 | Patient) (Poisson, n=24, 6 fix eff coeff, 5 rand eff coeff)

| Name effect coefficent | T value (df 18) | P value |
| --- | --- | --- |
| intercept | 1.40 | 0.18 |
| TP source | -1.55 | 0.14 |
| Other TP | -3.05 | **0.01** |
| T to VL connection | -2.63 | **0.02** |
| TP source x T to VL | 2.30 | **0.04** |
| Other TP x T to VL | 0.53 | 0.60 |

The small number of conditioning leads in other TP regions is significant, as is the small number of conditioned leads in the T to VL direction; the larger number of conditioning from the TP source for the T to VL direction is also significant.

7bis Region of conditioning leads (source TP, other TP, outside TP) for two directions of T-VL NS4 connection in 5 patients

Number= 1 + Region of Origin+ Direction + Region of Origin x Direction+ (1 | Patient) (Poisson, n=24, 6 fix eff coeff, 5 rand eff coeff)

| Name effect coefficent | T value (df 18) | P value |
| --- | --- | --- |
| intercept | 2.12 | 0.05 |
| TP source | -3.08 | **0.01** |
| Other TP | -1.17 | 0.26 |
| T to VL connection | 0.28 | 0.78 |
| TP source x T to VL | 1.84 | 0.08 |
| Other TP x T to VL | -1.08 | 0.29 |

Only small number of leads in TP source is significant, no significant effect of direction, no interaction.

8 *Strength of four external connections (with Tip and VL in both directions*) for two delays and two tasks in 5 patients

Strength=1 + (1 | patient) ( n varies, 1 fix eff coeff, 5 rand eff coeff)

8A Gender NS1

| connection | T Value (df ) | P Value ( only corrected) |
| --- | --- | --- |
| VLin | 23.1 (1980) | **10^-100^** |
| VLout | 1.47 (1980) | 0.14 |
| Tipin | 5.79 (2440) | **10^-8^** |
| Tipout | 3.62 (2440) | **0.0003** |

8B Gender NS4

| connection | T Value (df ) | P Value ( only corrected) |
| --- | --- | --- |
| VLin | 0.40 (1980) | 0.69 |
| VLout | 1.14 (1980) | 0.25 |
| Tipin | 0.26 (2441) | 0.80 |
| Tipout | 2.04 (2441) | 0.04 |

8C Action NS1

| connection | T Value (df ) | P Value ( only corrected) |
| --- | --- | --- |
| VLin | 2.09 (1980) | 0.04 |
| VLout | 1.11 (1980) | 0.27 |
| Tipin | 1.13 (2440) | 0.26 |
| Tipout | 1.90 (2440) | 0.06 |

8D Action NS4

| connection | T Value (df ) | P Value (only corrected) |
| --- | --- | --- |
| VLin | 4.76 (1980) | **10^-5^** |
| VLout | 1.60 (1980) | 0.11 |
| Tipin | 1.16 (2441) | 0.24 |
| Tipout | 2.53 (2441) | **0.011** |

Although connection are weak they are significant mainly for NS1 in gender and for NS4 in action

9 *Comparison strength external connection for two tasks* in 5 patients for two delays

Strength= 1+Task + (1 | patient)

9A Task dependency of external connections for NS1 (n=17688, 2 fix eff coeff, 5 rand eff coeff)

| Name effect | T value (df 17686) | P Value |
| --- | --- | --- |
| intercept | 4.56 | **10^-5^** |
| gender | 27.43 | **10^-120^** |

9B Task dependency of external connections for NS4 (n=17692, 2 fix eff. coeff, 5 rand eff coeff)

| Name effect | T value (df 17690) | P Value |
| --- | --- | --- |
| intercept | 1.55 | 0.12 |
| gender | 0.96 | 0.34 |

External connection are task dependent for NS1 but not NS4

10 *Number of 4 connection types amongst four outside connections* in 5 patients

Number= 1 + connection types+ anatomical connections+ conn typ X anat conn + (1 | patient)

(Poisson, n=80, 16 fix eff coeff (7 main, 9 interactions), 5 rand eff coeff)

| Name effect | T value (df 64) | P Value |
| --- | --- | --- |
| intercept | 9.51 | 10^-13^ |
| VLin | -1.28 | 0.20 |
| VLout | -1.65 | 0.10 |
| Tipin | -0.44 | 0.66 |
| Disappear | 13.54 | **10^-19^** |
| Maintain | -5.77 | **10^-6^** |
| Appear | 2.41 | **0.02** |
| Disapp x VLin | -0.12 | 0.90 |
| Disapp x VLout | -1.73 | 0.09 |
| Disapp x Tipin | 2.29 | **0.03** |
| Maint x VLin | -0.60 | 0.55 |
| Maint x VLout | -0.90 | 0.37 |
| Maint x VLout | -1.84 | 0.07 |
| Appear x VLin | -1.63 | 0.11 |
| Appear x VLout | 1.79 | 0.08 |
| Appear x Tipin | -0.95 | 0.35 |

The number of the 3 connection types is significant (large for disappear and appear, low for maintain as is the large number of disappear amongst tipin connections

11 *Number of four connection types in intrinsic and external connections compared* in 7 patients

Number= 1 + Conn type + Location+ conn type x loc + (1 | Patient) (Poisson, n=136, 8 fix eff coeff ( 5 main, 3 interactions, 7 rand eff coeff)

| Name effect | T value (df 128) | P value |
| --- | --- | --- |
| intercept | 3.54 | 0.001 |
| external | 6.74 | **10^-9^** |
| disappear | 10.94 | **10^-19^** |
| maintain | 0.32 | 0.75 |
| appear | -1.45 | 0.15 |
| Ext x disappear | 0.54 | 0.59 |
| Ext x Maintain | -6.72 | **10^-9^** |
| Ext x Appear | 2.88 | **0.005** |

Overall the large number of Disappear is significant, as is the small number of maintain and the large number of appear amongst the external leads

*12 Number of connected leads providing input to TP per anatomical region* in 5 patients

Number of leads= 1+ regionID + (1|patient) (binomial, n=38, 8 fixed eff coeff, 5 random eff coeff)

| Fixed effect | T Value (df=30) | P value |
| --- | --- | --- |
| Intercept | -4.50 | 9.46 10^-5^ |
| Region ID DT | -0.81 | 0.42 |
| Region ID VT | 2.26 | **0.03** |
| Region ID Par | -3.07 | **0.0046** |
| Region ID Med Front | 1.35 | 0.19 |
| Region ID DL Front | -2.37 | **0.025** |
| Region ID VL Front | 1.86 | 0.07 |
| Region ID OFC | 5.69 | **3.31 10^-6^** |

*13 Number of connected leads receiving Output from TP per anatomical region* in 5 patients

Number of leads= 1+ regionID + (1|patient) (binomial, n=38, 8 fixed eff coeff, 5 random eff coeff)

| Fixed effect | T Value (df=30) | P value |
| --- | --- | --- |
| Intercept | -4.41 | 0.00001 |
| Region ID DT | 0.31 | 0.75 |
| Region ID VT | 2.20 | **0.035** |
| Region ID Par | 0.05 | 0.95 |
| Region ID Med Front | 0.11 | 0.92 |
| Region ID DL Front | -3.25 | **0.003** |
| Region ID VL Front | -2.02 | 0.052 |
| Region ID OFC | 5.00 | **2.31 10^-5^** |

14 *Identity of TP regions (only VL, only Tip, and VL/TIP combined) and direction (IN, OUT) in external connections* in 5 patients

Number= 1 + TP region + Direction+ region x direction + (1 | patient) (Poisson, n=30, 6 fix eff coeff, ( 4 main, 2 interactions), 5 rand eff coeff)

| Name effect | T value (df=24) | P value |
| --- | --- | --- |
| intercept | 36.83 | 10^-20^ |
| Only VL | -6.01 | **10^-5^** |
| Combined VL,T | 1.27 | 0.22 |
| IN | 0.25 | 0.81 |
| IN x Only VL | 3.24 | **0.004** |
| IN x combined VL,T | -2.13 | **0.05** |

Although the small number of Only VL connections is significant, these Only VL connections occur significantly more amongst inputs, which in turn target significantly less VL and T combined.

*15 Number Dorsal Temporal cortex leads providing inputs to only VL, only T, or to both* in 5 patients

Number of leads= 1 + region ID + (1|patient) (Binomial, 15 observations, 3 fixed effects coeff, 5 random eff coeff)

| Fixed effect | T value (df 12) | P Value |
| --- | --- | --- |
| Intercept | -2.28 | 0.04 |
| Only VL | -1.02 | 0.33 |
| Only Tip | 3.26 | **0.007** |

Inputs from Dorsal Temporal cortex go significantly more frequently to T only

*16 Number Ventral Temporal cortex leads providing inputs to only VL, only T, or to both* in 5 patients

Number of leads= 1 + region ID + (1|patient) (Binomial, 12 observations, 3 fixed effects coeff, 5 random eff coeff)

| Fixed effect | T value (df 9) | P Value |
| --- | --- | --- |
| Intercept | -2.46 | 0.04 |
| Only VL | 3.89 | **0.004** |
| Only Tip | -2;05 | 0.07 |

Inputs from ventral cortex go significantly more frequently to VL only

*17 Number Ventral Temporal cortex leads receiving outputs from only VL, only T, or from both* in 5 patients

Number of leads= 1 + region ID + (1|patient) (Binomial, 15 observations, 3 fixed effects coeff, 5 random eff coeff)

| Fixed effect | T value (df 12) | P Value |
| --- | --- | --- |
| Intercept | -1.53 | 0.15 |
| Only VL | -2.06 | 0.06 |
| Only Tip | 3.70 | **0.003** |

Ventral Temporal cortex leads receive significantly more frequently outputs from only T.

18 *Task dependency of power of outside cortical leads with IN and OUT connections,* in 5 patients for positive and negative connections with TP

Power= 1 + task+ (1 | patient)

18A: Positive IN (n=420, 2 fix eff coeff, 5 rand eff coeff)

| Name effect | T value (df 418) | P value |
| --- | --- | --- |
| intercept | 9.63 | 10^-19^ |
| gender | 12.28 | **10^-28^** |

18B Negative IN (n=82, 2 fix eff coeff, 5 rand eff coeff)

| Name effect | T value (df 80) | P value |
| --- | --- | --- |
| intercept | 6.19 | 10^-7^ |
| gender | 3.89 | **0.0005** |

18C Positive OUT (n=488, 2 fix eff coeff, 5 rand eff coeff)

| Name effect | T value (df 486) | P value |
| --- | --- | --- |
| intercept | 6.36 | 10^-9^ |
| gender | 11.39 | **10^-25^** |

18D Negative OUT (n=46, 2 fix eff coeff, 5 rand eff coeff)

| Name effect | T value (df 44) | P value |
| --- | --- | --- |
| intercept | 6.90 | 10^-7^ |
| gender | 3.25 | **0.003** |

Significant task effect in all four groups of external connected leads

19 *Comparison of latency OFC VLin NS1 uGC and other VLin NS1 uGC* in 5 patients

Latency uGC= 1 + Type VLin + (1 | patient) (n=67, 2 fix eff coeff, 5 rand eff coeff)

| Name effect | T value (df 65) | P value |
| --- | --- | --- |
| intercept | 17.79 | 10^-25^ |
| OFC-VLin | -2.53 | **0.01** |

The latency of OFC-VLin uGC is significantly earlier (by 9 ms on average) than that of other VLin uGC

20 *Difference between latency OFC VLin to Other Vlin NS1 uGC and Other VLin NS1 uGC compared to zero* in 5 patients.

Difference in uGC latency = 1 + (1 | Patient) (n=15, 1 fix eff coeff, 5 rand eff coeff)

Intercept: T value (df 14) = 2.93, p value= **0.01**

Thus the difference in latency is significantly different from zero

**Supplementary tables**.

Supplementary Table 1: Patients

| Patient  ID | gender | age | MR | Post Surg | Nb Hem leads | NbTP leads | Mas  king | Granger  Intr stren | Granger Intr tim &Outside |
| --- | --- | --- | --- | --- | --- | --- | --- | --- | --- |
| 1 | F | 21 | FCD | n.a; | 114 | 9 | + | + | + |
| 2 | F | 39 | NEG | n.a. | 146 | 6 | + | + | + |
| 3 | M | 39 | NEG | n.a. | 128 | 13 | + | + | + |
| *4* | F | 32 | NEG | FCD Ia | 148 | 14 |  | + | + |
| *5* | M | 35 | NEG | negative | 141 | 16 |  | + | + |
| *6* | F | 23 | NEG | n.a. | 171 | 13 |  | + |  |
| 7 | F | 32 | NEG | FCD IIb | 85 | 10 | + | + |  |
| *8* | M | 45 | NEG | n.a. | 140 | 15 |  |  |  |
| *9* | M | 30 | PNH | n.a. | 123 | 11 |  |  |  |
| *10* | M | 22 | NEG | n.a. | 115 | 8 |  |  |  |
| *11* | F | 30 | PNH | n.a. | 85 | 8 |  |  |  |
| *12* | M | 33 | NEG | negative | 84 | 5 |  |  |  |
| *13* | F | 49 | PNH | negative | 103 | 8 |  |  |  |
| *14* | M | 34 | NEG | n.a. | 136 | 2 |  |  |  |
| *15* | M | 21 | NEG | negative | 126 | 8 |  |  |  |
| *16* | F | 27 | NEG | negative | 140 | 11 |  |  |  |
| *17* | M | 25 | NEG | TBD | 127 | 10 |  |  |  |
| *18* | M | 42 | NEG | n.a. | 96 | 6 |  |  |  |
| 19 | M | 38 | NEG | FCD Ia | 105 | 3 |  |  |  |
|  |  |  |  |  |  |  |  |  |  |

Italic indicate patients common to previous study (Ref. 9). The last three columns indicate the analyses that were performed on the dataset for the corresponding patient in this paper.

Nb: number; Intr: intrinsic, stren: strength, tim: timing

Supplementary Table 2: Psychophysical performance and neuropsychology tests

| Patient | Accuracy  (% correct) | RT | Semantic  Fluency | Naming | Visual exploration | Executive  Function  (Att mat) | Face  recognition |
| --- | --- | --- | --- | --- | --- | --- | --- |
| 1 | 98% | 547ms | **15/0** | **18** | 33 | 58/4 | 52 |
| 2 | 91% | 832ms | n.a. | 23 | 30 | 48/2 | **34** |
| 3 | 100% | 928ms | 30/2 | 23 | 30 | 46/1 | 47 |
| 4 | 100% | 707ms | 55/4 | 24 | 31 | 55/2 | ***39*** |
| 5 | 100% | 982ms | 57/4 | 24 | 34 | 56/3 | 47 |
| 6 | 100% | 663ms | ***29/0*** | 20 | 35 | 57/3 | 52 |
| 7 | 100% | 2245ms | 38/2 | 24 | 34 | 56/3 | 49 |
| 8 | 97% | 824ms | 51/4 | 24 | 35 | 58/3 | 49 |
| 9 | 94% | 916ms | 48/4 | 24 | 35 | n.a. | 49 |
| 10 | 98% | 799ms | n.a. | n.a. | n.a. | n.a. | n.a. |
| 11 | 100% | 562ms | 42/3 | 23 | 34 | 57/3 | 50 |
| 12 | 97% | 859ms | n.a. | n.a. | n.a. | n.a. | n.a. |
| 13 | 100% | 827ms | 34/1 | 24 | 34 | 59/4 | 54 |
| 14 | 95% | 923ms | 30/1 | 22 | 32 | 52/2 | 45 |
| 15 | 93% | 866ms | ***29/0*** | 23 | ***22*** | 52/2 | 52 |
| 16 | 100% | 767ms | ***28/0*** | 24 | 33 | 60/4 | 45 |
| 17 | 95% | 2446ms | n.a. | n.a. | n.a. | n.a. | n.a. |
| 18 | 84% | 753ms | 53/4 | 24 | 30 | 44/1 | ***24*** |
| 19 | 98% | 755ms | 48/4 | 24 | 33 | 55/2 | 47 |
| Median | 98% | 827ms |  |  |  |  |  |

Because of the two patients with lowest score on face recognition, there is a correlation between accuracy and face recognition score (FRS, r=0.72, p<0.005; % correct= 77+0.4 FRS)

Supplementary Table 3: Correlation Time dependent uGC with high (55-120Hz) and low frequencies (1-10Hz)

| Label | PearsonHighFreq | PearsonLowFreq | pHighFreq (log_10_) | pLowFreq (log_10_) |
| --- | --- | --- | --- | --- |
| GenderN1: Int | 0.84 | 0.39 | -237.09 | -33.28 |
| GenderN1: LatIn_N1 | 0.80 | 0.19 | -132.08 | -5.51 |
| GenderN1: LatOut_N1 | 0.79 | 0.31 | -124.95 | -14.36 |
| GenderN1: TipIn_N1 | 0.82 | 0.01 | -147.84 | -0.12 |
| GenderN1: TipOut_N1 | 0.82 | 0.14 | -146.32 | -3.35 |
| GenderN4: Int | 0.59 | 0.74 | -84.08 | -153.33 |
| GenderN4: LatIn_N4 | 0.69 | 0.52 | -83.87 | -41.71 |
| GenderN4: LatOut_N4 | 0.61 | 0.61 | -61.22 | -61.71 |
| GenderN4: TipIn_N4 | 0.54 | 0.70 | -45.71 | -87.17 |
| GenderN4: TipOut_N4 | 0.66 | 0.73 | -75.14 | -100.41 |
|  |  |  |  |  |
| ActionN1: Int | 0.67 | 0.43 | -113.30 | -40.55 |
| ActionN1: LatIn_N1 | 0.57 | 0.58 | -51.97 | -53.01 |
| ActionN1: LatOut_N1 | 0.49 | 0.77 | -36.19 | -118.56 |
| ActionN1: TipIn_N1 | 0.58 | 0.56 | -55.11 | -50.71 |
| ActionN1: TipOut_N1 | 0.51 | 0.68 | -39.64 | -81.72 |
| ActionN4: Int | 0.52 | 0.66 | -60.12 | -108.49 |
| ActionN4: LatIn_N4 | 0.50 | 0.56 | -38.17 | -50.32 |
| ActionN4: LatOut_N4 | 0.33 | 0.78 | -15.68 | -124.66 |
| ActionN4: TipIn_N4 | 0.41 | 0.74 | -24.84 | -102.01 |
| ActionN4: TipOut_N4 | 0.32 | 0.79 | -14.81 | -127.47 |

Supplementary Table 4: correlation uGC and power: NS1

| Connection | CorrWithSender | pVal Sender | CorrWithTarget | pVal Target | CorrWithProduct | pVal Product | Npts |
| --- | --- | --- | --- | --- | --- | --- | --- |
| BW:TipToVL | 0.11 | 0.39 | -0.16 | 0.65 | -0.01 | 0.03 | 62 |
| FW:VLToTip | 0.03 | 0.10 | 0.53 | 4.95 | 0.12 | 0.46 | 62 |
| TipOut | 0.14 | 11.70 | 0.13 | 11.13 | 0.11 | 7.49 | 2583 |
| VLOut | 0.09 | 4.70 | 0.13 | 9.05 | 0.13 | 8.43 | 2122 |
| TipIn | 0.07 | 3.38 | 0.07 | 3.56 | 0.07 | 3.81 | 2583 |
| VLIn | 0.21 | 21.83 | 0.04 | 1.16 | 0.16 | 12.46 | 2122 |

Supplementary Table 5: correlation uGC and power NS4

| Connection | CorrWith Sender | pValWith Sender | CorrWith Target | pValWith Target | CorrWith Product | pValWith Product | Npts |
| --- | --- | --- | --- | --- | --- | --- | --- |
| BW:TipToVL | 0.18 | 0.81 | -0.26 | 1.37 | -0.15 | 0.62 | 62 |
| FW:VLToTip | -0.26 | 1.40 | -0.04 | 0.14 | -0.20 | 0.91 | 62 |
| TipOut | 0.19 | 22.41 | 0.09 | 5.13 | 0.16 | 15.27 | 2583 |
| VLOut | 0.08 | 3.53 | 0.02 | 0.47 | 0.06 | 2.30 | 2122 |
| TipIn | -0.02 | 0.68 | 0.18 | 19.53 | 0.06 | 2.88 | 2583 |
| VLIn | 0.12 | 7.66 | -0.07 | 2.59 | 0.06 | 2.46 | 2122 |

Supplementary Table 6: coverage of parts Right hemisphere by leads outside TP. Rows show patients 1-5 and total

| Occipit | DorsTemp | VentTemp | Pariet | MedFront | DLFront | VLFront | OFC | Insula | Total |
| --- | --- | --- | --- | --- | --- | --- | --- | --- | --- |
| 0 | 17 | 5 | 6 | 13 | 2 | 40 | 6 | 16 | 105 |
| 0 | 24 | 3 | 10 | 27 | 16 | 43 | 6 | 11 | 140 |
| 0 | 31 | 5 | 0 | 18 | 14 | 29 | 9 | 9 | 115 |
| 33 | 52 | 15 | 19 | 3 | 0 | 2 | 6 | 4 | 134 |
| 0 | 30 | 16 | 15 | 13 | 8 | 28 | 6 | 9 | 125 |
| 33 | 152 | 44 | 50 | 74 | 40 | 142 | 33 | 48 | 619 |

The separation between dorsal and ventral temporal is in the occipito-temporal sulcus; that between dorsolateral (DL) and ventrolateral (VL) frontal cortex is in the middle of middle frontal gyrus and central gyrus

Supplementary Table 7: proportion of connected lead: input to TP

|  | DT | VT | Par | MedFron | DLFron | VLFron | OFC | Insula | Total |
| --- | --- | --- | --- | --- | --- | --- | --- | --- | --- |
| Patient 1 | 24 | 60 | 0 | 54 | 0 | 63 | 83 | 25 | 47 |
| Pt 2 | 8 | 0 | 20 | 26 | 0 | 29 | 33 | 18 | 19 |
| Pt 3 | 32 | 80 | - | 33 | 29 | 31 | 78 | 11 | 37 |
| Pt 4 | 15 | 33 | 5 | 0 | - | 100 | 83 | 0 | 16 |
| Pt5 | 35 | 37 | 0 | 38 | 0 | 14 | 100 | 22 | 26 |
| median | 24 | 37 | 3 | 33 | 0 | 31 | 83 | 18 | 26% |

VT: ventral temporal cortex; DT: dorsal Temporal cortex

Supplementary Table 8: proportion of connected leads: output from TP

|  | DT | VT | Par | MdFron | DLFron | VLFron | OFC | Insula | Total |
| --- | --- | --- | --- | --- | --- | --- | --- | --- | --- |
| Patient 1 | 6 | 40 | 0 | 23 | 0 | 27 | 83 | 19 | 26 |
| Pt 2 | 46 | 67 | 90 | 44 | 0 | 21 | 33 | 100 | 40 |
| Pt 3 | 29 | 33 | - | 21 | 7 | 17 | 67 | 33 | 24 |
| Pt 4 | 25 | 33 | 26 | 0 | - | 0 | 67 | 50 | 22 |
| Pt 5 | 33 | 44 | 0 | 31 | 0 | 14 | 100 | 33 | 27 |
| Median | 29 | 40 | 13 | 23 | 0 | 17 | 67 | 33 | 26% |

Supplementary Table 9: numbers of leads in DT (total 29) sending input only to VL, only to T or to both.

|  | Pt 1 | Pt 2 | Pt 3 | Pt 4 | Pt 5 | Total |
| --- | --- | --- | --- | --- | --- | --- |
| Only VL | 2 | 0 | 1 | 4 | 0 | 7 (24%) |
| Both | 0 | 0 | 4 | 2 | 0 | 6 (21%) |
| Only Tip | 2 | 2 | 5 | 2 | 5 | 16 (55%) |

Supplementary Table 10: number of leads in VT (total 18) sending input only to VL, only Tip or both.

|  | Pt 1 | Pt2 | Pt 3 | Pt 4 | Pt 5 | Total |
| --- | --- | --- | --- | --- | --- | --- |
| Only VL | 3 | 0 | 2 | 4 | 4 | 13 (72%) |
| Both | 0 | 0 | 0 | 1 | 2 | 3 (17%) |
| Only Tip | 0 | 0 | 2 | 0 | 0 | 2 (11%) |

Supplementary Table 11: number leads in VT (total 18) receiving output only from VL, only Tip, or both.

|  | Pt 1 | Pt 2 | Pt 3 | Pt 4 | Pt 5 | Total |
| --- | --- | --- | --- | --- | --- | --- |
| Only VL | 1 | 0 | 0 | 0 | 2 | 3 (17%) |
| Comb | 1 | 0 | 0 | 0 | 1 | 2 (11%) |
| Only Tip | 0 | 2 | 2 | 5 | 4 | 13 (72%) |

Supplementary table 12: Methods Artefact rejection

| Patient Number | Task | Outlier Procedure | Bivariate Reject Percentile | Trials Indicated by XCFCM | Trials Indicated by MaxAbs | Percentile Reject Global HighFreq | Lower CutOff Global HighFreq | Lead  Threshold  For Trial  Based  Rejection |
| --- | --- | --- | --- | --- | --- | --- | --- | --- |
| 1 | Gender | BivarXCWav | 97.5 | 18 | 7 | 85 | 15 | 5 |
| 1 | Action | BivarXCWav | 95 | 4 | 3 | 85 | 15 | 5 |
| 2 | Gender | BivarXCWavJumpsAll | 98.5 | 39 | 0 | 90 | 15 | 5 |
| 2 | Action | BivarXCWavJumpsAll | 97.5 | 54 | 6 | 90 | 10 | 5 |
| 3 | Gender | BivarXCWav | 98 | 39 | 1 | 95 | 15 | 5 |
| 3 | Action | BivarXCWav | 98 | 45 | 0 | 90 | 10 | 5 |
| 4 | Both | BivarXCWav | 99 | 39 | 11 | 93 | 10 | 10 |
| 5 | Gender | BivarXCWav | 97.5 | 13 | 9 | 90 | 15 | 5 |
| 5 | Action | BivarXCWav | 97.5 | 36 | 9 | 95 | 10 | 5 |
| 6 | Gender | BivarXCWavJumps | 98 | 8 | 0 | 90 | 15 |  |
| 6 | Action | BivarXCWavJumps | 97.5 | 10 | 0 | 90 | 15 |  |
| 7 | Both | BivarXCWavJumpsFlatness | 90 | 20 | 43 | 75 | 10 |  |

Supplementary table 13: Methods: Artefact rejection

| Patient Number | Task | Outlier Procedure | Trials Glitch Removed | Glitch Threshold | Trials Leads FlatnessRemoved | Flatness  Winstep | Flatness  Winlen | FlatnessRepLen |
| --- | --- | --- | --- | --- | --- | --- | --- | --- |
| 1 | Gender | BivarXCWav |  |  |  |  |  |  |
| 1 | Action | BivarXCWav |  |  |  |  |  |  |
| 2 | Gender | BivarXCWavJumpsAll | 12 | 15 |  |  |  |  |
| 2 | Action | BivarXCWavJumpsAll | 11 | 15 |  |  |  |  |
| 3 | Gender | BivarXCWav |  |  |  |  |  |  |
| 3 | Action | BivarXCWav |  |  |  |  |  |  |
| 4 | Both | BivarXCWav |  | 20 |  |  |  |  |
| 5 | Gender | BivarXCWav |  |  |  |  |  |  |
| 5 | Action | BivarXCWav |  |  |  |  |  |  |
| 6 | Gender | BivarXCWavJumps | 5 | 15 |  |  |  |  |
| 6 | Action | BivarXCWavJumps | 5 | 15 |  |  |  |  |
| 7 | Both | BivarXCWavJumpsFlatness | 3 | 30 | 34 | 5 | 10 | 5 |

Legends S Figures

**Figure** S1-1: **A**: examples of the 3 types of leads: specific, responsive and unresponsive. The mean (over trials) power in the 50-150Hz band, z-scored by the 1s interval preceding static onset, is plotted as a function of time relative to static onset. **B, D**: proportion of responsive leads (of tested leads) across parts of TP (B) and across the 19 patients (D). **C, E**: proportion of selective leads (of tested leads) across the four parts of TP (C) and the 19 patients (E). Same conventions as fig 1.

**Figure** S1-2: **A-D**: average power (average of z-scored power of individual leads) as a function of time after static onset in the gender (brown) and action (green) task in the four TP parts; Hatching indicates 1SE. RTs averaged 740 ms after static onset in gender task and 1400 ms after video onset in action task; Notice the strong suppression in VL and T during the video epoch in the action task.

**Figure** S1-3: Control for the EZ: **A**: Proportion of leads in the EZ in the four TP parts; **B**: proportion of responsive leads in and outside the EZ; **C**: Response strength of T and VL responsive leads in and outside EZ.

**Figure** S2-1: **A**: static frame of videos without mask, with face masked and hand masked; **B,C**: Average responses (time course of average of z-scored power of individual leads) T (B) and VL (C) leads (3 each) of patient P1 (same as fig 2) to static onset in the gender task without mask, with face masked and with hand masked. Hatching is SE.

**Figure** S3-1: (below diagonal) Raw unconditioned GC between leads in Tip (blue I1-I3) in VL (green I7-9) and dorsal (red T5-7) parts of TP in patient P1 for the gender task in the forward (towards the rostral end, green) and backward direction (away from the rostral end, purple); (above diagonal) we show the unconditioned GC normalized by subtracting the mean and dividing by the standard deviation of the surrogate (which are obtained by shuffling the trials of the leads of the pair, see Methods). The dashed dotted line, when visible, indicates z=3, if it is not visible it means that y-scale of the figure starts above z=3. On the diagonal responses of the leads to static onset in gender task on the same timescale (0= static onset). Notice the anatomical specificity of the tonic uGC for pairs involving dorsal part. The dotted black vertical at 150 ms indicates the approximate onset of response as a guide to the eye.

**Figure** S3-2. Frequency resolved (0-125 Hz) raw unconditioned GC between leads in tip (blue), VL (green) and dorsal (red) parts of TP in patient P1 for gender task in the forward direction (from column lead to row lead, shown below diagonal) and backward direction (above diagonal). Same data as in fig S3-1. Red square: panels with strong broadband gamma phasic increase; orange square: panels with weak broadband gamma phasic increase; black square: panels with tonic broadband gamma increase. The horizontal black dotted line is 50 Hz, whereas the vertical line indicates the approximate onset at 150 ms after onset of static stimulus.

**Figure** S3-3. Z-scored (on the pre-stimulus period) uGC between leads in tip (blue), in VL (green) and dorsal (red) parts of TP in patient P1 in gender task (below diagonal) and action task (above the diagonal) in forward (purple) and backward direction (green). The hatching indicate +/- 1STD obtained by bootstrapping. On the diagonal responses of the leads to static onset in gender (red) and action (blue) task on the same time interval. In all panels stippled horizontal lines indicate z-score=3. Same data as in fig S3.1.

**Figure** S3-4: Time course NS1 and NS4 uGC within TP (P5): **A**: VL to T; **B**: T to VL. Time courses at different delays : 4ms (NS1), 8 ms (NS2) and 16ms (NS4) and for different window lengths and offsets as indicated in the inset: NS indicates the sampling interval, WL the window length in ms, OFF is the shift in ms for moving the window over the trial interval. Both NS1 and NS4 uGCs are stronger in the backward direction.

**Figure** S3-5: Distributions of NS1 and NS4 intrinsic uGCs. **A, B**: distribution of NS1 uGC in gender (A) and action (B) tasks. **C, D**: distribution of NS4 uGC in gender (C) and action (D) tasks. **E**: NS4 uGC as a function of NS1 uGC in gender (red) and action (blue) task; Correlations are small, but significant: 0.496 (p=10^-55^) for gender and 0.287 (p=10^-18^) for action. In A-D red vertical lines indicate z-score uGC = ± 5.

**Figure** S3-6: Strength of the ten types of connections (mixed effects corrected) between and within TP subparts. **A, B**: strength of NS1 uGC for gender (A) and action (B) tasks; **C, D:** strength of NS4 uGCs for gender (C) and action (D) tasks; **E**: mean strength for the two tasks and two delays. In A-D: green: backward, purple: forward connections. Vertical lines are SE across patients (n=7) in A-D and across connections (n=10) in E; asterisks: significant difference from zero (mixed effects, see mixed effects test 3) at p<0.05 (one) or p<0.01 (two).

**Figure** S3-7: Distribution of the four strong functional connection types for the five connections inside and between TP parts (averaged over the two directions). Notice dominance of disappear and maintain types (= strong NS1 uGCs) within tip and between tip and VL.

**Figure** S3-8. As in Figure 3H, data for all five patients. From left to right, z-scored power averaged across leads, the uGC from tip to VL averaged across pairs, and from VL to tip, respectively, for, from top to bottom, patient 1 to 5. The shading represent the standard error across leads or pairs. For each patient we state the number of pairs, and how many of the forward/backwards, respectively, are strong: P1: 9 uGCs; 6/3 strong; P2: 5 uGCs; 2/4 strong; P3: 5 uGCs; 0/3 strong; P4: 18 uGCs; 0/15 strong; P5: 25 uGCs; 4/22 strong.

**Figure** S4-1: Conditioning of the within Tip backward connection. **A, B**: number of effective leads in origin (Tip), other TP regions, and outside TP conditioning the NS1 (A) and NS4 (B) uGCs. **C**: flatmap of right hemisphere showing the location of the via (blue, green) and non-via (red, black) leads outside TP conditioning NS1 (red, blue) and NS4 (black, green) uGCs. Notice the only via-lead conditioning NS1 uGCs located in OFC.

**Figure** S5-1: Lateral (A) and ventral (B) views of inflated right hemisphere showing all tested leads outside TP in 5 patients. Color code: red: P1, light blue: P2, green: P3, black: P4 blue: P5.

**Figure** S5-2: Time course of VLin uGCs at different delays (4ms, 8ms, 16ms) in five patients: P1 (A), P2 (B), P3 (C) P4 (D) and P5 (E). Same color convention as fig. 3C and S3-4.

**Figure** S5-3: Distribution of extrinsic NS1 and NS4 uGCs. **A, B**: distribution of NS1 (A) and NS4 (B) uGC in gender task. **C, D**: distribution of NS1 (C) and NS4 (D) uGC in action task. **E, F**: joint distributions for gender (E) and action (F) tasks. G: Correlation between NS1 and NS4 uGCs in gender (red) and action (blue) tasks. Correlations are small: r= 0.254 (p<3 10^-36^) for gender and 0.355 (p< 2 10^-71^) for action. Red lines in A to F indicate a z-score of ± 5; in G these are indicated in blue.

**Figure** S5-4: **A**: Strength (mixed effects corrected) of NS1 and NS4 uGCs for the four outside connections (to and from VL, to and from T) in gender (left of middle) and action (right of middle) task; **B**: strength (mixed effect corrected) of NS1 and NS4 UGCs in two tasks. In A asterisks indicate significantly different from zero (mixed effects, see mixed effects test 8 ): p<0.05 (one) and p<0.01 (two). In B vertical lines indicate SE across connections (n=4).

**Figure** S5-5: **A-C**: Time courses of NS1 uGCs to and from VL (A), to and from Tip (B) and between VL and T (C) in patient P5 performing gender (red) or action (blue) tasks. **D**: mean onset latencies of power in VL and T, of the NS1 uGC between VL and T, of NS1 uGCs for the four outside connections; hatching in A and B are STD, for C it is SE and horizontal lines in D indicate SE; Notice that the four outside connections start generally synchronously with power in TP (mean difference across 5 patients range from -12 to -2ms for the four outside connections).

**Figure** S5-6: Extension of Fig 5A to all patients. From left to right, patient 1 to 5, (top) z-scored power, followed by, continuing from the top, uGC from external leads to VL, and from VL, from external leads to tip, and from tip. The shading represents one 1STD,

**Figure** S6-1: **A, B**: Flatmaps of right hemisphere showing the localization of outside leads connected negatively towards TP (A) and away from TP (B). Color code indicates strength of uGC (in relative terms: OUT between 0.07 and 1.74; IN between 0.05 and 3.14). The leads are concentrated in PFC, insula and STG but this mainly reflects the fact that most of these leads were observed in patient P1 (see S5-1). Notice that negative input is much stronger than output in PFC.

**Figure** S6-2: **A-F**: Distributions of response (z-scored) of leads with strong *positive* connections to T/VL (A,B), strong connections away from T/VL (C,D) and T/VL leads (E,F) in gender (A,C, E) and action (B,D,F) tasks; Mean (SD) are indicated in the text above the distributions.

**Figure** S6-3: **A-D**: Distributions of response (z-scored) of leads with strong *negative* connections to T/VL (A,B), strong connections away from T/VL (C,D) in gender (A,C) and action (B,D) tasks. It is noteworthy that the very responsive leads in A were all located in OFC.

**Figure** S6-4: Timing of onset of outside connected leads relative to T/VL onset**. A**: distributions of onset latencies of leads with strong connection to T/VL relative to earliest T/VL lead; **B**: distribution of onset latencies of leads with strong connection away from T/VL relative to latest T/VL onset. **C, D**: distributions of T/VL lead onsets as used in A and B respectively. As indicated the means of the distributions in A and B differ much less than one SD from zero.

**Figure** S6-5: **A-D**: Time course of power of VL (blue), T (red), FFA (green) and LG1( black) leads in gender (full lines) and action task (stippled lines) in four patients: P1 (A), P3 (B), P4 (C), and P5 (D); **E,F**: relationship in 4 patients between latency and RT for LG1 (E) and VL (F). Notice shallower slope in LG1.

**Figure** S6-6: Time courses of NS1 uGCs connecting LG1 and FFA with TP. **A-D**: time courses of VLin NS1 uGCs from LG1 in patient P1 (A) and P5 (B) and from FFA in patient P4 (C) and P5 (D) in gender (red) and action (blue) tasks. Error band indicates SD. **E**: Time course of Tipin NS1 uGC from LG1 in gender (red) and action (blue) tasks in patient P1, for comparison with A; **F,G**: time course of uGCs connecting LG1 to VL (F) and VL to LG1 (G) in patient P5: NS1,NS2,NS4 corresponding to delays of 4, 8 and 16ms. Same conventions as fig 3C and S3-4; **H**: Time course of individual VLin uGCs from FFA in Patient P5: 15 curves indicate connections from 3 FFA leads (red, green, black) to 5 VL leads. Notice the spread of onset latencies.

**Figure** S6-7: **A-H**: Average time courses during gender task of uGC from LG1 to VL (A-D), and from VL to LG1 (feedback, E-H) at 4ms delay (NS1), 8ms delay (NS2), and 16ms delay (NS4) in patient P1 (A, E), P3 (B, F), P4 (C, G), and P5 (D, H). Same conventions as fig 3C and S3-4.

**Figure** S6-8: **A-C**: Average time courses of NS1 uGCs from VLin leads to all VL leads (blue) and single dorsal leads (red, yellow, purple) in patients P1 (A), P3 (B) and P5 (C); **D-F**: Average time courses of NS1 uGCs from Tipin leads to all Tip leads (blue) and single dorsal leads (red, yellow, purple) in patients P1 (D), P3 (E) and P5 (F).

**Figure** S7-1: Duration VL responses for 5 stimulus durations. **A-E**: Time power in the 0.1 to 0.4ms window after onset for five stimulus durations (color code) in patient P1 (A), P2 (B), P3 (C), P4 (D) and P5 (E). **F**: median (n=5) duration TP response (at half of maximum change from baseline) as a function of stimulus duration; the correlation is significant (r=0.94, p<0.02) but the slope very shallow (0.07). The slopes for individual patients are: –0.03 (A), 0.04 (B), 0.06 (C), 0.02 (D), and 0.12 (E).

**Figure** S7-2: Duration of TP responses for short trials and long trials. **A-E**: Time course z-scored power of tip (left panels) and VL (right panels) in the 0.1 to 0.4 ms window after onset for short (blue) and long (red) trials in patient P1 (A), P2 (B), P3 (C), P4 (D) and P5 (E); **F,G**: duration VL(F) and Tip (G) response (at one third of maximum change from baseline) long trials as a function of that for short trials (F same as fig 6). Notice the outlier in F is same as in S7.1 E.

**Figure** S7-3: Duration VLin and Tipin NS1 uGCs for short and long trials. **A-E**: Time course z-scored VLin ( blue, yellow) and Tipin (red, purple) uGCs and VL in the 0.0 to 0.5 ms window after onset for short (blue, red) and long (yellow, purple) trials in patient P1 (A), P2 (B), P3 (C), P4 (D) and P5 (E); **F, G**: duration VLin (F) and Tipin (G) increase for long trials as a function of that for short trials (same as fig 7). In A-E horizontal lines indicate z-score=2 at which the durations were measured. Notice slope close to 1 in G, not F.

**Figure** S8-1: OFC leads. **A**: Responsive leads in 19 patients, **B:** tested leads in 5 patients in which outside TP connection studied. **C**: OFC VL in leads in 5 patients; **D**: VL to T conditioning leads in 2 patients. In A red and blue dots: responsive and unresponsive leads, respectively; In B-D: color code patients: red: P1, light blue: P2, green: P3, black: P4, and blue: P5.

**Figure** S8-2: Matrix of uGCs from all outside leads (VLin leads in red font at the end) to VLin leads in patient P3: Yellow uGC>5, light blue >3, dark blue<3. Same patient as fig 8; in this patient O3, 4,7,9, 10,11,12,13,14 and Y 14,15 are OFC leads, but only a subset (O3,4,7,9,10) are VLin. Black (OFC -VLin) and red (other VLin) rectangles indicate connections within OFC, which are reduced.

**Figure** S8-3: Matrix of uGCs from all outside leads (VLin leads at the end) to VLin leads in patient P1: Yellow uGC>5, light blue >3, dark blue<3. Leads O6-11 are OFC leads, but only a subset (O8, 9, 10) are VLin (black rectangle).

**Figure** S8-4: **A, B**: Average responses of OFC leads positively (A) and negatively linked to VL (3 each) of patient P1 (same as fig 2, S2-1) to static onset in the gender task without mask, with face masked, and hand masked

**Figure** S8-5: **A, B**: Detail of right hemisphere flatmaps showing leads in prefrontal cortex with connections to T/VL (A) and from T/VL (B); same data and same conventions as in fig 6A,B. CS: central sulcus.
