# Supplementary material for: Uncovering the fast, directional signal flow through the human temporal pole during semantic processing
Source: Sci Rep. 2023 Apr 26;13:6831. doi: 10.1038/s41598-023-33318-5 (PMC10133264; doi:10.1038/s41598-023-33318-5)

Figure S1-1

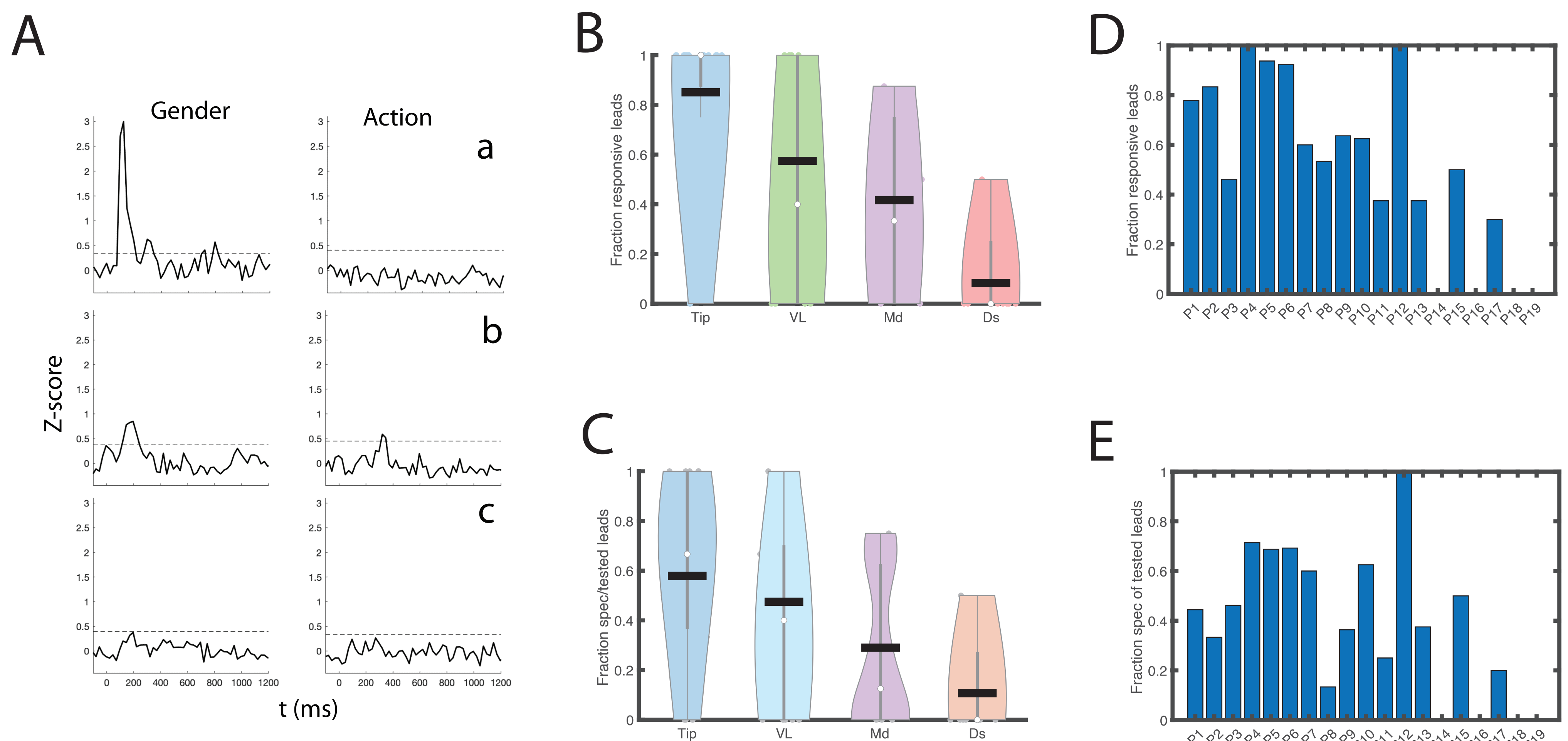
A
Gender
Action
a
b
c
Z-score
t (ms)
B
Fraction responsive leads
Tip
VL
Md
Ds
C
Fraction spec/tested leads
D
Fraction responsive leads
E
Fraction spec of tested leads
P1 P2 P3 P4 P5 P6 P7 P8 P9 P10 P11 P12 P13 P14 P15 P16 P17 P18 P19

Figure S1-2

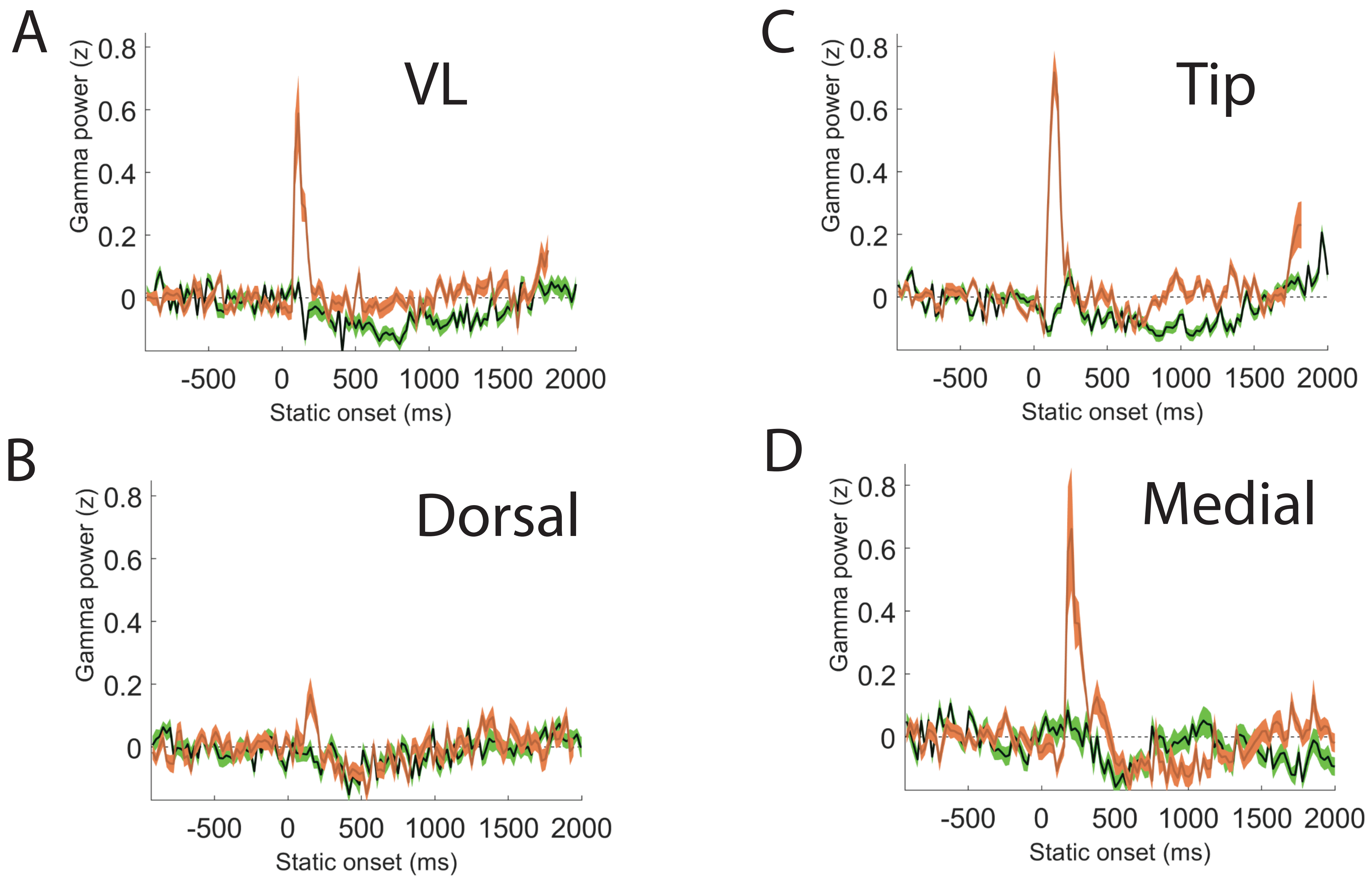
A
VL
Gamma power (z)
0.8
0.6
0.4
0.2
0
-500
0
500
1000
1500
2000
Static onset (ms)
B
Dorsal
C
Tip
D
Medial

Figure S1-3

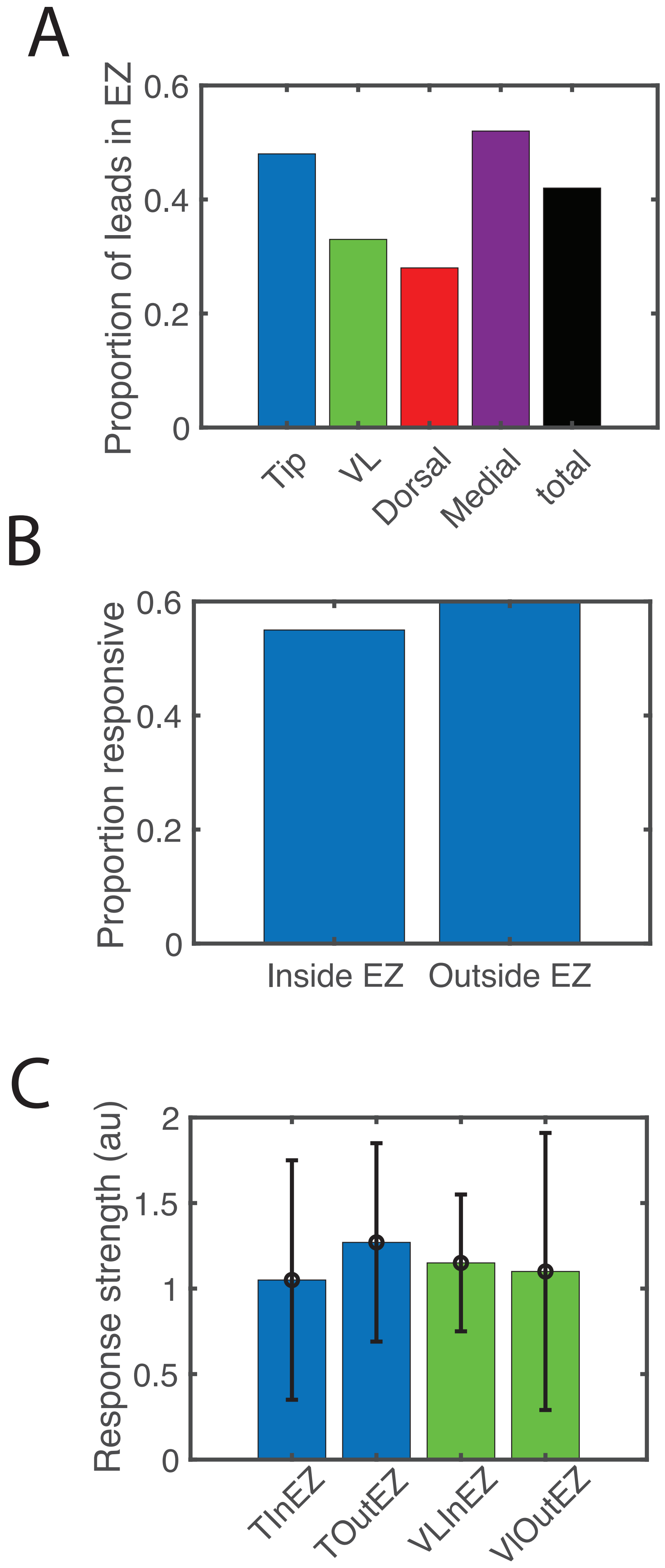
A
Proportion of leads in EZ
0.6
0.4
0.2
0
Tip
VL
Dorsal
Medial
total
B
Proportion responsive
0.6
0.4
0.2
0
Inside EZ
Outside EZ
C
Response strength (au)
2
1.5
1
0.5
0
TInEZ
TOutEZ
VLInEZ
VlOutEZ

Figure S2-1

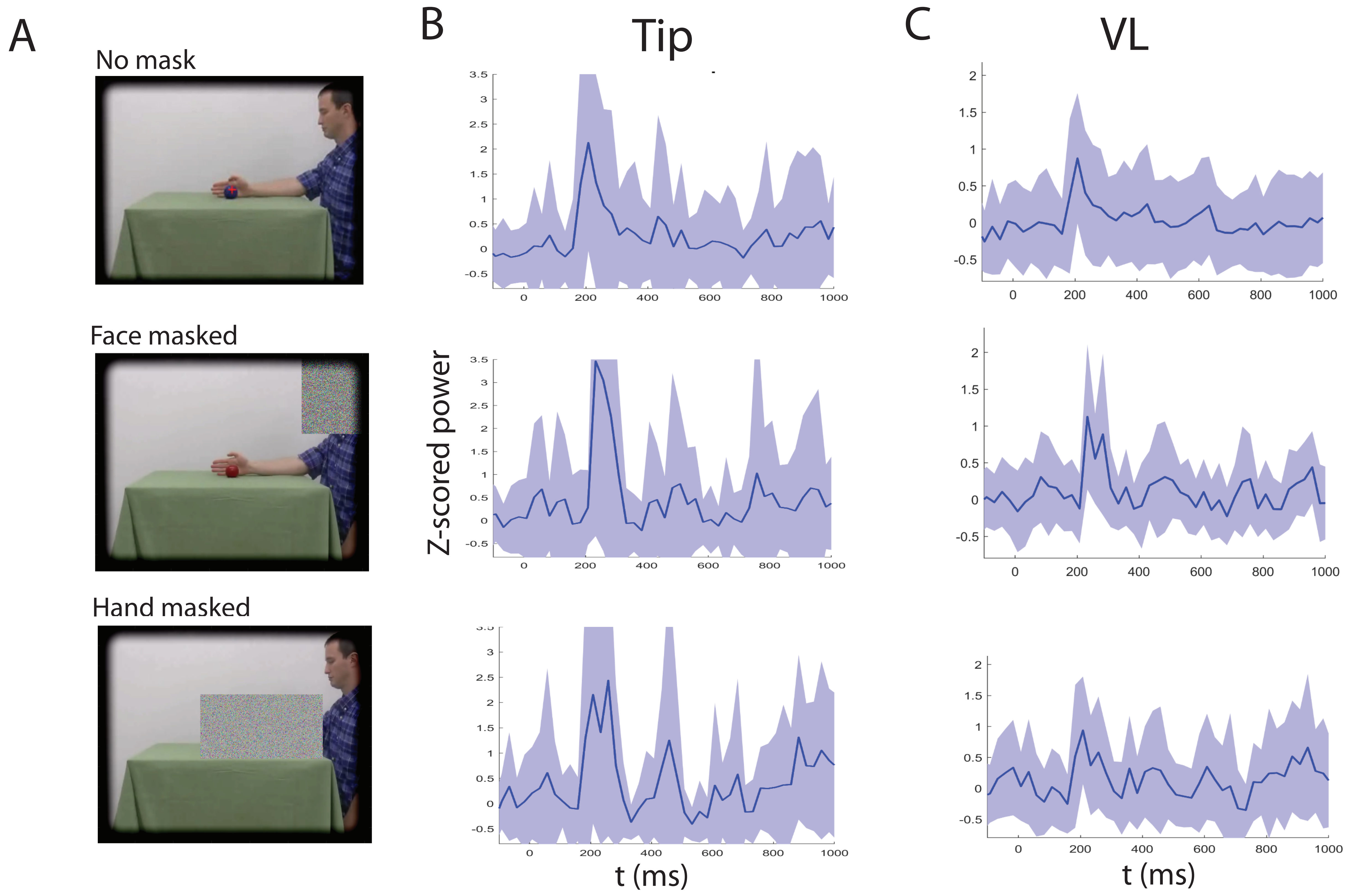
A
No mask
Face masked
Hand masked
B
Tip
C
VL
Z-scored power
t (ms)
t (ms)

Figure S3-1

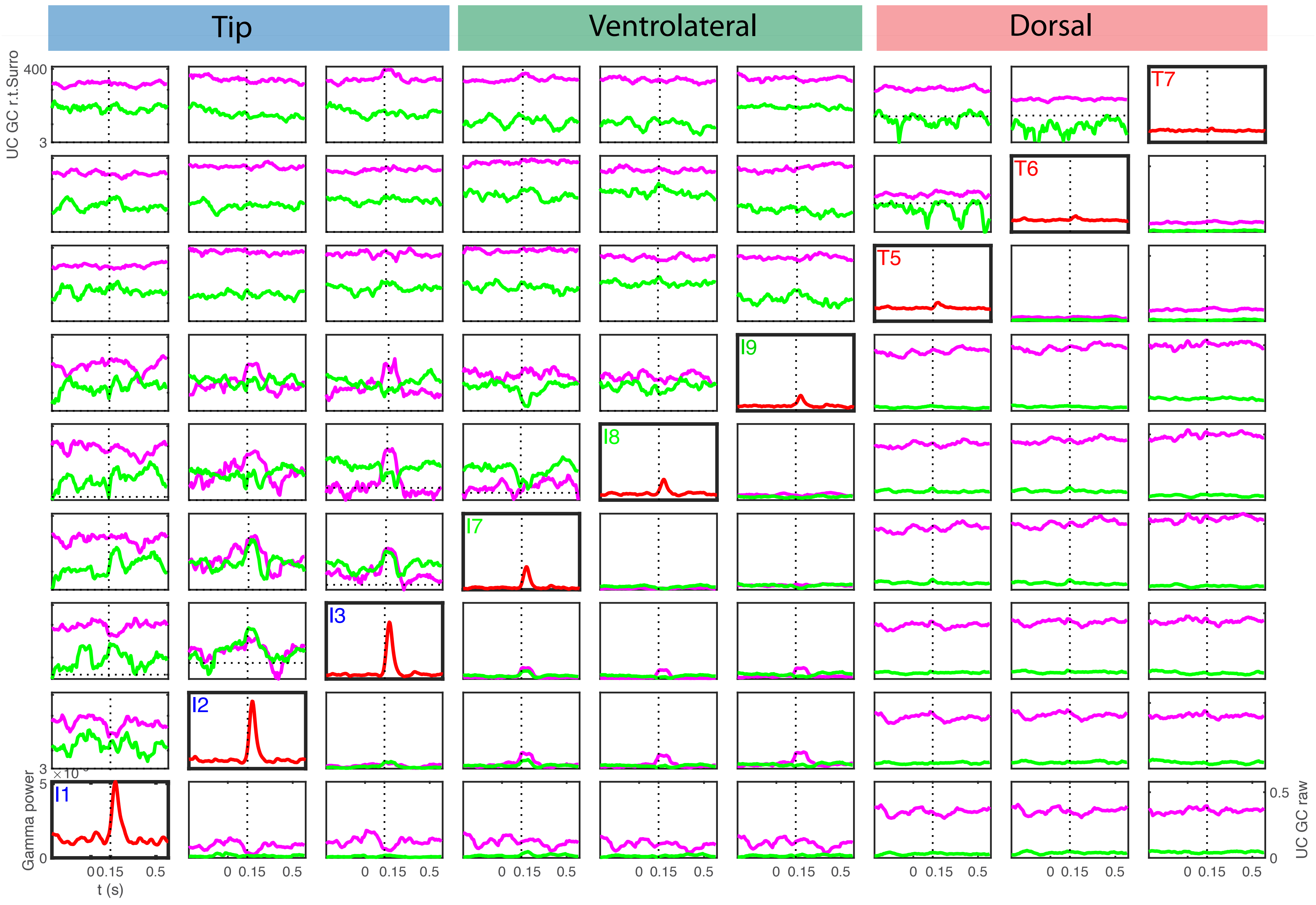
Tip
Ventrolateral
Dorsal
UC GC r.t.Surro
400
3
Gamma power
×10
5
0
I1
I2
I3
I7
I8
I9
T5
T6
T7
0 0.15 0.5
t (s)
UC GC raw
0.5
0

Figure S3-2

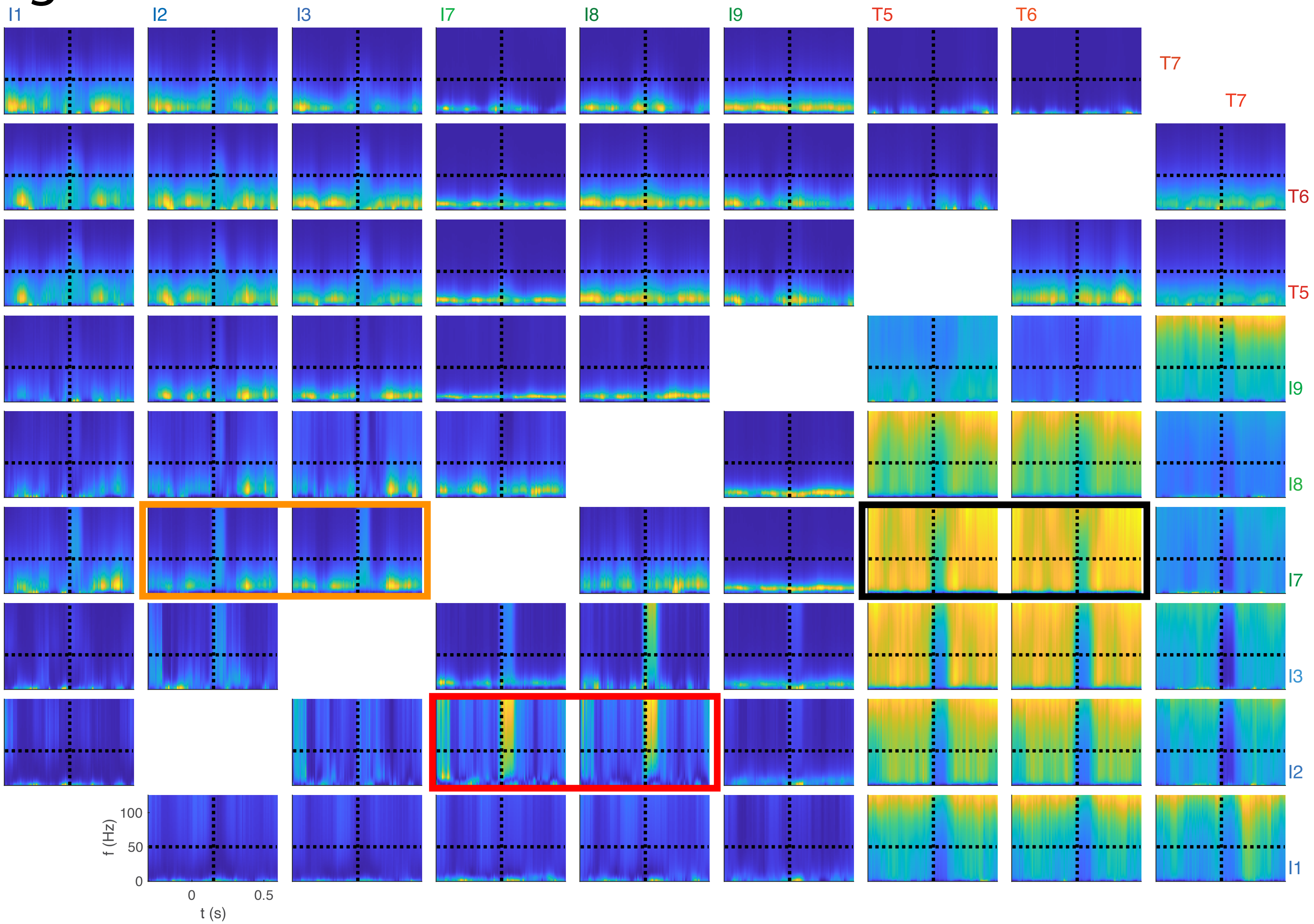

I1
I2
I3
I7
I8
I9
T5
T6
T7
T7
T6
T5
I9
I8
I7
I3
I2
I1
100
50
0
f (Hz)
0
0.5
t (s)

Figure S3-3

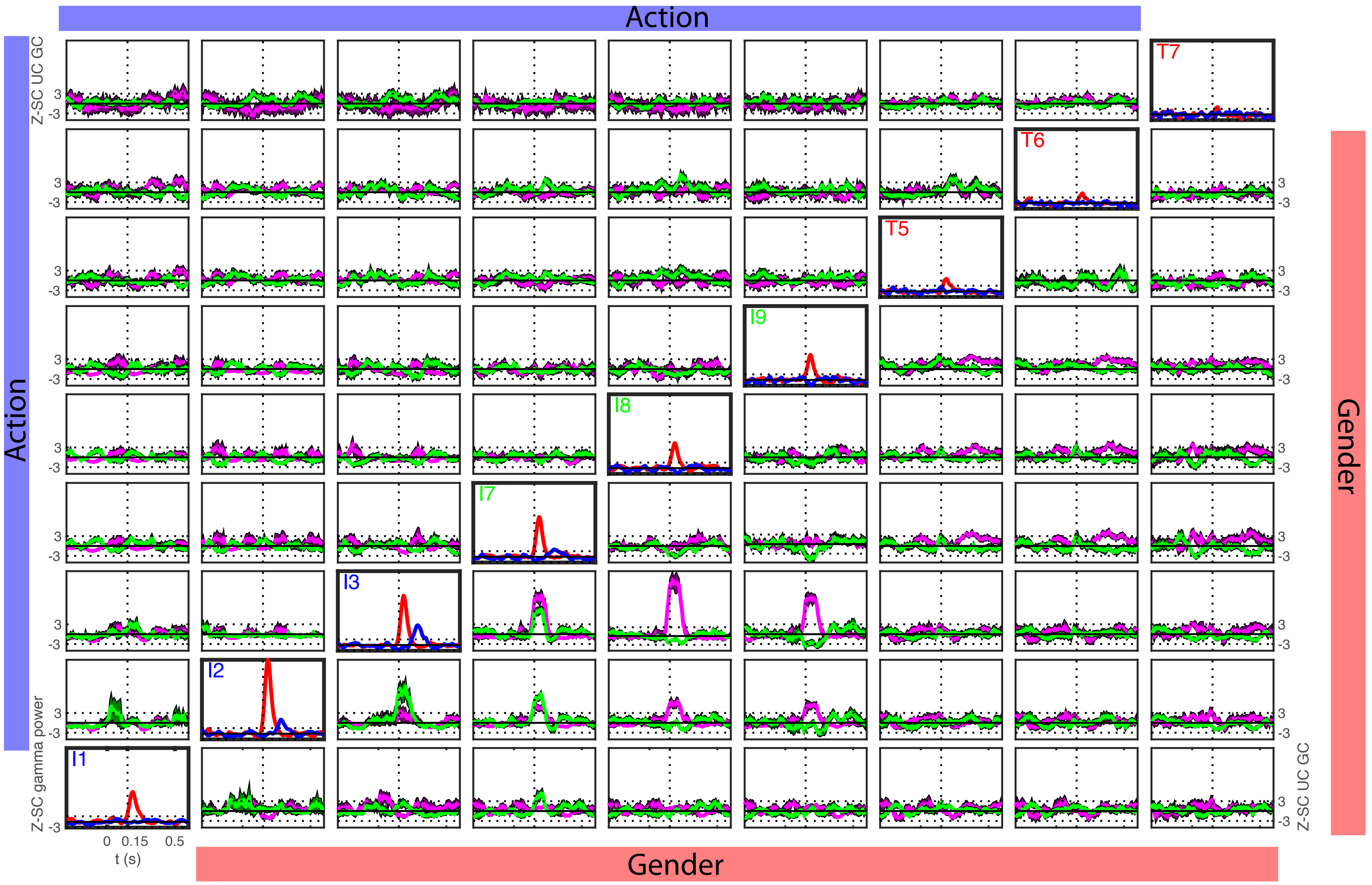

Action
Action
Gender
Gender
Z-SC UC GC
Z-SC gamma power
Z-SC UC GC
3
-3
0 0.15 0.5
t (s)
I1
I2
I3
I7
I8
I9
T5
T6
T7

Figure S3-4

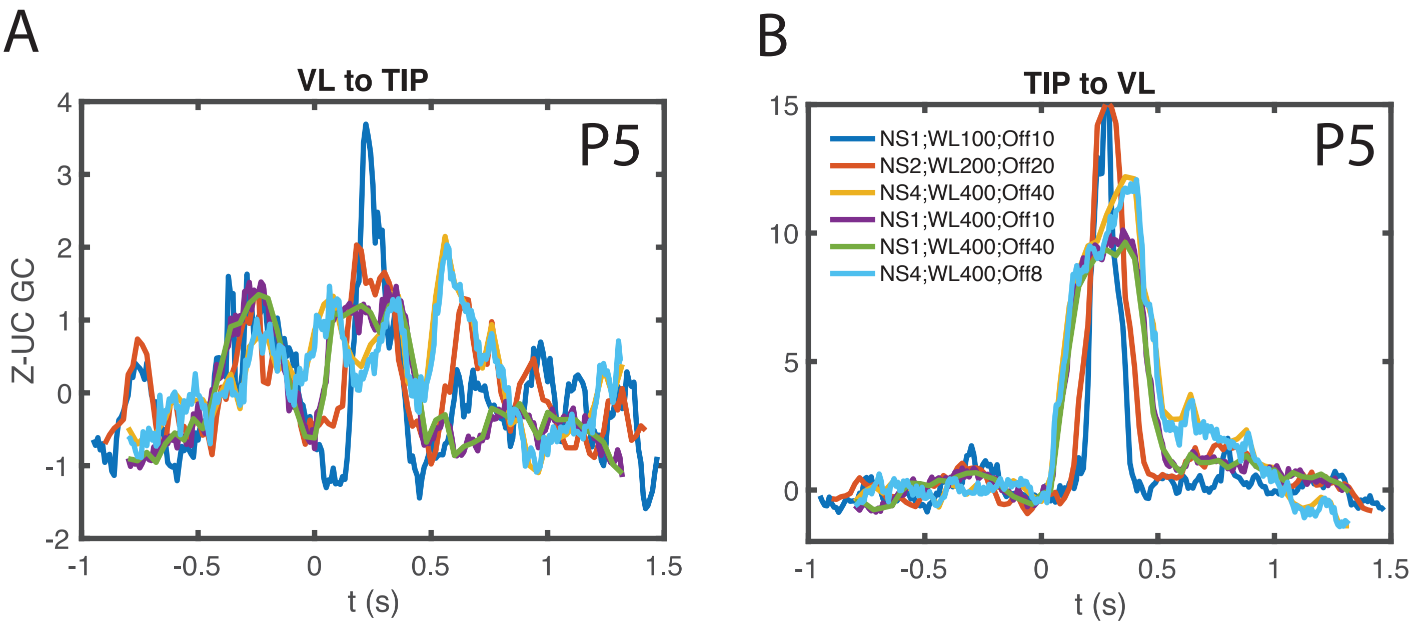

A
VL to TIP
P5
Z-UC GC
t (s)
B
TIP to VL
P5
NS1;WL100;Off10
NS2;WL200;Off20
NS4;WL400;Off40
NS1;WL400;Off10
NS1;WL400;Off40
NS4;WL400;Off8
t (s)

Figure S3-5

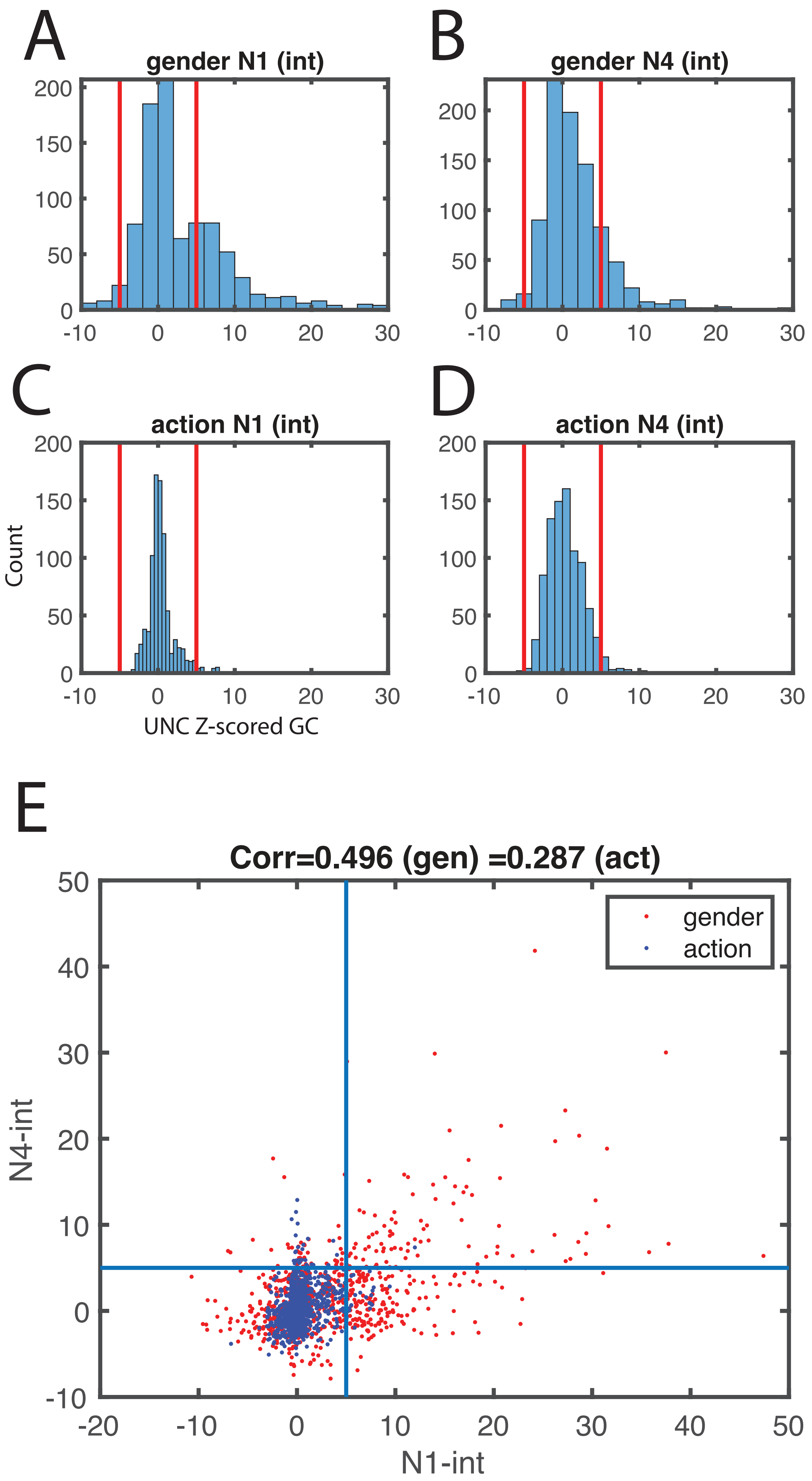

A
gender N1 (int)
B
gender N4 (int)
C
action N1 (int)
D
action N4 (int)
Count
UNC Z-scored GC
E
Corr=0.496 (gen) =0.287 (act)
gender
action
N4-int
N1-int

Figure S3-6

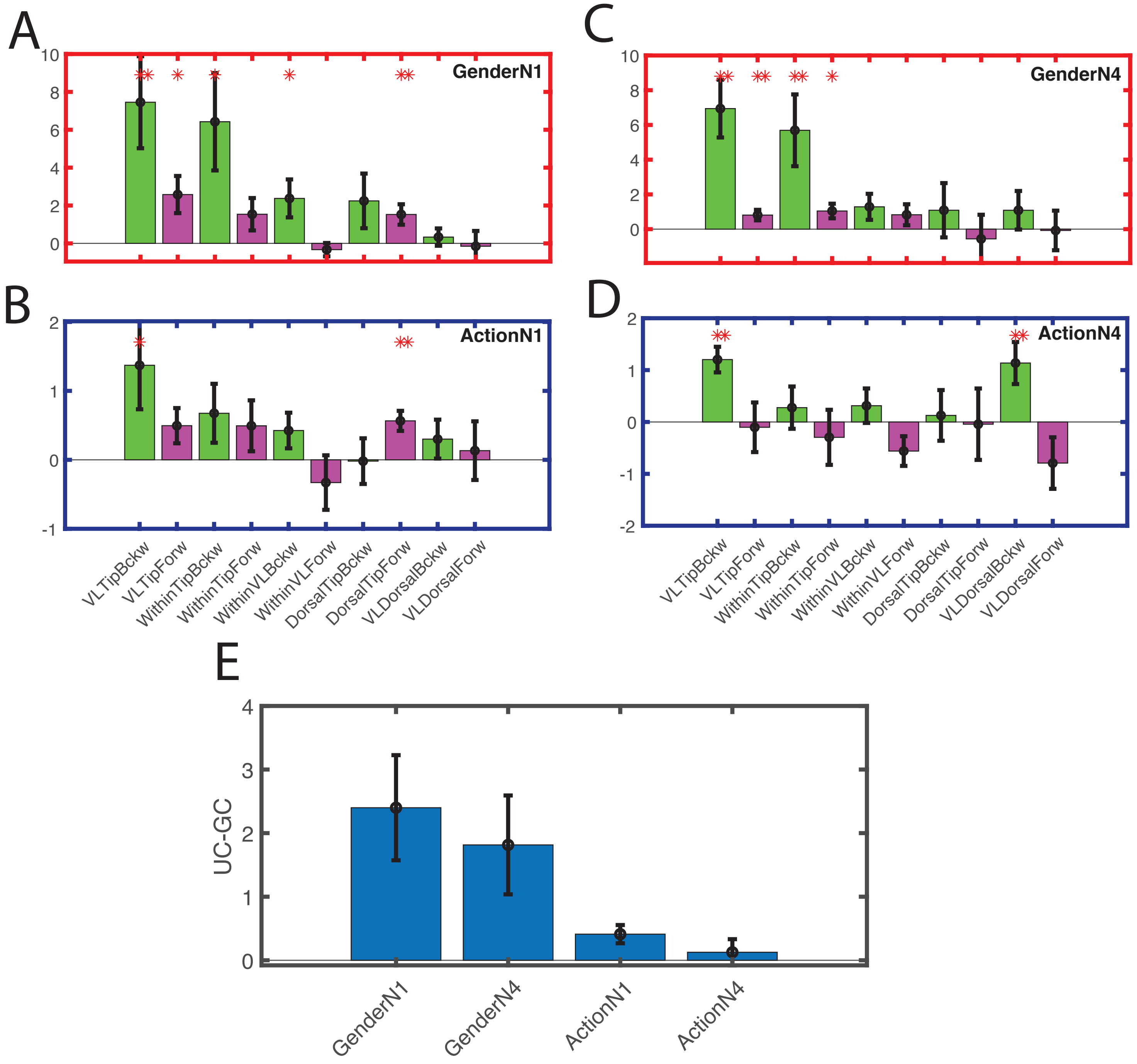

A
B
C
D
E
GenderN1
ActionN1
GenderN4
ActionN4
VLTipBckw
VLTipForw
WithinTipBckw
WithinTipForw
WithinVLBckw
WithinVLForw
DorsalTipBckw
DorsalTipForw
VLDorsalBckw
VLDorsalForw
UC-GC

Figure S3-7

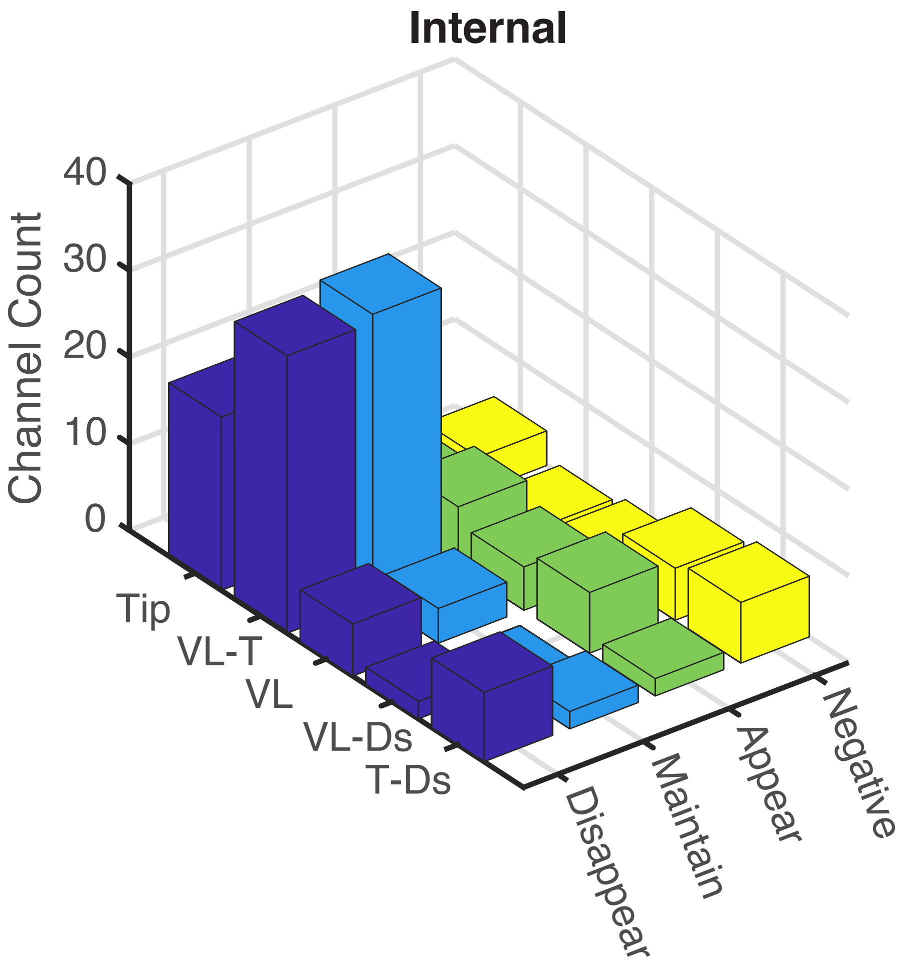

Internal
Channel Count
40
30
20
10
0
Tip
VL-T
VL
VL-Ds
T-Ds
Disappear
Maintain
Appear
Negative

Figure S3-8

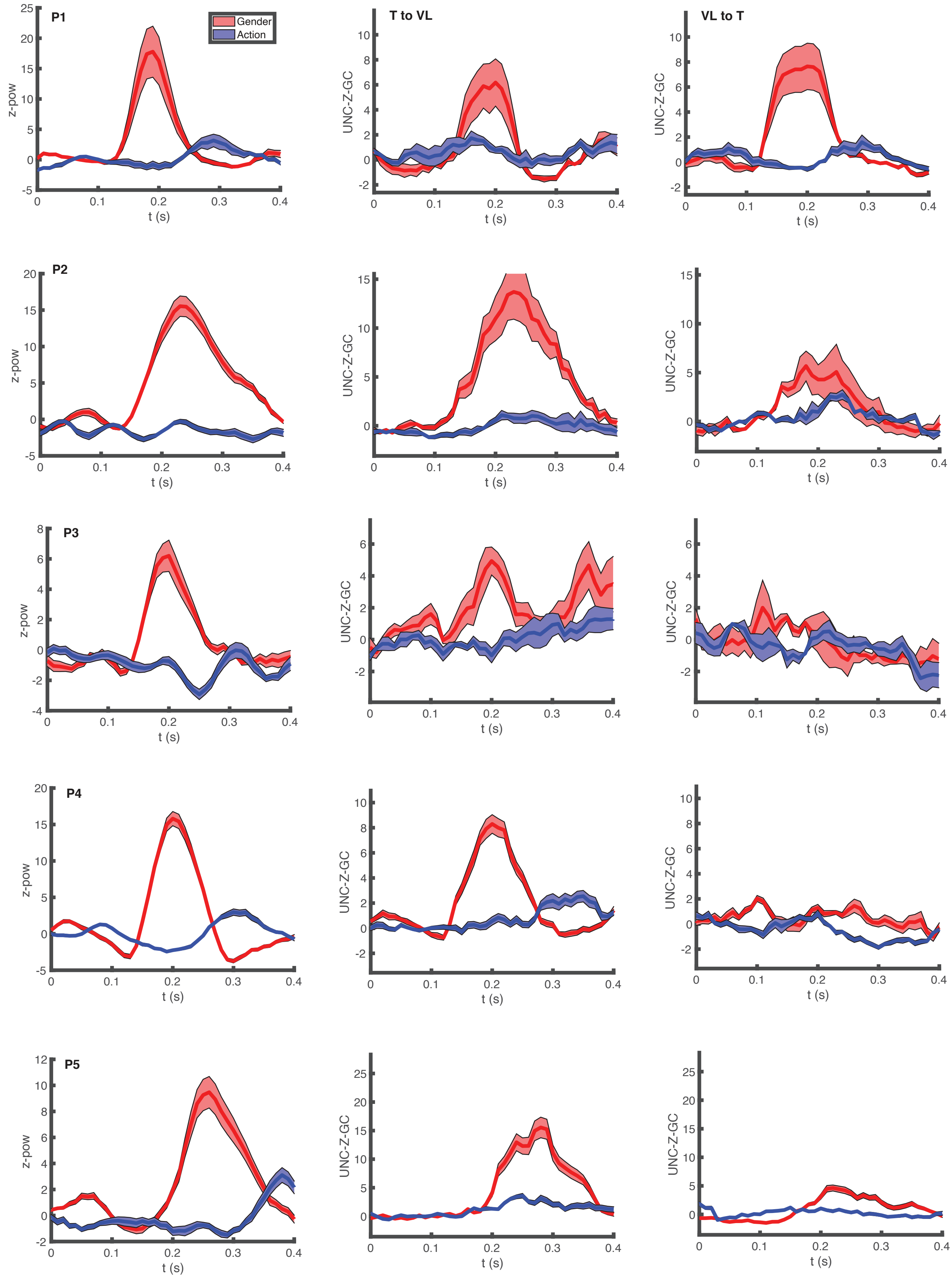

Figure S4-1

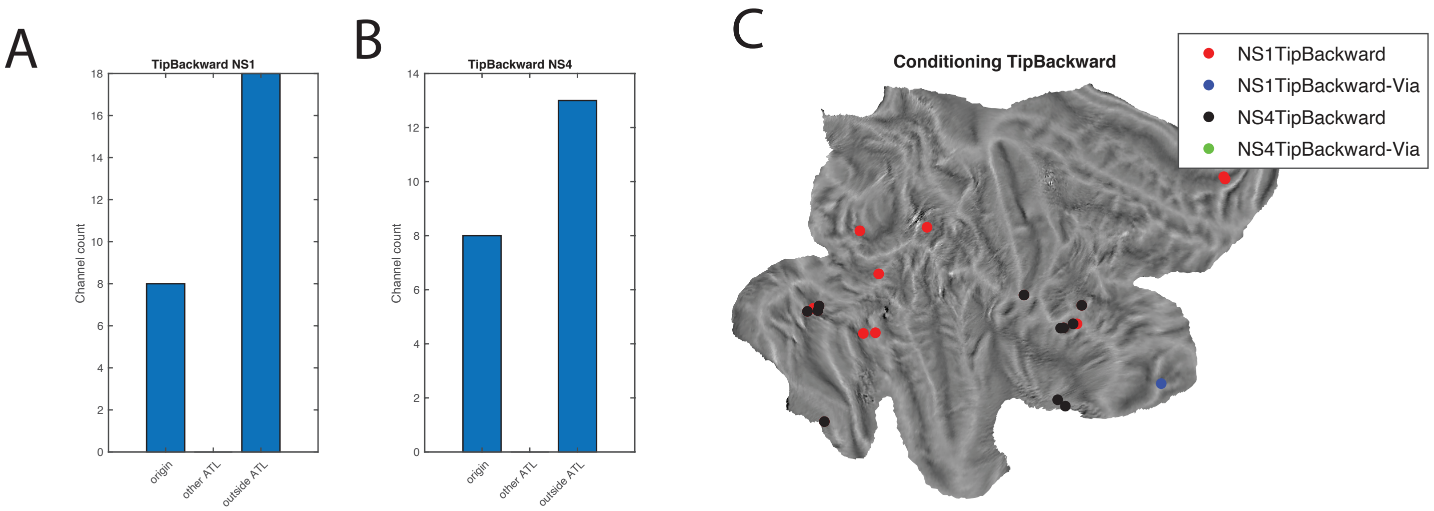
A
TipBackward NS1
Channel count
0
2
4
6
8
10
12
14
16
18
origin
other ATL
outside ATL
B
TipBackward NS4
Channel count
0
2
4
6
8
10
12
14
origin
other ATL
outside ATL
C
Conditioning TipBackward
NS1TipBackward
NS1TipBackward-Via
NS4TipBackward
NS4TipBackward-Via

Figure S5-1

A

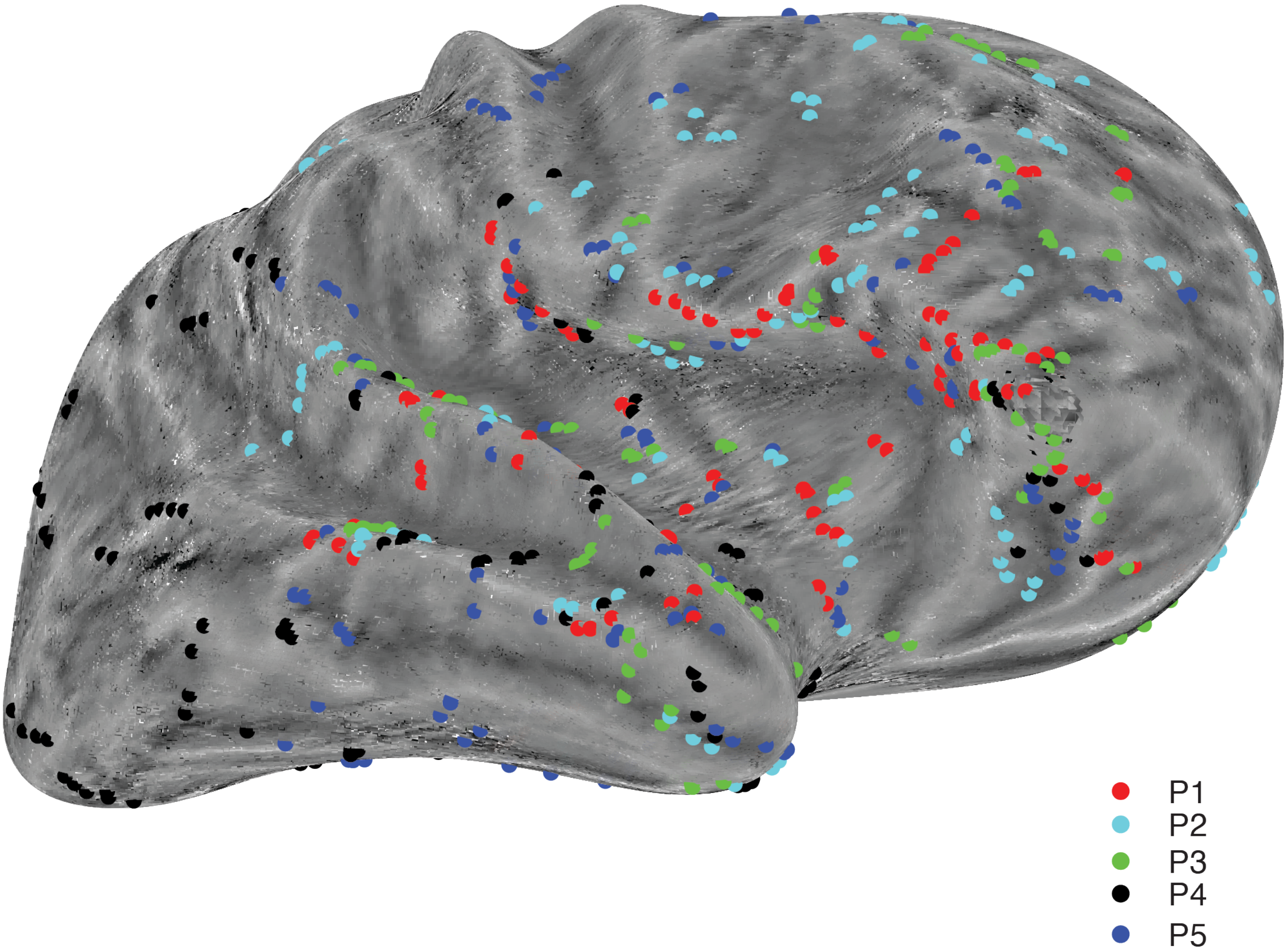

B

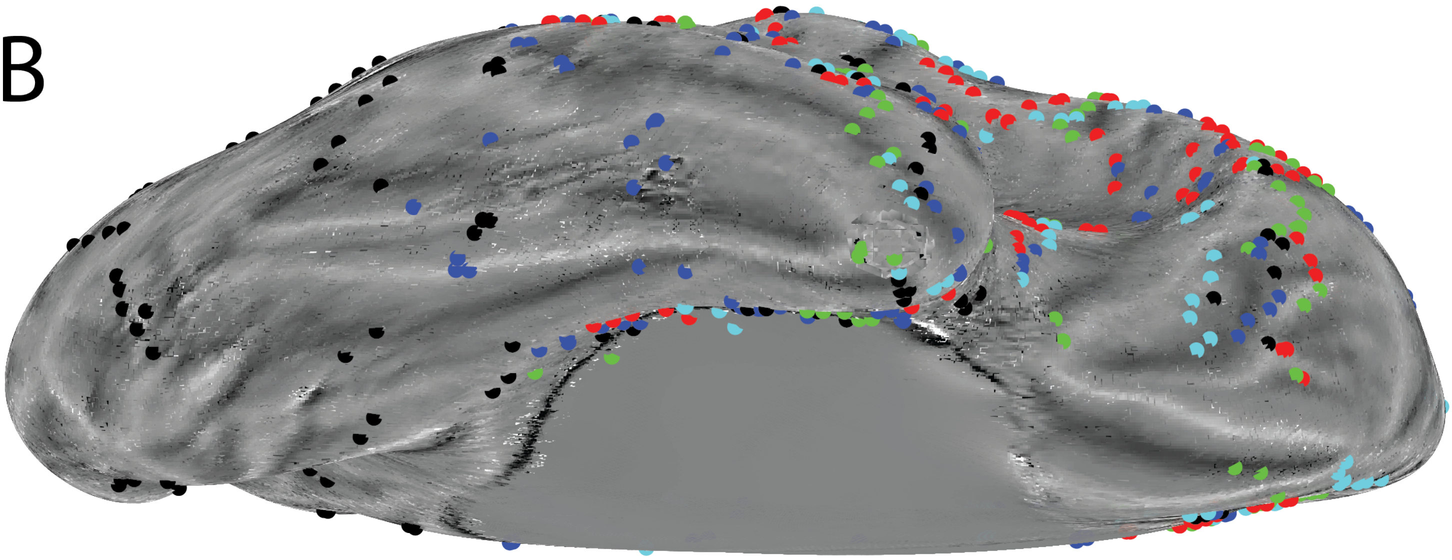

Figure S5-2

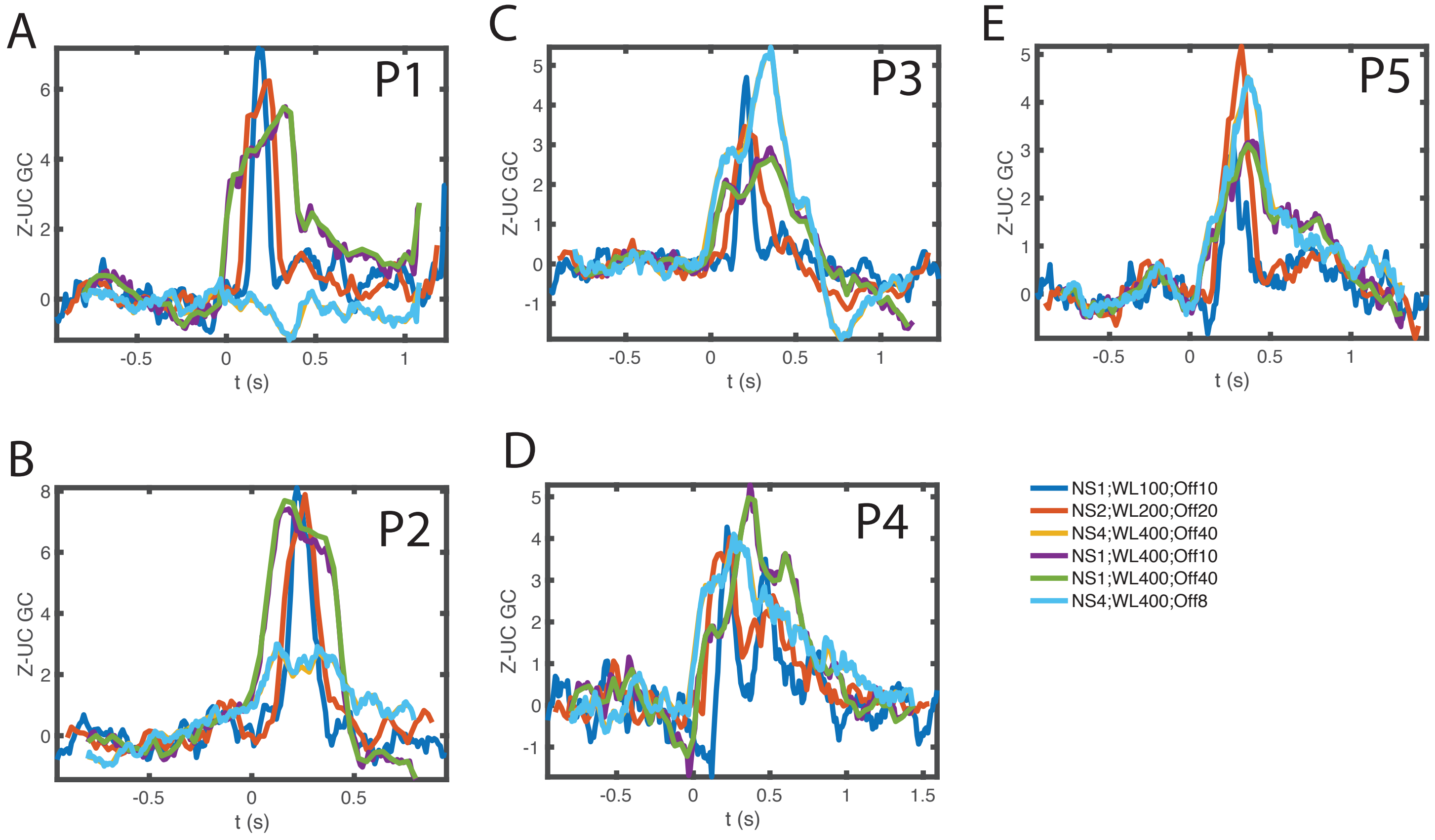
A
P1
B
P2
C
P3
D
P4
E
P5
Z-UC GC
t (s)
NS1;WL100;Off10
NS2;WL200;Off20
NS4;WL400;Off40
NS1;WL400;Off10
NS1;WL400;Off40
NS4;WL400;Off8

Figure S5-3

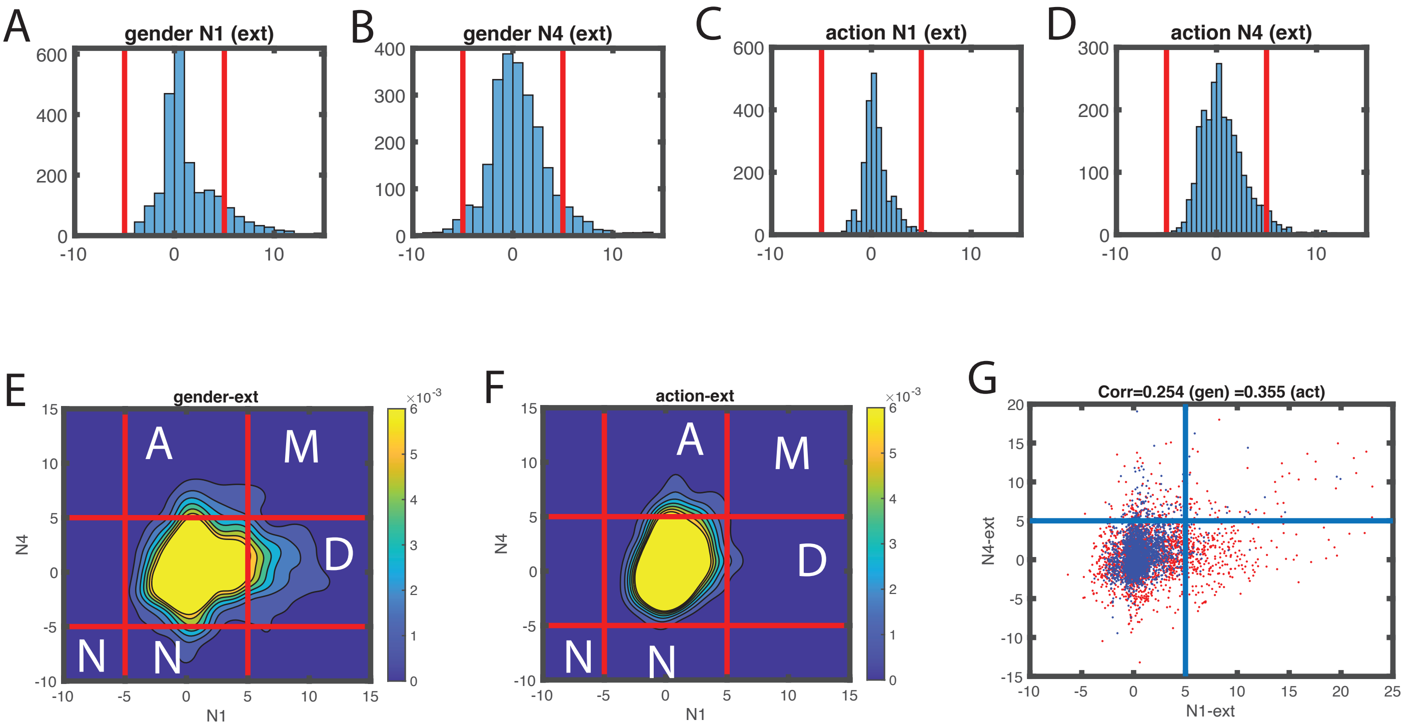
A
gender N1 (ext)
B
gender N4 (ext)
C
action N1 (ext)
D
action N4 (ext)
E
gender-ext
N1
N4
A
M
D
N
N
F
action-ext
N1
N4
A
M
D
N
N
G
Corr=0.254 (gen) =0.355 (act)
N1-ext
N4-ext

Figure S5-4

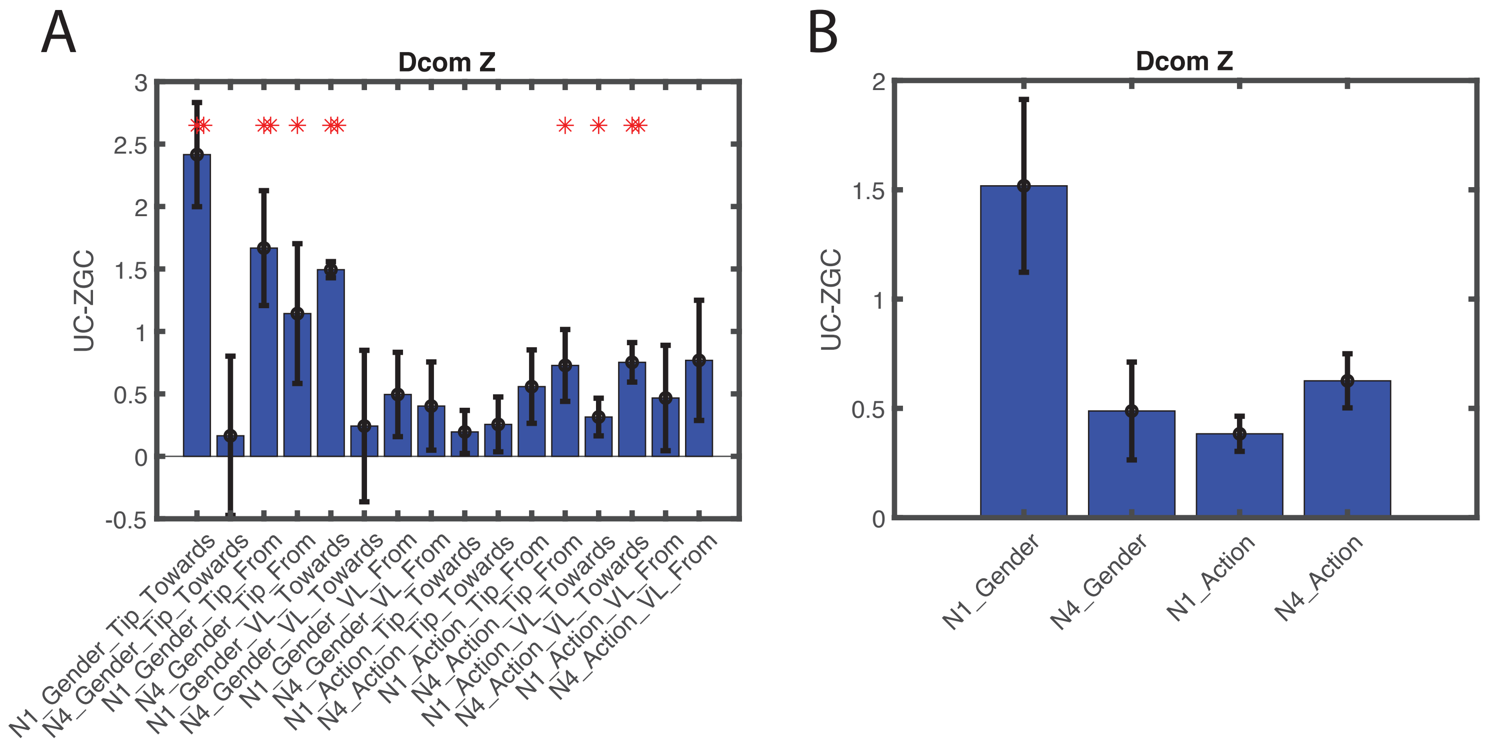
A
Dcom Z
UC-ZGC
N1_Gender_Tip_Towards
N4_Gender_Tip_Towards
N1_Gender_Tip_From
N4_Gender_Tip_From
N1_Gender_VL_Towards
N4_Gender_VL_Towards
N1_Gender_VL_From
N4_Gender_VL_From
N1_Action_Tip_Towards
N4_Action_Tip_Towards
N1_Action_Tip_From
N4_Action_Tip_From
N1_Action_VL_Towards
N4_Action_VL_Towards
N1_Action_VL_From
N4_Action_VL_From
B
Dcom Z
UC-ZGC
N1_Gender
N4_Gender
N1_Action
N4_Action

Figure S5-5

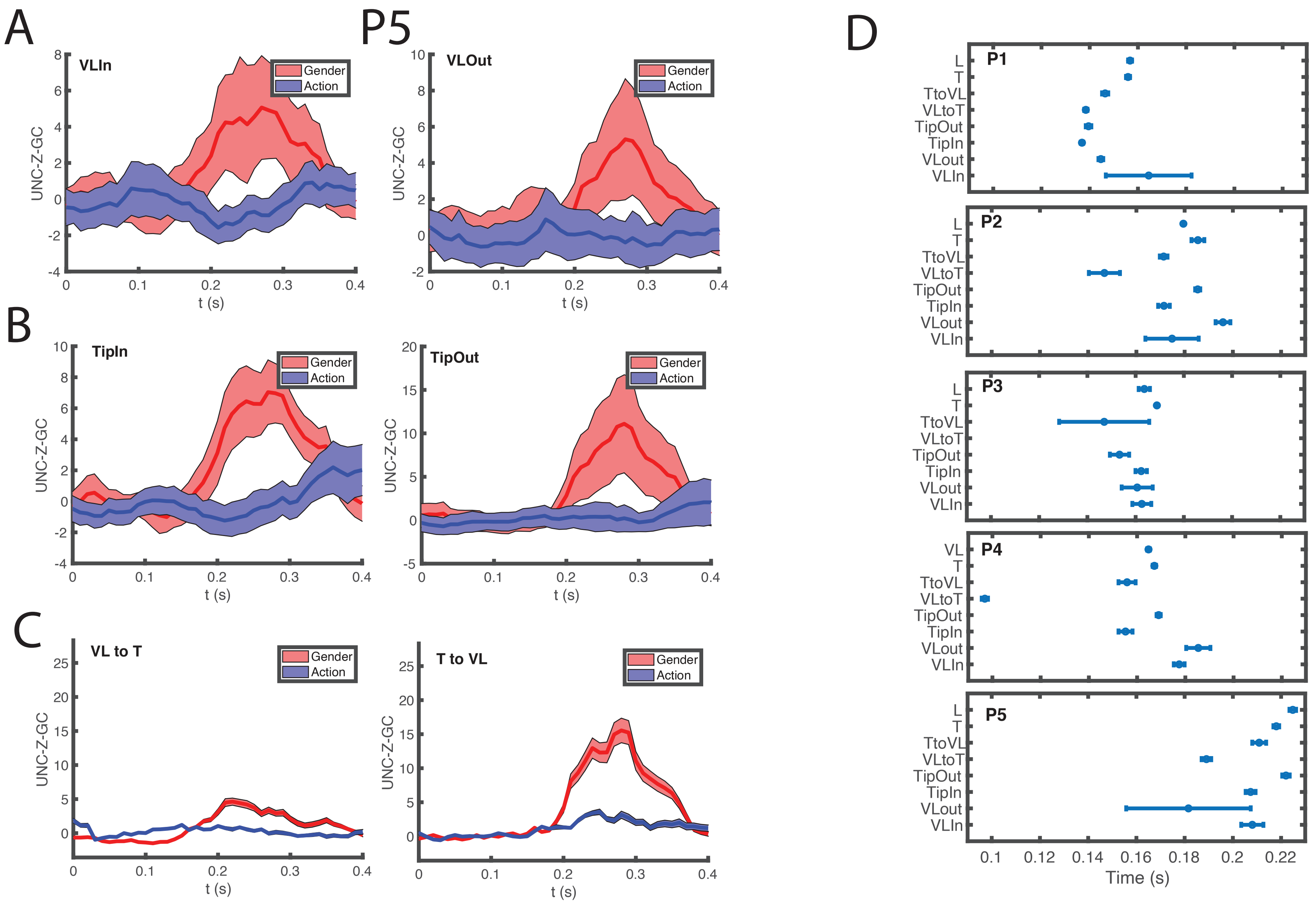
A
P5
VLIn
VLOut
Gender
Action
UNC-Z-GC
t (s)
B
TipIn
TipOut
C
VL to T
T to VL
D
P1
P2
P3
P4
P5
L
T
TtoVL
VLtoT
TipOut
TipIn
VLout
VLIn
VL
Time (s)

Figure S5-6

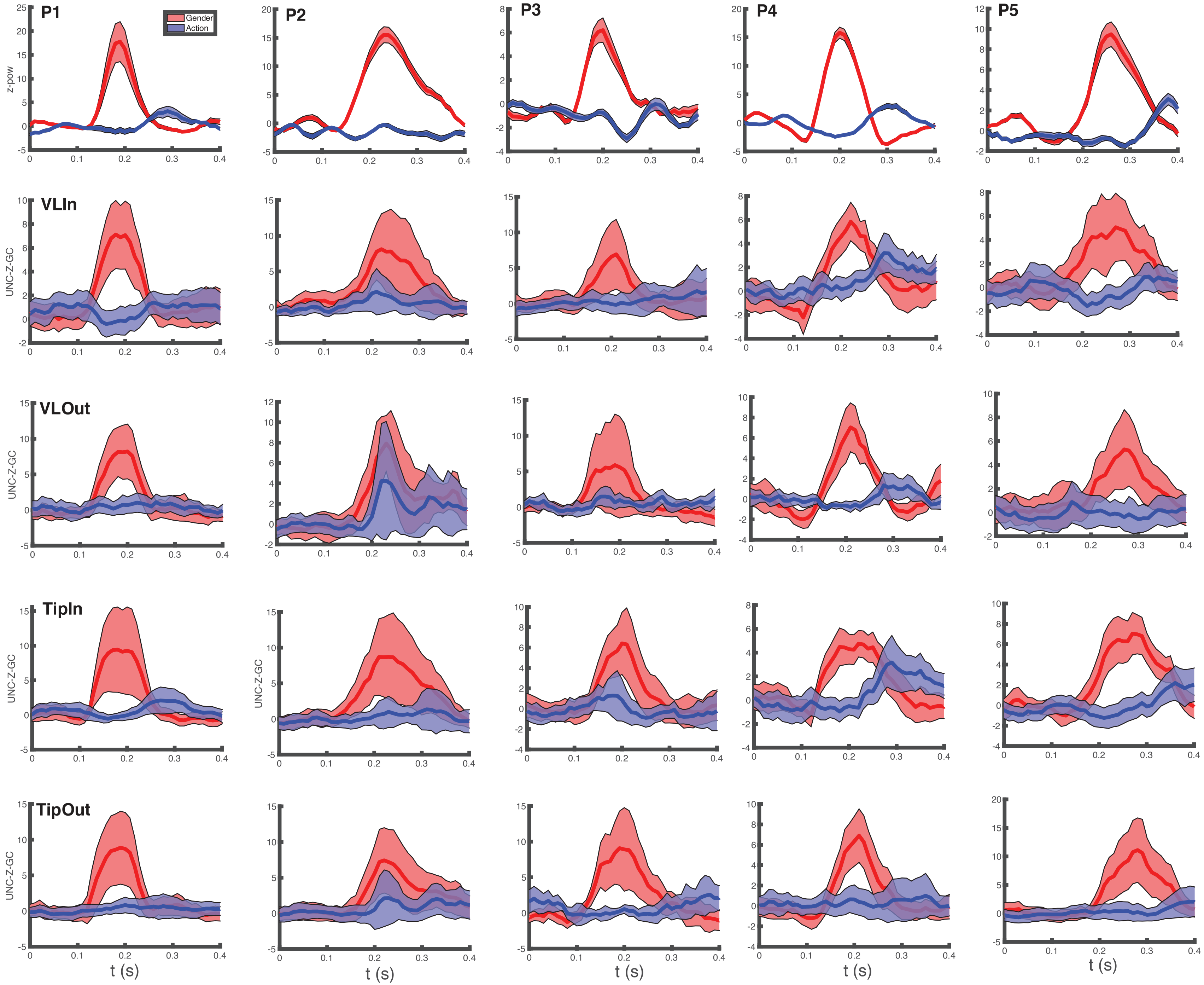

P1
P2
P3
P4
P5
VLIn
VLOut
TipIn
TipOut
Gender
Action
z-pow
UNC-Z-GC
t (s)

Figure S6-1

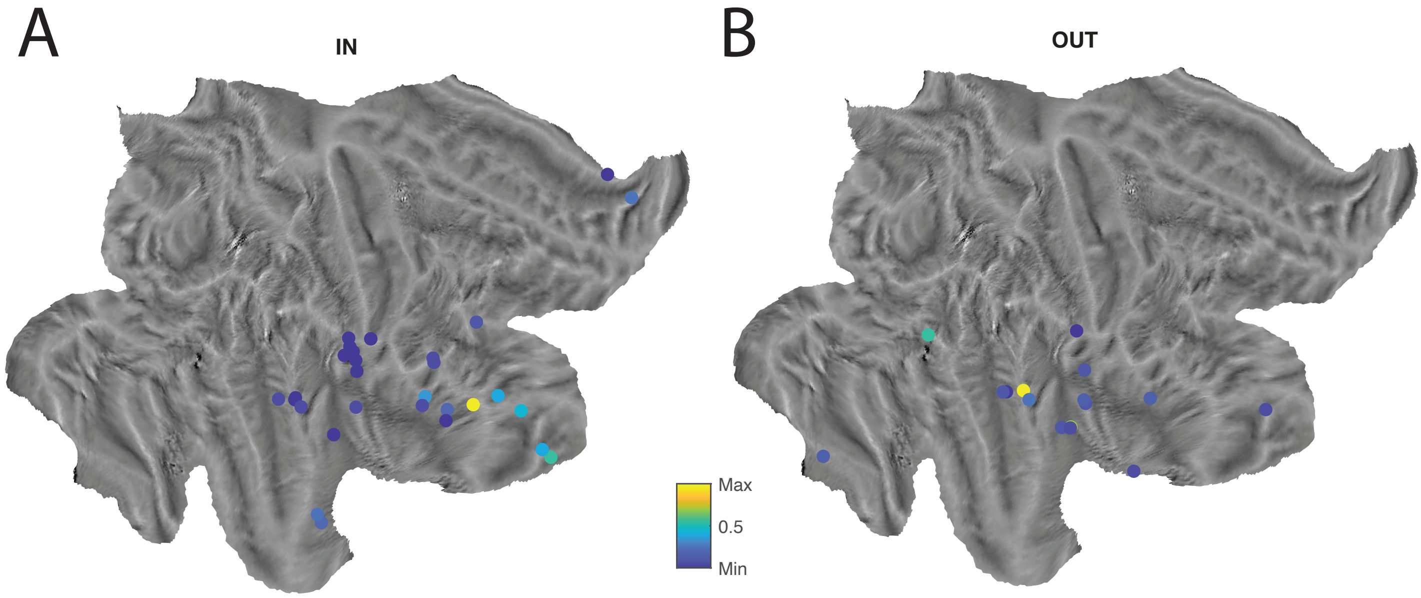

Figure S6-2

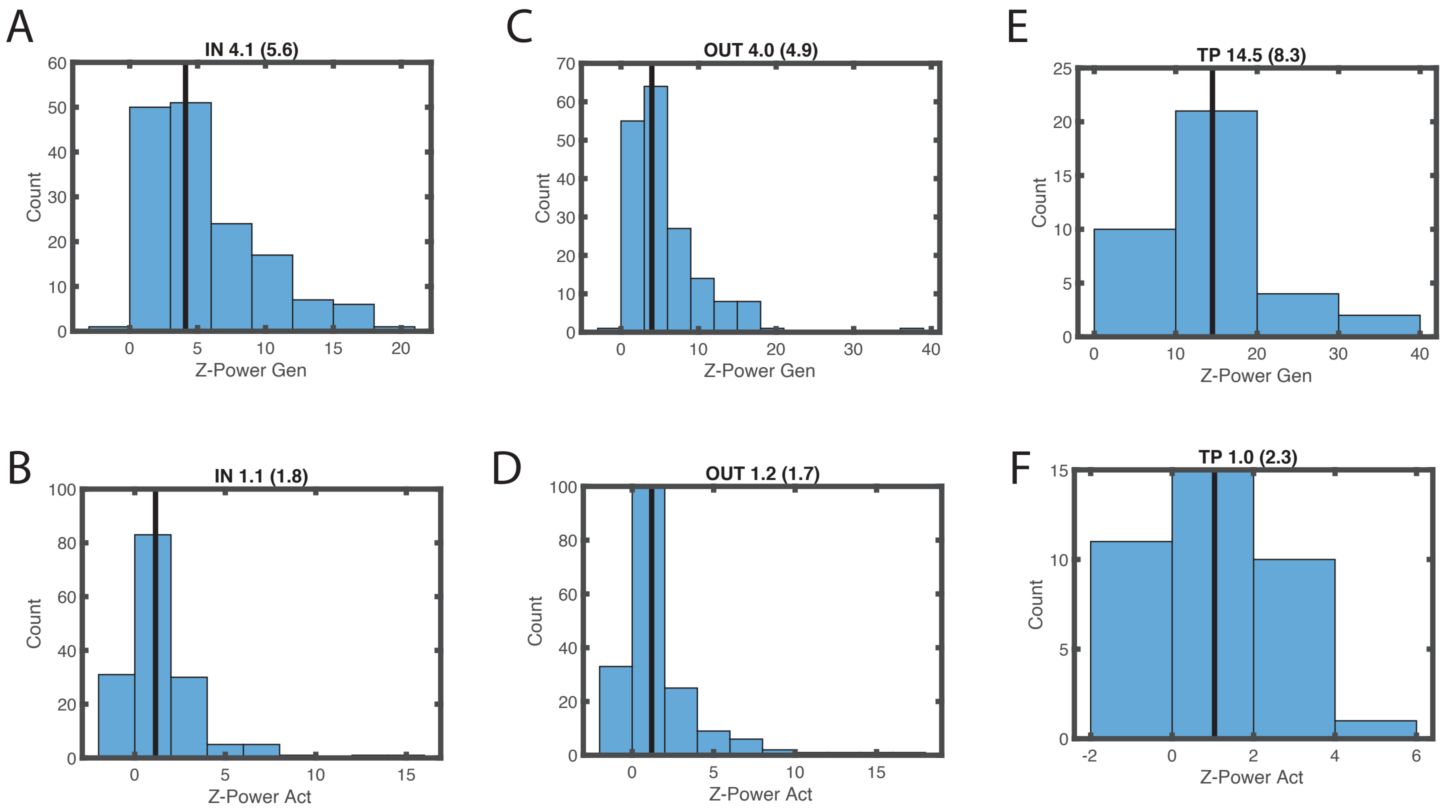
A
IN 4.1 (5.6)
Count
Z-Power Gen
C
OUT 4.0 (4.9)
Count
Z-Power Gen
E
TP 14.5 (8.3)
Count
Z-Power Gen
B
IN 1.1 (1.8)
Count
Z-Power Act
D
OUT 1.2 (1.7)
Count
Z-Power Act
F
TP 1.0 (2.3)
Count
Z-Power Act

Figure S6-3

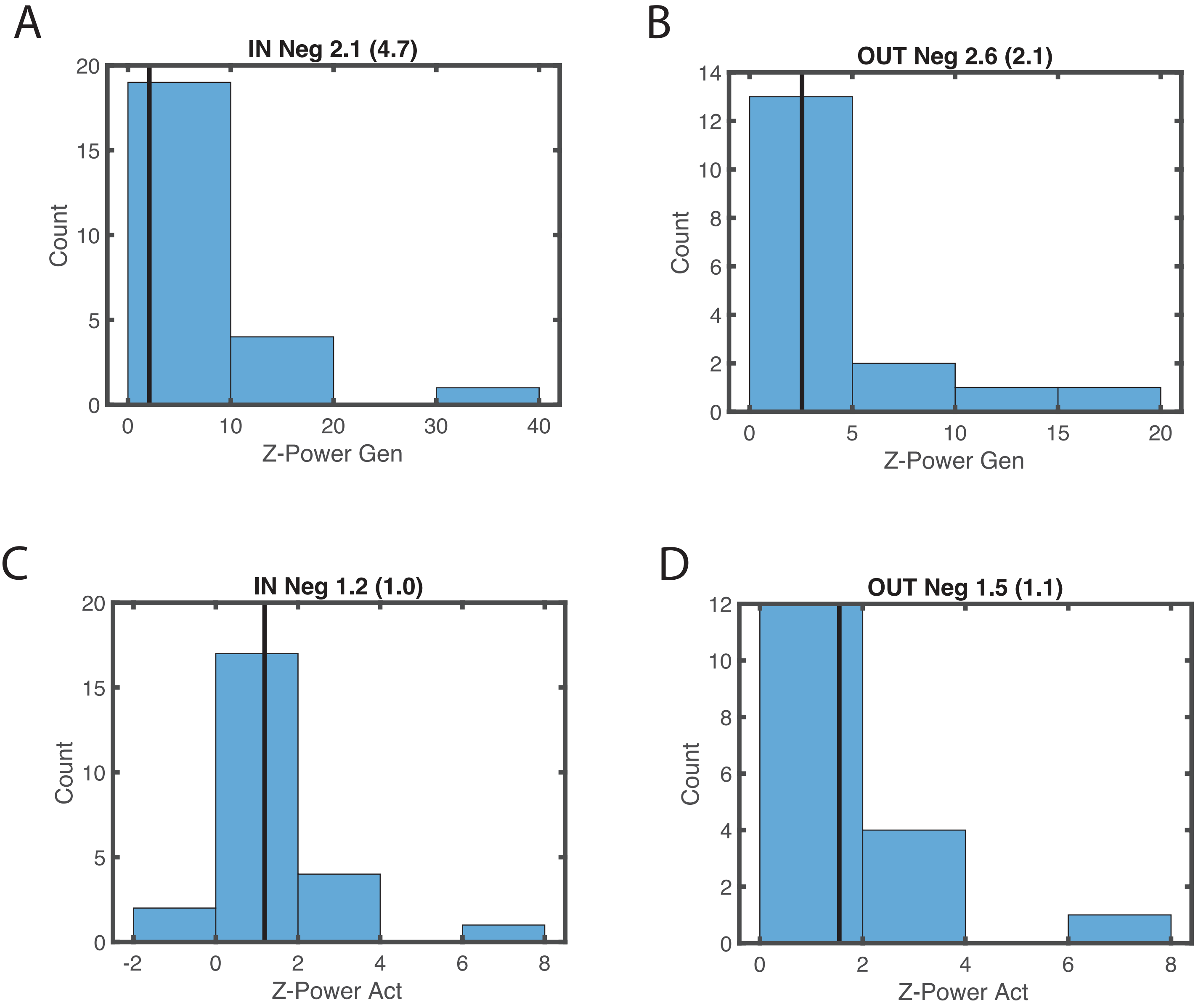

A
IN Neg 2.1 (4.7)
Count
Z-Power Gen
B
OUT Neg 2.6 (2.1)
Count
Z-Power Gen
C
IN Neg 1.2 (1.0)
Count
Z-Power Act
D
OUT Neg 1.5 (1.1)
Count
Z-Power Act

Figure S6-4

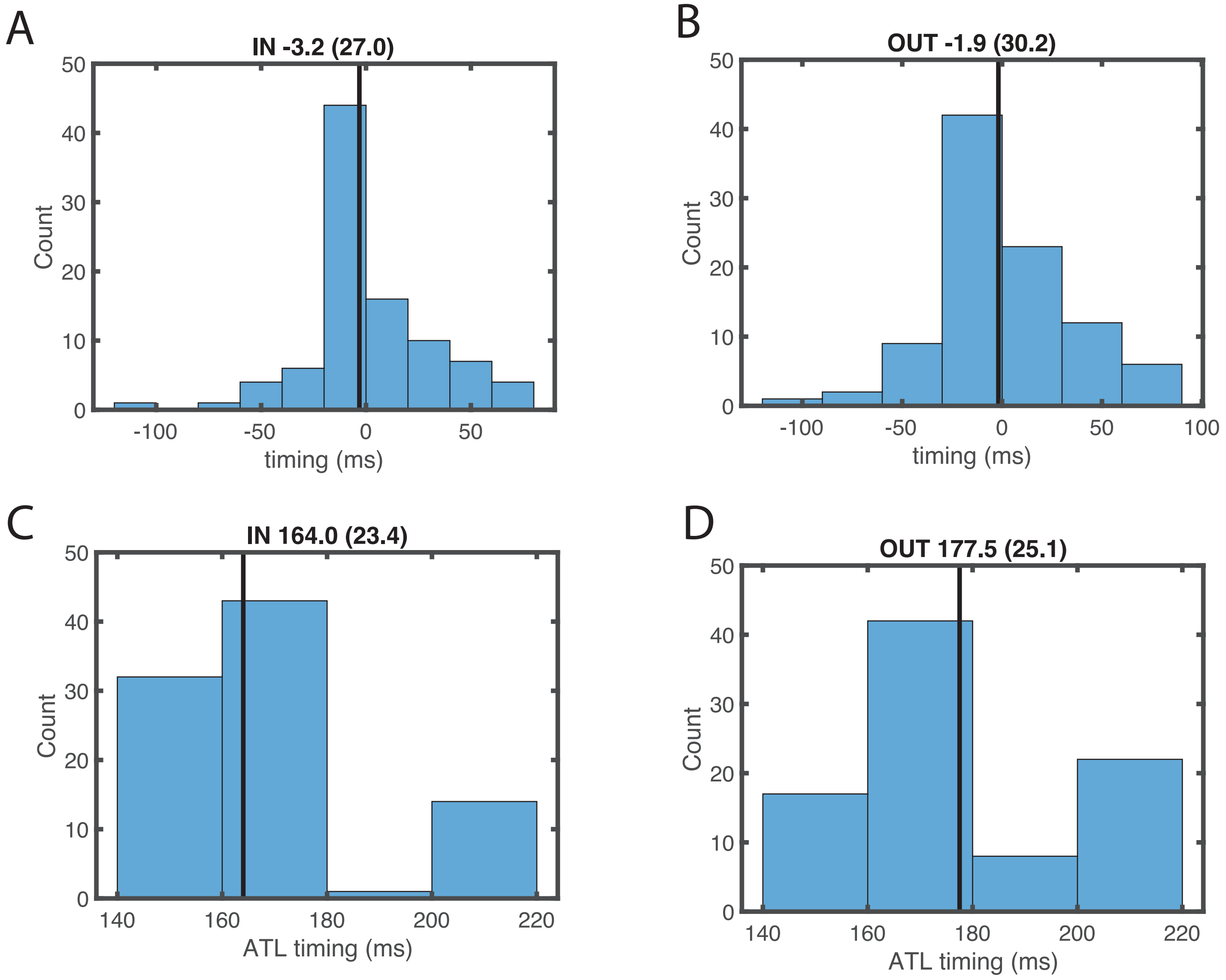
A
IN -3.2 (27.0)
Count
timing (ms)
B
OUT -1.9 (30.2)
Count
timing (ms)
C
IN 164.0 (23.4)
Count
ATL timing (ms)
D
OUT 177.5 (25.1)
Count
ATL timing (ms)

Figure S6-5

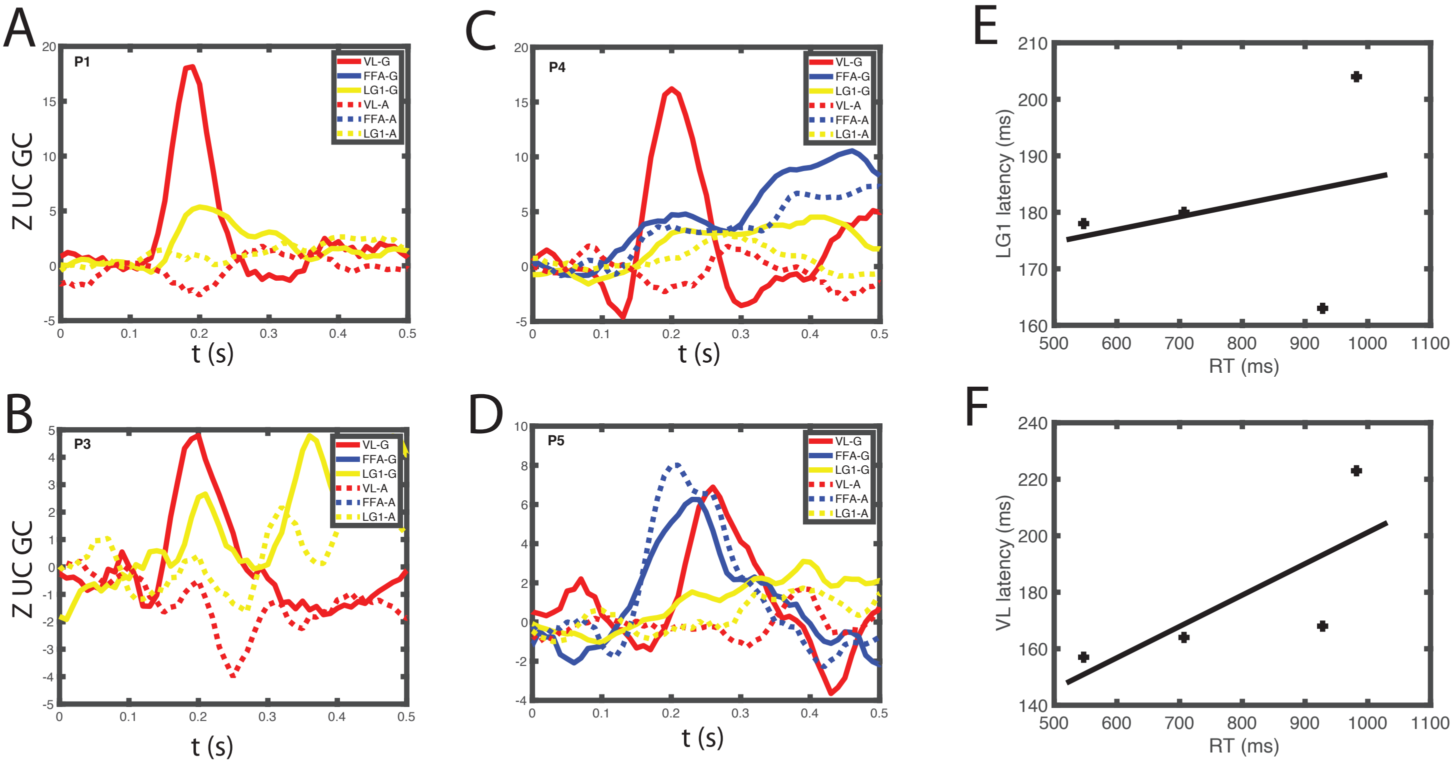

A
B
C
D
E
F
P1
P3
P4
P5
VL-G
FFA-G
LG1-G
VL-A
FFA-A
LG1-A
Z UC GC
t (s)
LG1 latency (ms)
VL latency (ms)
RT (ms)

Figure S6-6

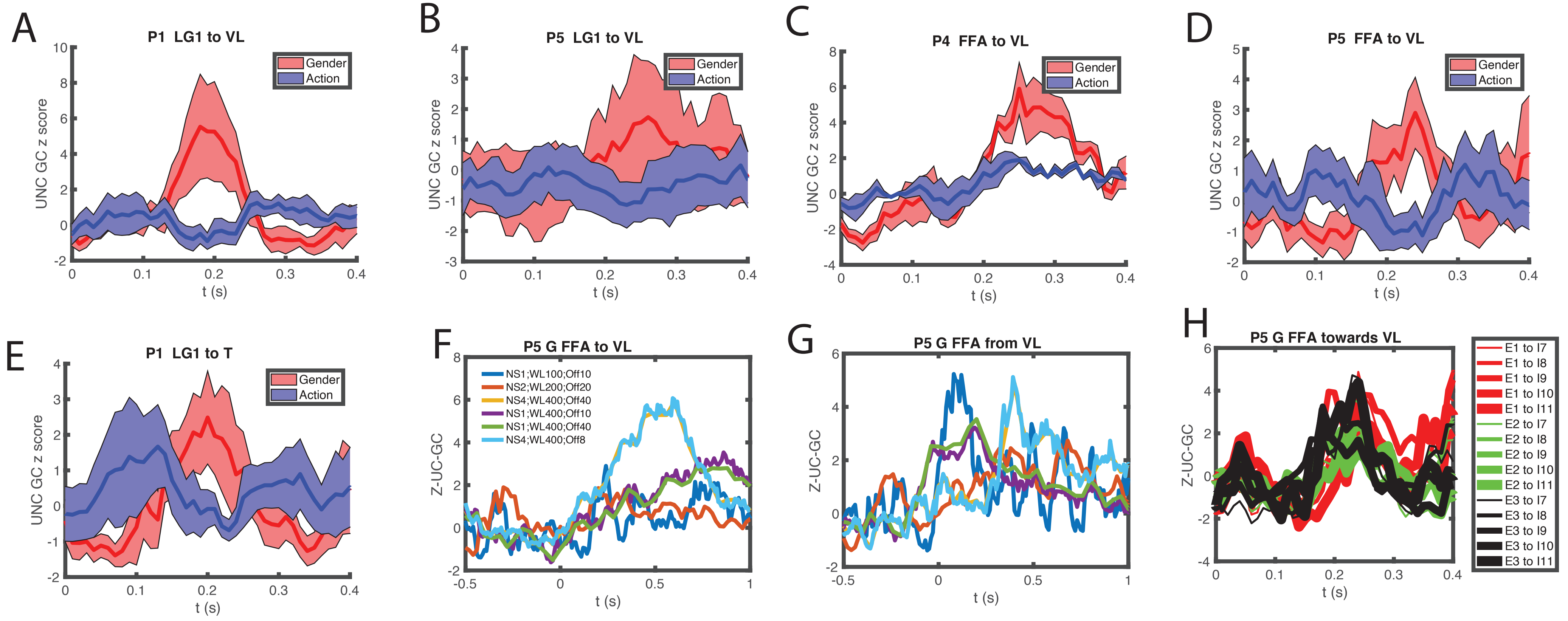
A
P1 LG1 to VL
Gender
Action
UNC GC z score
t (s)
B
P5 LG1 to VL
C
P4 FFA to VL
D
P5 FFA to VL
E
P1 LG1 to T
F
P5 G FFA to VL
NS1;WL100;Off10
NS2;WL200;Off20
NS4;WL400;Off40
NS1;WL400;Off10
NS1;WL400;Off40
NS4;WL400;Off8
Z-UC-GC
G
P5 G FFA from VL
H
P5 G FFA towards VL
E1 to I7
E1 to I8
E1 to I9
E1 to I10
E1 to I11
E2 to I7
E2 to I8
E2 to I9
E2 to I10
E2 to I11
E3 to I7
E3 to I8
E3 to I9
E3 to I10
E3 to I11

Figure S6-7

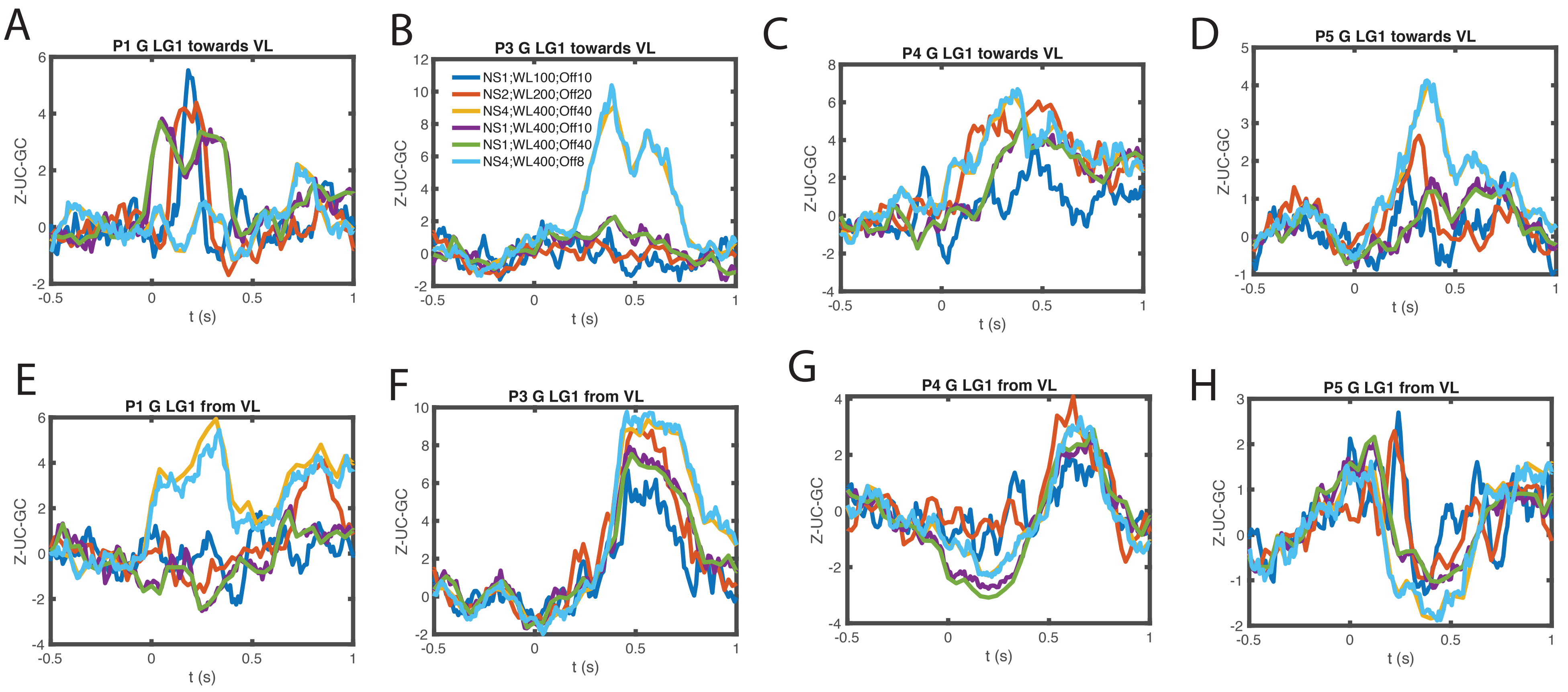
A
P1 G LG1 towards VL
B
P3 G LG1 towards VL
NS1;WL100;Off10
NS2;WL200;Off20
NS4;WL400;Off40
NS1;WL400;Off10
NS1;WL400;Off40
NS4;WL400;Off8
C
P4 G LG1 towards VL
D
P5 G LG1 towards VL
E
P1 G LG1 from VL
F
P3 G LG1 from VL
G
P4 G LG1 from VL
H
P5 G LG1 from VL
Z-UC-GC
t (s)

Figure S6-8

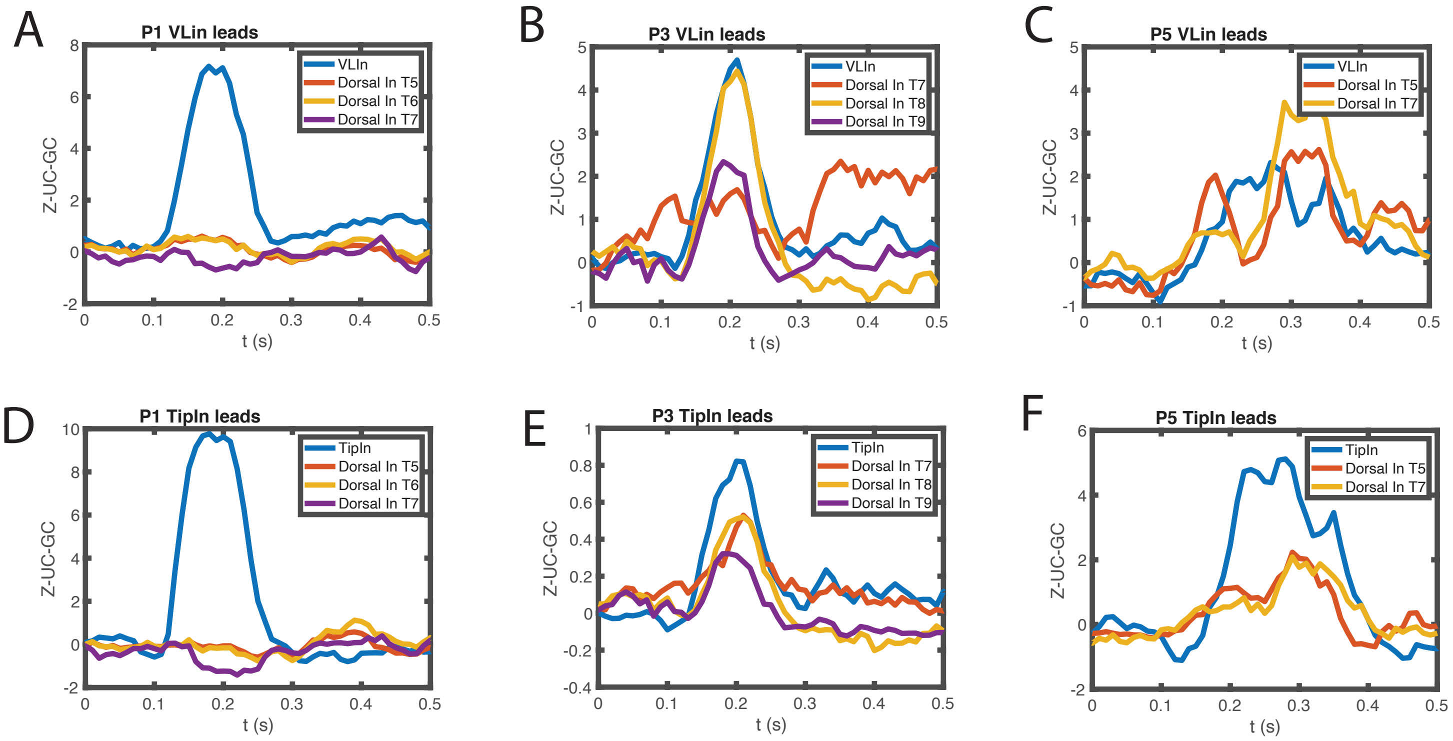

A
P1 VLin leads
VLIn
Dorsal In T5
Dorsal In T6
Dorsal In T7
Z-UC-GC
t (s)
B
P3 VLin leads
VLIn
Dorsal In T7
Dorsal In T8
Dorsal In T9
Z-UC-GC
t (s)
C
P5 VLin leads
VLIn
Dorsal In T5
Dorsal In T7
Z-UC-GC
t (s)
D
P1 TipIn leads
TipIn
Dorsal In T5
Dorsal In T6
Dorsal In T7
Z-UC-GC
t (s)
E
P3 TipIn leads
TipIn
Dorsal In T7
Dorsal In T8
Dorsal In T9
Z-UC-GC
t (s)
F
P5 TipIn leads
TipIn
Dorsal In T5
Dorsal In T7
Z-UC-GC
t (s)

Figure S7-1

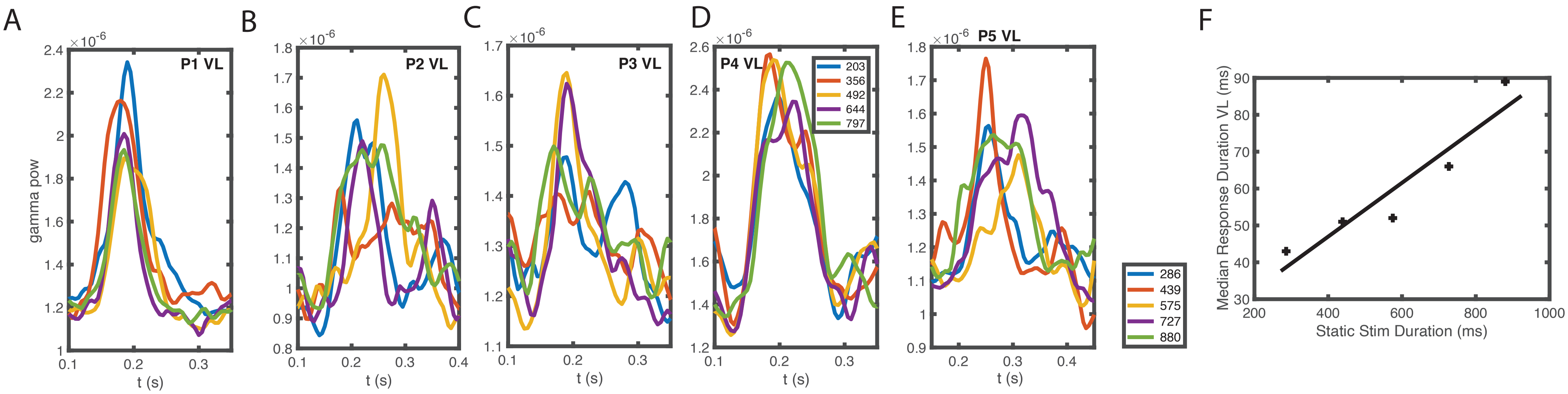
A
B
C
D
E
F
gamma pow
t (s)
P1 VL
P2 VL
P3 VL
P4 VL
P5 VL
203
356
492
644
797
286
439
575
727
880
Median Response Duration VL (ms)
Static Stim Duration (ms)

Figure S7-2

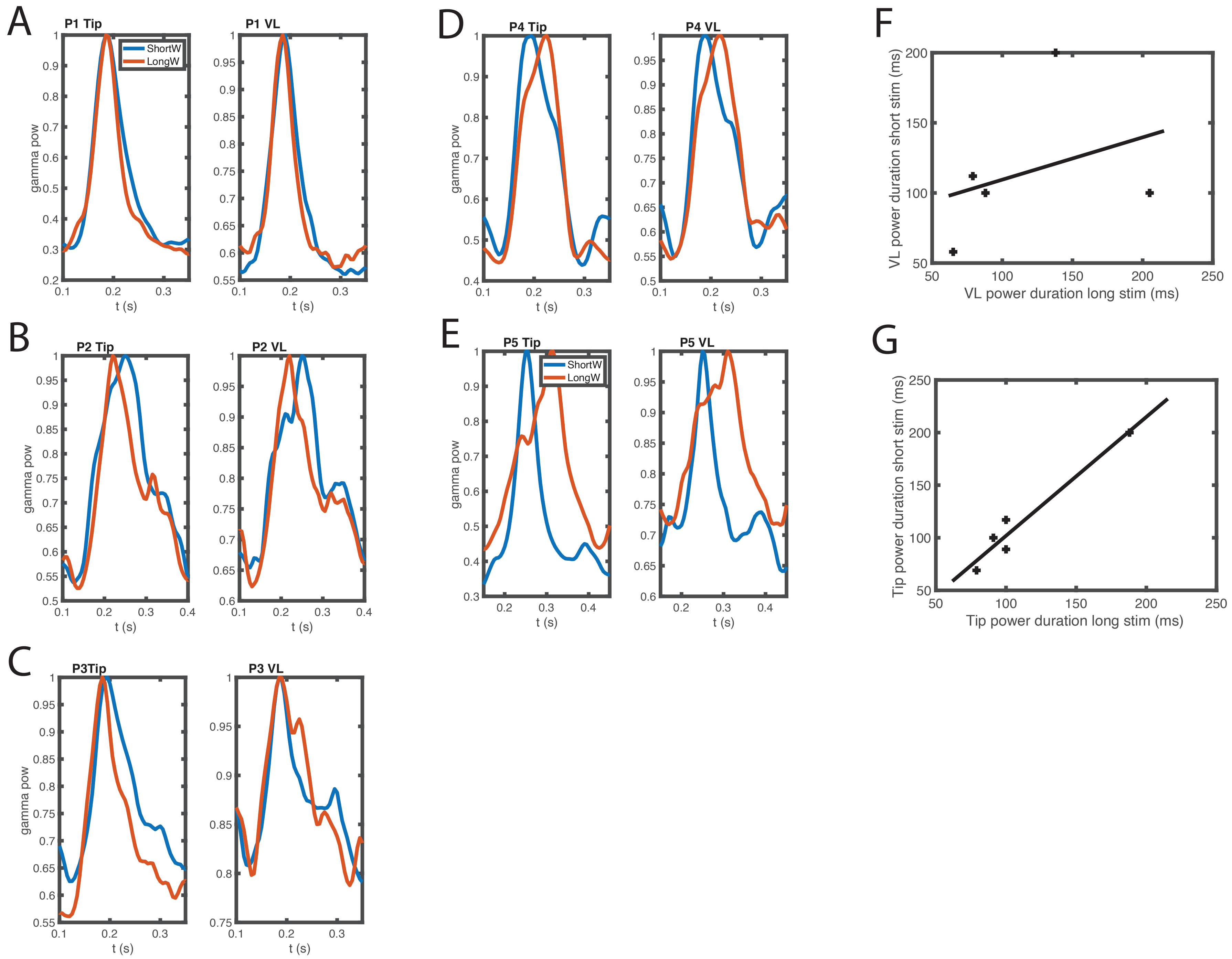

A
P1 Tip
ShortW
LongW
gamma pow
t (s)
P1 VL
B
P2 Tip
P2 VL
C
P3Tip
P3 VL
D
P4 Tip
P4 VL
E
P5 Tip
P5 VL
F
VL power duration short stim (ms)
VL power duration long stim (ms)
G
Tip power duration short stim (ms)
Tip power duration long stim (ms)

Figure S7-3

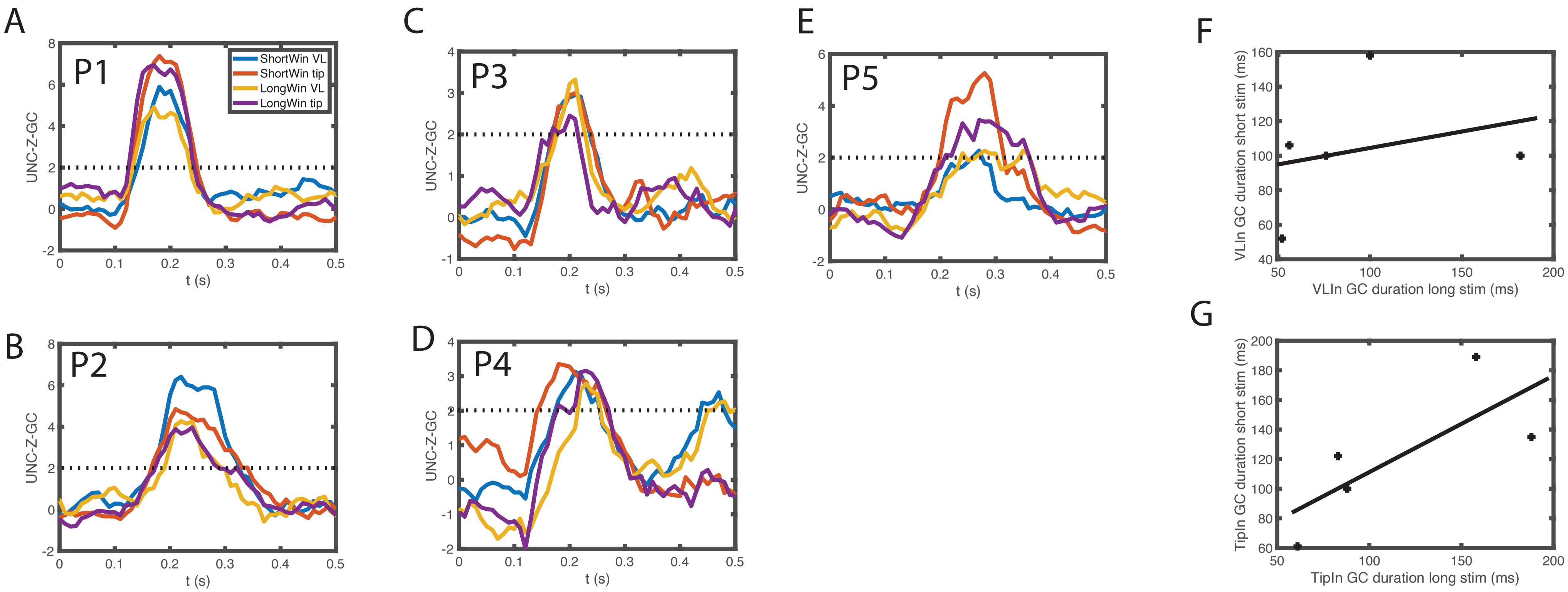
A
B
C
D
E
F
G
P1
P2
P3
P4
P5
ShortWin VL
ShortWin tip
LongWin VL
LongWin tip
UNC-Z-GC
t (s)
VLIn GC duration short stim (ms)
VLIn GC duration long stim (ms)
TipIn GC duration short stim (ms)
TipIn GC duration long stim (ms)

Figure S8-1

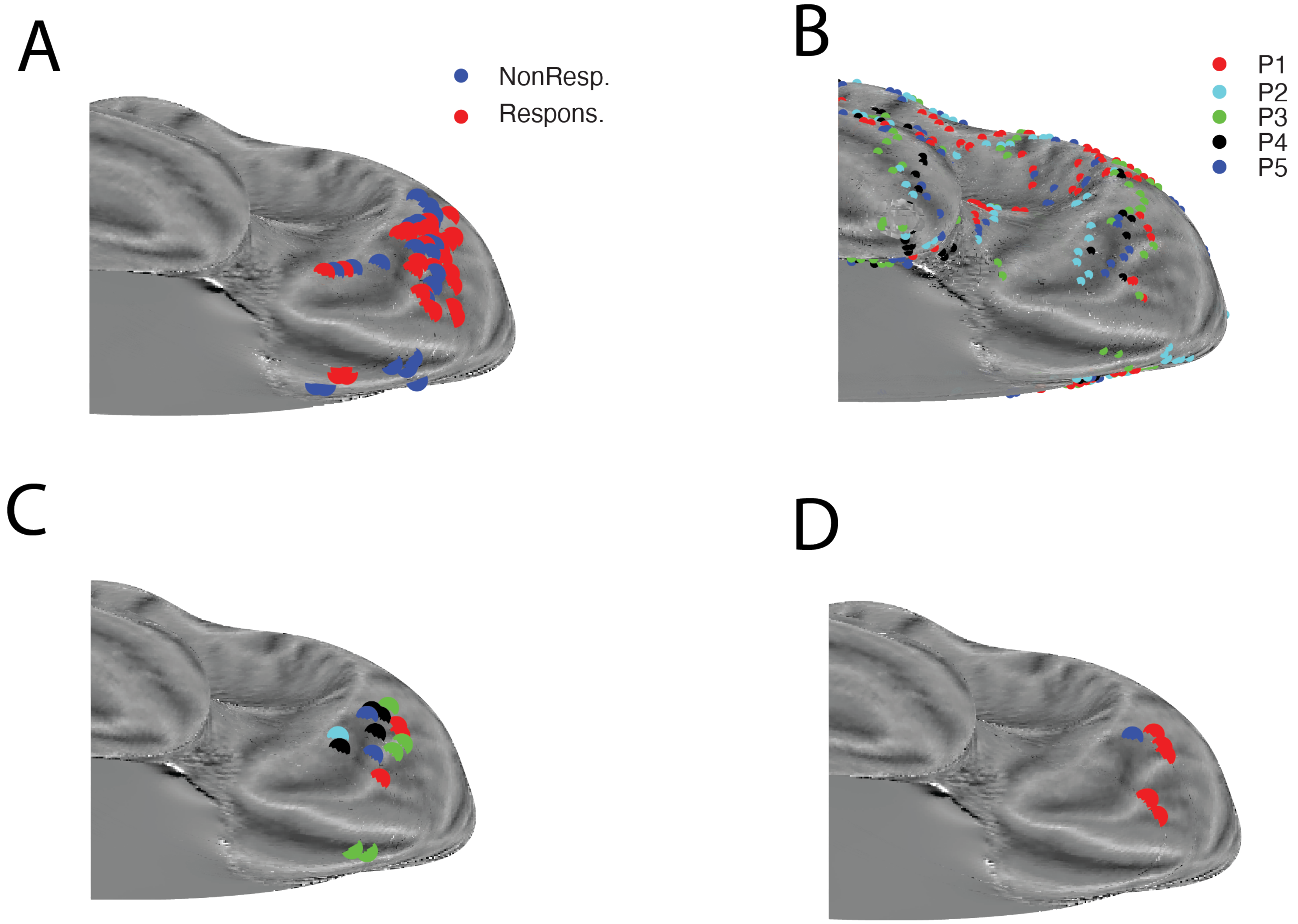

Figure S8-2

P3

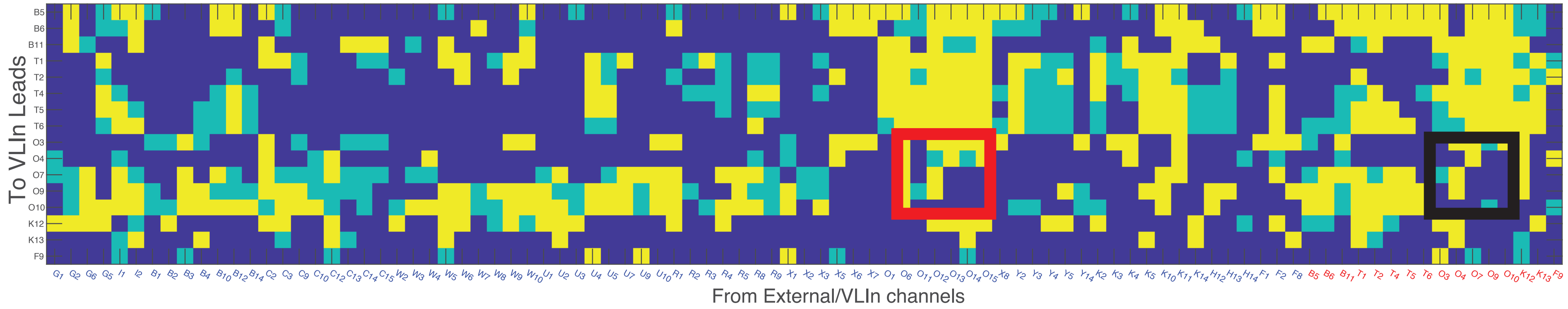

Figure S8-3

P1

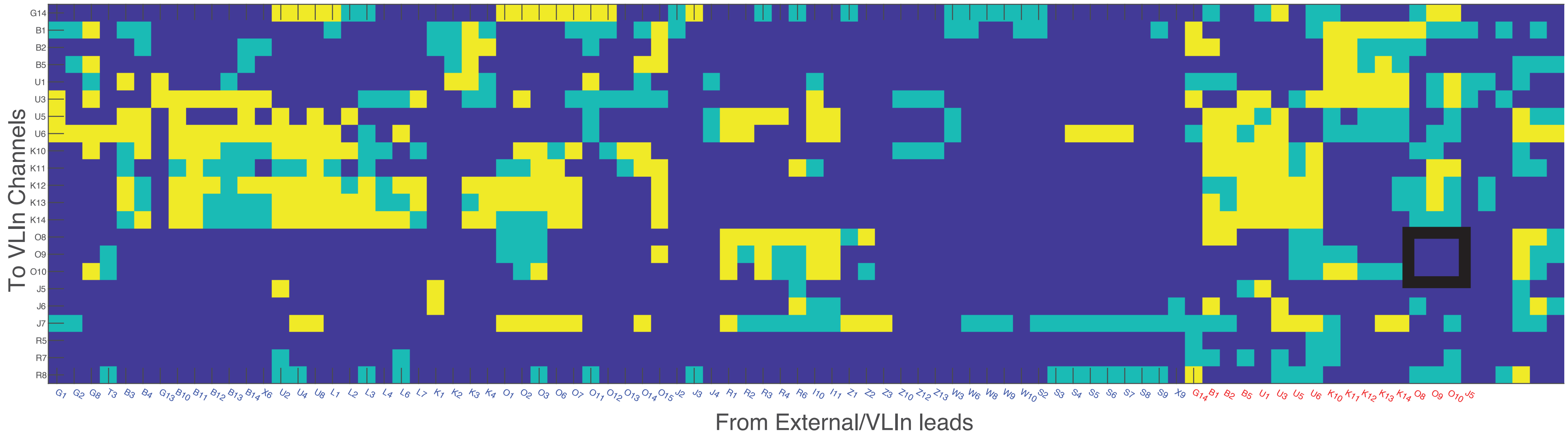

Figure S8-4

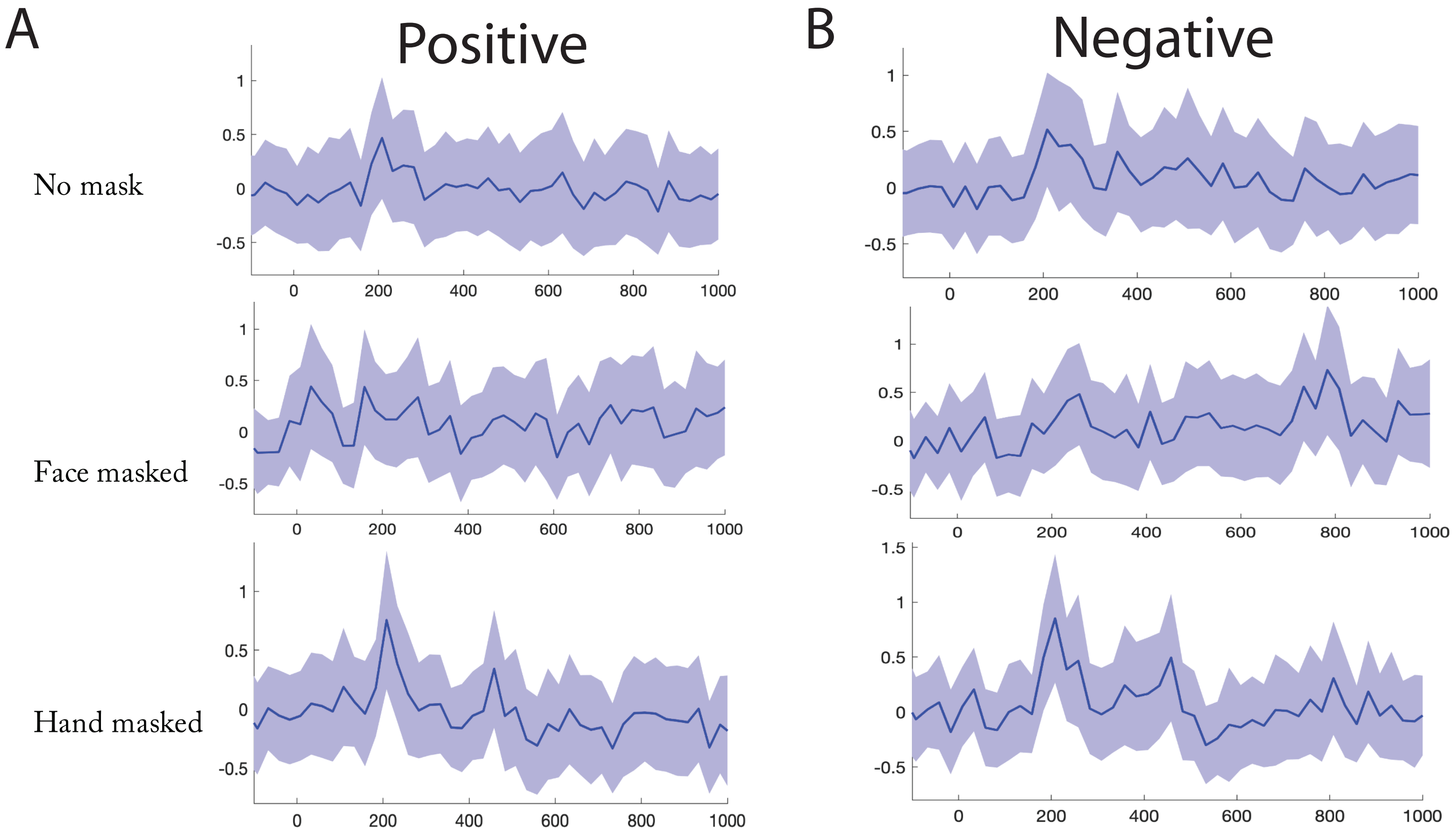

A
Positive
B
Negative
No mask
Face masked
Hand masked
1
0.5
0
-0.5
1.5
0
200
400
600
800
1000

Figure S8-5

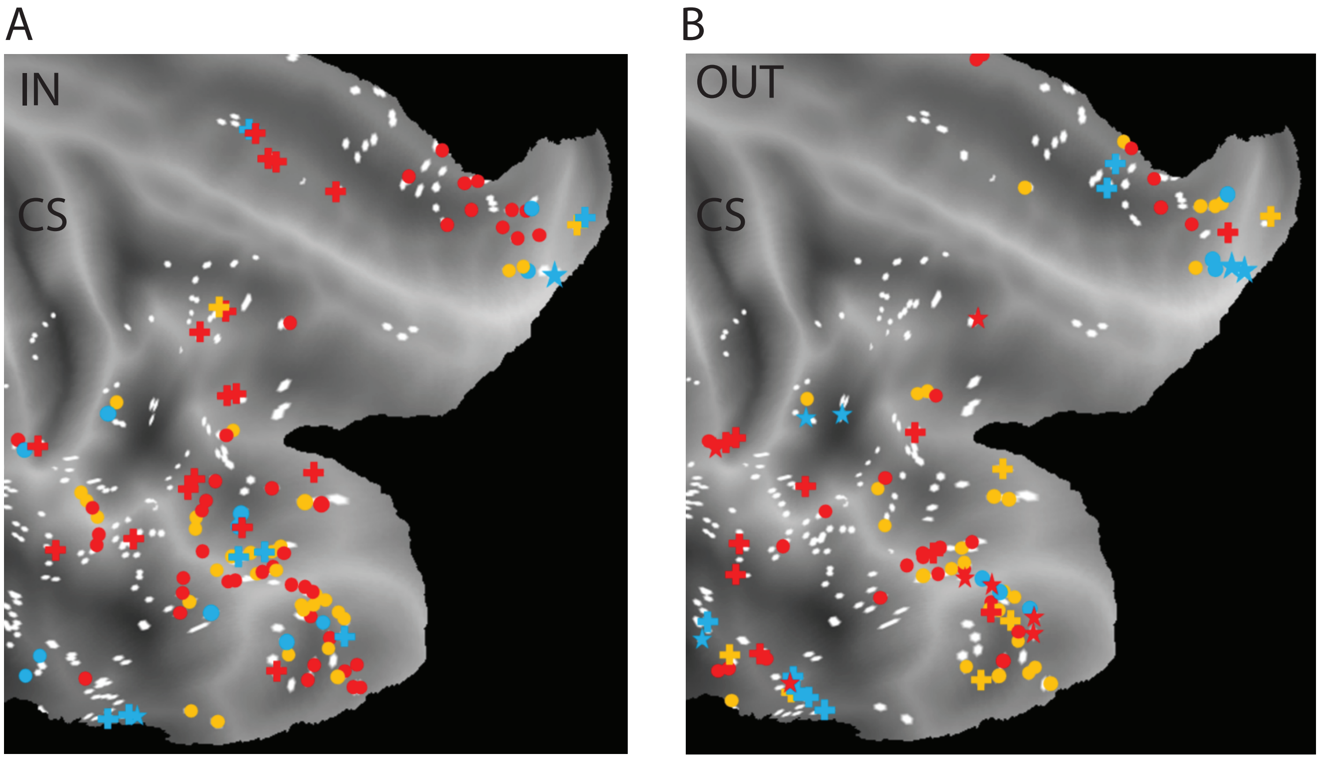

Supplement: Supplementary file 2 — Supplementary Information 2. [file 41598_2023_33318_MOESM2_ESM.pdf]
